# Deep Eutectic Solvents for Pretreatment of Lignocellulose Biomass: Physical Properties, Solubility Mechanisms, and Their Interactions

**DOI:** 10.1021/acs.chemrev.5c00597

**Published:** 2026-01-13

**Authors:** Ntebogeng Mqoni, Indra Bahadur, Sangeeta Singh, Xianzhi Meng, Arthur Ragauskas

**Affiliations:** † Department of Chemistry, Faculty of Natural and Agricultural Sciences, 56405 North-West University (Mafikeng Campus), Private Bag X2046, Mmabatho 2735, South Africa; ‡ Thermodynamics-Materials-Separations Research Group, Department of Chemical Engineering, Mangosuthu University of Technology, Umlazi, Durban 4031, South Africa; § Department of Chemical Biomolecular Engineering, The University of Tennessee-Knoxville, Knoxville, Tennessee 37996, United States; ∥ Department of Forestry, Wildlife, and Fisheries, The Center for Renewable Carbon, The University of Tennessee-Knoxville, Knoxville, Tennessee 37996, United States; ⊥ Joint Institute of Biological Sciences, Biosciences Division, Oak Ridge National Laboratory, Oak Ridge, Tennessee 37831, United States

## Abstract

As the most accessible and abundant renewable resource on earth, lignocellulosic biomass mainly consists of cellulose, hemicelluloses, and lignin with a small amount of protein, pectin, minerals, and extractives (e.g., tannins, lipids, and resins). Lignocellulosic biomass has gained extensive attention in industry and research owing to its renewability, availability, and low cost. However, achieving efficient fractionation of lignocellulose components and all-component utilization in a green and cost-effective manner remains a challenge dueto biomass recalcitrance. Deep eutectic solvents (DESs) have received considerable attention because they are biocompatible, inexpensive, biodegradable, have low toxicity, and are easy to prepare and recycle; these characteristics strongly depend on individual components involved in DESs preparation. This review systematically summarizes recent progress in the fractionation of carbohydrates (cellulose and hemicelluloses) and lignin from biomass using DESs, with particular emphasis on the effects of DES types and pretreatment parameters on fractionation efficiency. The subsequent conversion and upgrading of the DES-fractionated products (i.e., carbohydrates and lignin) are comprehensively analyzed. Finally, the challenges and future prospects of lignocellulose biomass fractionation using DESs are proposed in view of the existing limitations. This review provides an in-depth understanding of lignocellulose biomass fractionation during DESs processing, offering insights to improve current pretreatment methods and/or to explore new pretreatment methods aimed at mitigating the global energy crisis.

## Graphical abstract

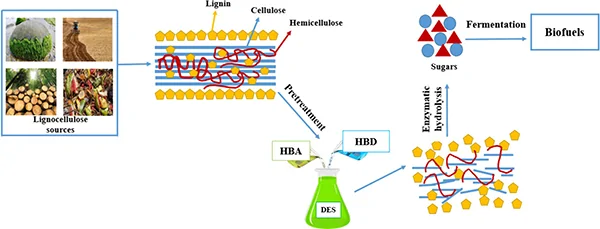

## 1 Introduction

In the 20th century, human society became highly dependent on fossilized organic resources, including coal, gas, and oil, for energy and chemical production due to the expansion of civilization and industrialization, which that requires substantial energy input.^1–3^ A challenge that the world currently faces is resource depletion owing to the exploitation of nonrenewable fossil resources, which leads to an increasing need for sustainable technologies to supply carbon-based fossil resources. Coupled with the environmental and human health concerns associated with petroleum-based materials, it is inevitable that a sustainable and eco-friendly alternative is needed. One promising solution is to develop renewable and domestically produced chemicals to target transportation fuels. An integrated biorefinery is defined by Tian et al.^4^ as “the usage of different techniques that transform lignocellulosic biomass into a variety of biofuels, material precursors and platform chemicals with the goal of complete biomass utilization.” The valorization of nonrenewable materials into biofuels and chemicals was initially explored with food crops until it was recognized as a threat to food security, as edible crops were used.^5,6^ As a potential alternative to edible crops, lignocellulosic biomass has been introduced as a renewable resource for the production of various biofuels and biobased chemicals.^7^ To further amplify its potential, lignocellulosic biomass is regarded as the most copious sustainable carbon-based resource on earth; it is attainable as forest biomass and herbaceous materials, agricultural, industrial, municipal wastes, and marine algae,^8–10^ it is carbon neutral because the biomass carbon does not contribute to the CO_2_ net input of the carbon cycle. Lignocellulosic biomass can be found at a lower cost than other carbohydrates, such as starch and sucrose, and it also has a low nitrogen and sulfur content.^11^

Lignocellulose is an organic material found in the cell walls of soft and hard woody plants, such as shrubs, trees, along with grasses and agricultural resources, and is biosynthesized by plants to serve several roles. It is primarily composed of three polymers: 35–50% cellulose, 20–35% hemicellulose, and 10–30% lignin. In addition to its primary components, lignocellulose also consists of trace amounts of other molecules such as ash, pectin, proteins, and phenolic compounds.^12,13^ Cellulose and hemicellulose make up most of the biomass, with hemicellulose cross-linked to the lignin component through covalent bonds, while cellulose forms strong hydrogen bonds within its structure to enforce a durable plant structure and provide rigidity to the plant’s framework. The percentages of the main components differ according to the type of lignocellulosic biomass, as shown in Table 1. The hierarchical structural organization of these components differs according to species origin, such as hardwood, softwood, grasses, etc.^14^ Structural differences (weight, pores, thickness, epidermal tissues, chemicals) and chemical composition (biomass composition, crystallinity, bonds)^15^ are factors that further influence the quantity of each component in each species, the structural organization, and, most importantly, the recalcitrance of the biomass toward deconstruction.

**Table 1:** Lignocellulosic Biomass Types and Its Main Constituents, Adapted from Baruah et al.^24^

Lignocellulosic Biomass	Lignin%	Cellulose%	Hemicellulose%
Poplar	29	44	20
Pine	30	42	21
Corncob	12	41	31
*Gmelina arborrea saw dust*	23.3	23	-
Corn stover	20	38	23
*Salvodora oleoides sawdust*	21.8	24	-
Soybean straw	22	34	16
Switchgrass	18	31	24
Barley straw	16	38	35
Coastal Bermuda grass	23	30	29
Rice husk	24	37	29
Napier grass	22	47	31
Wheat straw	15–20	33–40	20–25
Bamboo	20	45	24
Rice straw	12	38	32
Waste papers	1	65	13
Sugar cane bagasse	20–30	40–45	30–35
Elephant grass	28	36	24
Sweet sorghum bagasse	21	45	27

The most effective pretreatment technology that fractionates lignocellulose into its three utilizable forms, cellulose, lignin, and hemicellulose, is the solution to unlocking numerous product-integrated biorefinery operations.^4^ In an attempt to do so, researchers have introduced different pretreatment methods: biological (enzymes/fungi), physical (milling, sonification, and extrusion), physicochemical (ammonia fiber explosion, steam explosion, sulfite pretreatment to overcome recalcitrance of lignocellulose and carbon dioxide explosion), chemical (acid, alkali, organosolv, ionic liquid and ozonolysis)^16–19^ and different combinations thereof. Recently, ionic liquids (ILs) and deep eutectic solvents (DESs) have been developed for the fractionation of lignocellulosic biomass. Extensive research has been conducted on IL pretreatment of biomass owing to its favorable properties. On the other hand, DESs are used as alternatives to ILs, as the latter presents more shortcomings than desired. Abbott et al.^20^ was the first group to prepare homogeneous liquid DESs with temperatures below 100 °C. They have since gained attention and have been applied in various industries owing to their potential “green” properties, which largely depend on the nature of DES and its individual constituents. DESs are specifically employed in lignocellulose fractionation because of their ability to solubilize lignin, facile and inexpensive preparation, recyclability, and environmentally friendly properties.^21^ For instance, Xu et al.^22^ estimated the cost of a DES to be 20% of that of an IL. In the same breath, Gorke et al.^23^ stated that DES constituents cost ten times less than IL components. While the influence of DES composition on solvent properties has been widely explored, there is little literature on how compositional differences influence functional performance.

In this review, an extensive summary of recent advancements in DES pretreatment and the subsequent valorization of lignocellulosic biomass is presented. It also provides insight into DESs’ physicochemical properties, synthesis, various applications in biorefinery, and DES properties that affect their effectiveness in biomass pretreatment and the mechanism of action. Moreover, this work reviews the enzymatic hydrolysis of DES-pretreated substrates, integrated pretreatment methods to enhance DESs’ performance, DESs recycling, and finally, the challenges and future prospects of biomass pretreatment by DESs.

## 2 Lignocellulosic Biomass

### 2.1 Cellulose

Cellulose is a structurally linear, unbranched biopolymer composed of repeating units of glucose. It is the primary structural component of the plant cell walls, especially abundant in the secondary cell wall of plants. With more than 100 years of intensive research and development studies, structural analysis methods such as electron microscopy, X-ray diffraction (XRD), and high-resolution nuclear magnetic resonance (NMR) have concluded that the native structure consists of long-chain repeating glucose monomers, as shown in Figure 1, linked together through β-1,4-glycosidic bonds arranged in parallel.^25^ It exists in four major crystalline forms: I­(I_α_ and I_β_), II, III, and IV, as a result of intramolecular and intermolecular hydrogen bonding that exists between glucose units.^25,26^ Cellulose I is also known as native cellulose, which is predominant in plant cell walls and has a distribution of both crystalline and amorphous regions.^25^ The amorphous regions are more porous than the crystalline regions, allowing penetration and enhancing reactivity to enzymatic hydrolysis. Cellulose I_α_ has a triclinic unit cell and is usually rich in the cell wall of primitive microorganisms such as bacteria. Cellulose I_β_ has a monoclinic unit cell, and it is usually produced by higher plants and tunicates. Native cellulose can be converted into other forms of crystalline stable cellulose II. Cellulose II, which is found in marine algae, is a crystalline form that is formed when cellulose I is treated with aqueous sodium hydroxide. Cellulose II is the most thermodynamically stable form of cellulose. This is because it can be irreversibly transitioned from native cellulose due to the hydrogen bonds and van der Waals interactions. Cellulose III is prepared from cellulose I by treating it with cold (−80 °C) anhydrous ethylamine or liquid ammonia. Cellulose IV is formed by treating cellulose in hot (200 °C) glycerol, followed by washing with 2-propanol and water. Although cellulose can be derived from various sources, its chemical structure remains the same.^25–28^

**Figure 1:**
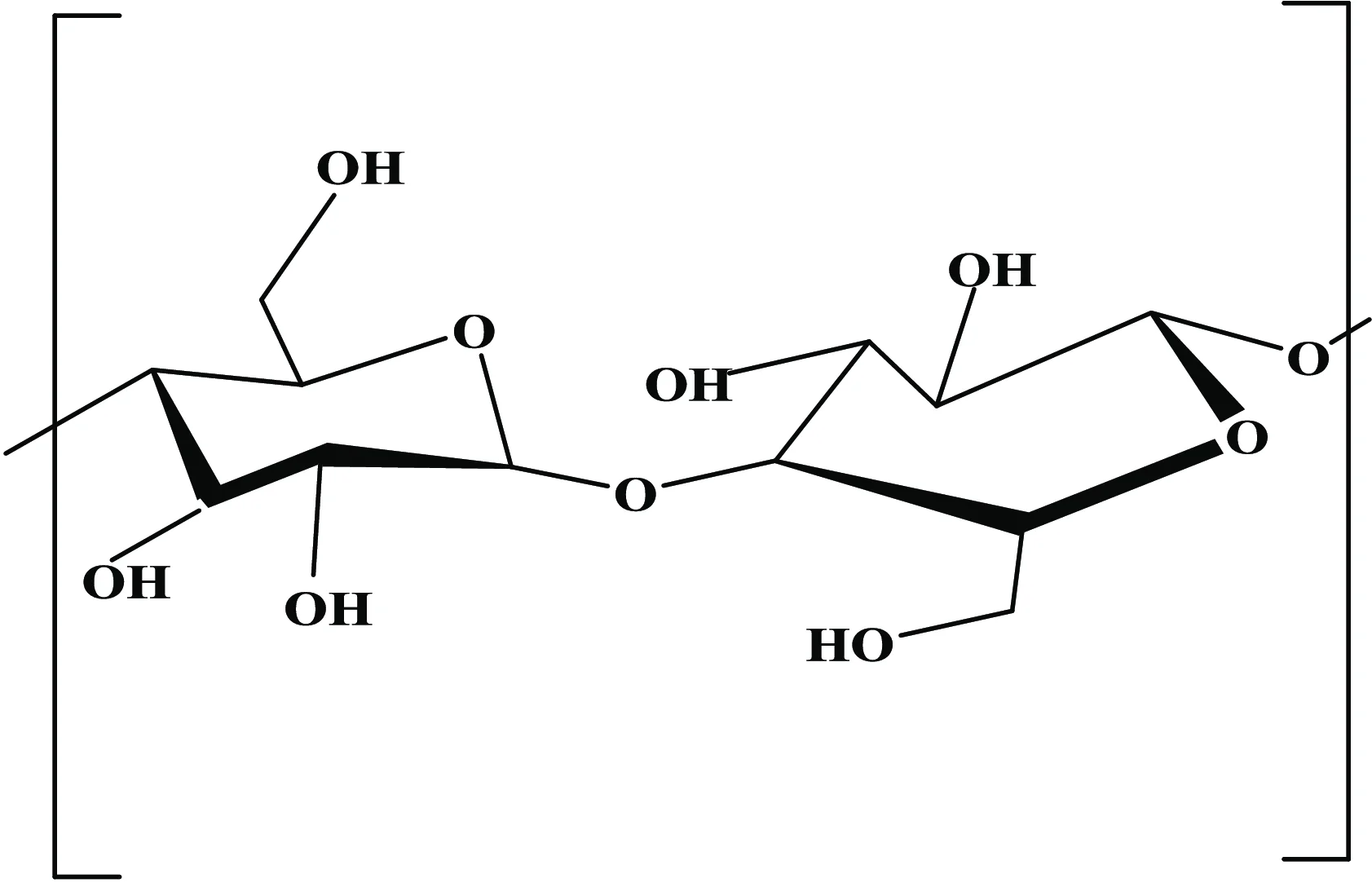
Chair conformation structure of cellulose.

Because the chains in Cellulose I are aligned in a parallel manner by hydrogen bonds, it gives rise to 3D sheets with three intermolecular forces in directions a (hydrogen bonds), b (covalent bonds), and c (weak van der Waals forces), as postulated by Holtztapple.^26^ All of these forces provide strength and crystallinity to the cellulose ultrastructure. Moreover, this compact structure and extensive hydrogen bond network are responsible for its stability, insolubility, and hydrophilicity. In the chair conformation of glucose, the hydrogen atoms are axially positioned, whereas the hydroxyl groups are in equatorial positions. The equatorial hydroxyl groups form hydrogen bonds with adjacent neighbors, facilitating crystallization, while the β-1,4-glycosidic linkages between the glucose units reinforce the extended and rigid polymer network.^26^

Being the most abundant organic polymer on terrestrial Earth, cellulose can be used in numerous applications. The following figure (Figure 2) lists the applications of cellulose, underscoring the importance of its valorization. Applications are achieved using different sources, types and derivatives of cellulose: bacterial cellulose, cellulose acetate, ethylcellulose, and hydroxypropyl cellulose (HPC).^29^ Some of these applications are still under research and development (i.e., drug delivery, textiles), others are in a pilot stage (i.e., hydrogels, cellulosic ethanol), while several have already been commercialized (i.e., paper and pulp, MCC).

**Figure 2:**
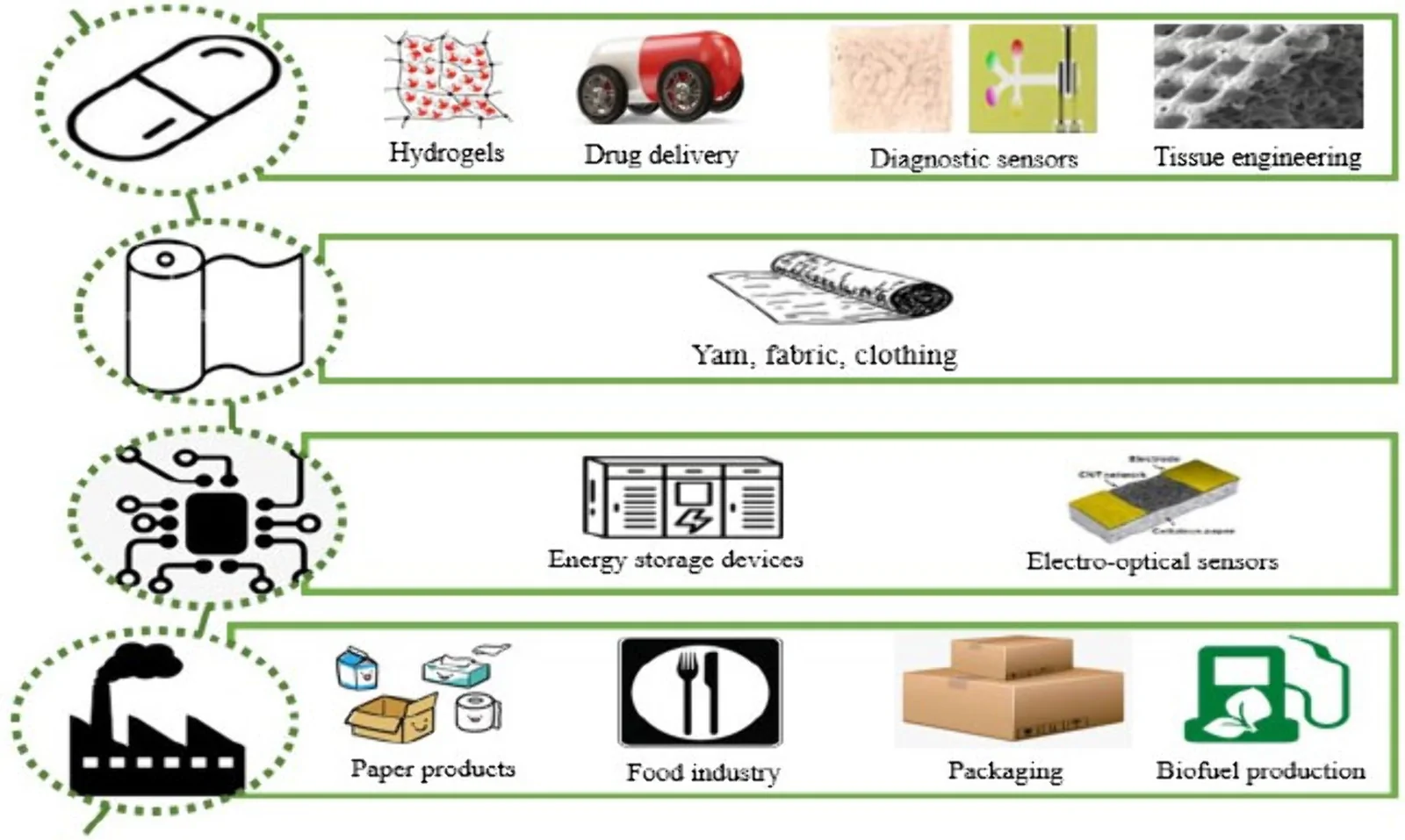
Applications of cellulose.^30^

### 2.2 Hemicellulose

Hemicellulose is the second most abundant component of lignocellulosic biomass, constituting approximately 20–35% of the plant cell wall.^31,32^ Hemicellulose adheres to cellulose through hydrogen bonds and van der Waals interactions, thus forming highly resistant networks with cellulose.^33^ Unlike cellulose, it has a heterogeneous, amorphous network composed of various short and branched heteropolymers including C6 sugars such as d-glucose, d-galactose, and d-mannose and C5 sugars such as d-xylose and l-arabinose as well as sugar/uronic acids like d-glucuronic, d-galacturonic, and methylgalacturonic acids.^1,34–36^ The composition and abundance of hemicellulose are dependent on the biomass and its nature.^31^ For example, hardwood hemicelluloses are mostly composed of xylans, whereas softwood hemicelluloses mostly comprise glucomannans.^37^ The recalcitrance of hemicellulose originates from its structure. For example, feruloyl groups attach to arabinoxylans, which can undergo oxidative coupling to form diferulate bridges that cross-link xylan chains and anchor them within the cell wall, creating a cross-linking mesh-like network, effectively embedding heteroxylans within the cellulose–lignin matrix.^38^ Furthermore, in hardwood xylans, 60–70% of xylose residues are acetylated, whereas in softwood heteroxylans, arabinofuranosyl residues are often esterified with p-coumaric acid and ferulic acid. This is why the degree of polymerization of hardwood xylans (150–200) is higher than that of softwood (70–130).^37^ Although the structure of hemicellulose is more complex than that of cellulose, it is actually more susceptible to enzymatic degradation. However, its hydrolysis requires a wider array of enzymes due to its heterogeneous nature. The highly branched and amorphous nature of hemicellulose allows for its easy conversion; it is more unstable than cellulose and thus readily degrades when subjected to heat.^39^ The valorization of hemicellulose requires the selective dissolution of hemicellulose from raw biomass and the selective production of target materials from hemicellulose derivatives. However, its valorization is inhibited by physical and covalent interactions with other cell-wall components.^39^ Biological conversion of hemicellulose into valuable products often involves complete depolymerization into monomeric sugars, although isolated hemicelluloses could also be utilized in various material applications such as films and hydrogel production, 3D printing materials, and adsorbents.^40^

Hemicellulose depolymerization can be achieved chemically (acids) or biologically (hemicellulases).^41^ Normally, hemicellulose hydrolysis requires a consortium of enzymes for complete degradation due to the variable structure and organization of hemicelluloses; different enzymes work at different levels within the hemicellulolytic matrix.^42^ Consortium action is important because of the complexity of hemicellulose and its adherence to other components. Recently, hemicellulose degradation has been extensively studied owing to its practical application in various fields. For instance, furfural (FF) is a hemicellulose degradation byproduct and is a key product in biorefinery industries that could be further employed as a fuel and chemical in plastics, oil refining, pharmaceutical, and agrochemical industries.^39^ However, FF conversion is still in its infancy. Some of the introduced pretreatments have disadvantages that inhibit the valorization of hemicellulose. Suitable pretreatments, which produce high-yield products (i.e., xylitol, FF, and organic acids) with no decomposition of cellulose and lignin, need to be developed.^39^ These pretreatment technologies should be able to minimize the formation of inhibitory compounds and be tailored to the specific characteristics of the biomass used. Most importantly, they should be scalable for industrial applications and more cost-effective than the existing conversion technologies.

### 2.3 Lignin

Lignin is the third most abundant component of lignocellulosic biomass.^43^ It is a flexible and aromatic biopolymer that is often covalently bonded to hemicellulose, thus creating a protective barrier that contributes to the recalcitrance of lignocellulose to physical and biological attacks.^44^ It exists in the tissues of vascular plants, which aids the structural, water resistance, and mechanical strength of the plant cell wall.^45^ There have been cases of lignin redeposition after pretreatment. Selig et al.^46^ reported the detection of spherical lignin droplets after high-temperature acid pretreatment due to the migration and redeposition of lignin from the plant cell wall to the liquid phase and back to the biomass surface. Lignin is also responsible for limiting cellulose swelling and controlling water intake and retention,^47^ despite it being more hydrophobic than its counterpart polysaccharides such as cellulose.^48^ Native lignin is not found isolated in nature but exists in a lignocellulose matrix.^49^ Lignin is a versatile, 3D cross-linked polyphenol macromolecule composed of phenyl propane structural units, which vary based on the substitution of methoxyl groups on the aromatic rings. These lignin subunits are primarily interconnected via aryl ether linkages (e.g., β–O–4 and α–O–4) and carbon–carbon bonds (e.g., β–5 and β–β). Lignin biosynthesis occurs mainly through combinatorial free radical polymerization involving three primary monolignols, namely coniferyl, sinapyl and p-coumaryl alcohols, leading to the formation of guaiacyl (G), syringyl (S), and p-hydroxyphenyl (H) subunits, respectively.^24,50,51^

Lignins of different origins vary in the ratio of these monolignol units. For instance, softwood is rich in G units, with small amounts of H and trace amounts of S units, whereas hardwood contains both S and G units, with only trace amounts of H units.^49^ The variation in lignin structure also depends on plant tissue, growth, nutrition, species type, climate, and ecological conditions.^52^ Furthermore, different lignocelluloses yield lignin fragments with different compositions of functional groups, monomer types, and interunit linkages. Even when the same lignocellulose was used, the extraction method used to isolate lignin impacts its structure because of the extraction conditions adapted from the process.^53^ The growing research on lignin over the years highlights its increasing recognition as a valuable and versatile biopolymer. For instance, as the only renewable raw material that consists of aromatic units, lignin depolymerization to obtain valuable aromatic compounds has been explored.^54,55^ Moreover, lignin has also been valorized into high-value-added materials (carbon fibers), as well as biofuels (multifunctional hydrocarbons and syngas);^56^ however, the complex structure of lignin and insufficiently developed pretreatment technologies hinder the practical application of lignin.^54^ Despite growing advancements in the research, development, and commercialization of lignin, its limitations are not confined to its contribution to biomass recalcitrance. Lignin also presents inherent challenges, such as the difficulties in meeting precise product purity specifications; therefore, its valorization strategies often need to target multiple commodity chemicals rather than a single product. During the isolation process, lignin frequently forms condensed structures which have inferior performance compared to synthetic chemicals in many applications, and there is a large variety of lignin types, limited availability of pure lignin, unique reactivity,^57^ energy-intensive fragmentation and purification processes that further hinder its efficient and broad utilization. All these considerations portray the need for new, efficient, mild lignin extraction protocols.

Integrated biomass biorefinery has seen varying degrees of success and failures globally. It started around 1909 ^58^ with the Skutskar sulfite plant in Sweden (33 plants in total), producing 85 000 m^3^ sulfite ethanol by 1950, using hemicellulose from spruce that was pretreated in acidic sulfite pulping and then dissolved and hydrolyzed to monosaccharides.^59^ Several sulfite plants followed, but most are no longer in operation, while the status of some is unknown.^59^ The Borregaard plant in Norway is the largest producer of second-generation ethanol from lignocellulose with a capacity of 20 000 m^3^, they have had this commercial operation for decades, spanning from paper, high-value chemicals and biofuels.^59^ While they demonstrated commercial viability and environmental performance, other projects have illustrated challenges of scaling innovative technologies and failed to achieve long-term success. In September 2014, Project LIBERTY started the first commercial-scale corn stover-derived cellulosic ethanol biorefinery in the U.S., developed by POET-DSM Advanced Biofuels in Emmetsburg.^60^ It uses ANDRITZ’s two-stage biomass treatment which involves a vertical reactor and interstage washing and second, continuous steam explosion to make sugar accessible, followed by enzymatic hydrolysis, fermentation, distillation and denaturing and finally, shipment of ethanol to refiners for blending with gasoline.^60^ Lignin was used to generate thermal power as it could not be converted to a biofuel.^60^ Even though they reached 20 million gallons per year and 770 bone-dry tons per day (tpd) nameplate, they scaled down to research and development due to economic cost pressures.^60^ Another notable example is Abengoa Bioenergy, which, despite pioneering technologies in cellulosic ethanol and waste-to-biofuel conversion,^61^ ultimately faced operational and financial collapse, accumulated unsustainable debt, and filed bankruptcy a year after official opening^61^ (not including the demonstration plant). Recently, St1 Cellunolix Biorefinery in Finland also closed due to underperformance and unprofitability; production started with a targeted annual production capacity of 10 million liters of bioethanol (from sawdust biomass) with a wide range of coproducts,^62,63^ but it only achieved a third of its targeted annual production capacity.^64^ Moreover, the availability and constant supply of sawdust from local mills were important for the plant’s operation and the lack thereof posed a challenge.^64^ It appears the success of Borregard stems from diversification instead of a single market, feedstock flexibility, integration of all biomass parts, investing in research and demonstration plants before commercialization to tune product characteristics and understanding technical, operational, financial, and market challenges. However, Borregaard involves energy-intensive processing and economic viability is not guaranteed for all products. Most large-scale biorefinery plants use steam explosion and sometimes acids. No large-scale commercial biorefinery solely uses ILs and DESs yet, but these solvents are actively tested in pilot scale such as Lixea (Sweden^65^), moving from concept to practical application, supported by ongoing research and development aimed at identifying ideal solvents for biorefinery processes.

## 3 Pretreatment Methods

Various pretreatments have been used to degrade lignocellulosic complexes. These are divided into four categories: biological, physical, chemical, and physicochemical,^11,66,67^ as shown in Figure 3. Pretreatment methods aim to disrupt the physical and chemical structure of biomass, to enhance the accessibility of cellulose, while some methods may partially fractionate biomass into its main components: lignin, hemicellulose, and cellulose. That is facilitated by altering the structural features and thus altering the factors (e.g., biomass porosity, cellulose DP and crystallinity, lignin structure and content) that contribute to biomass recalcitrance.^11^ In essence, the optimum pretreatment should achieve the following objectives as described by Sasmal et al.:^11^

- A high yield of the cellulose fraction that can be readily hydrolyzed into monomeric sugars via biological deconstruction.
- Little or no production of inhibitors that reduce the sugar production
- Fractionate each biomass component with minimal product loss
- Require minimal energy
- Require fewer chemicals and operational costs

**Figure 3:**
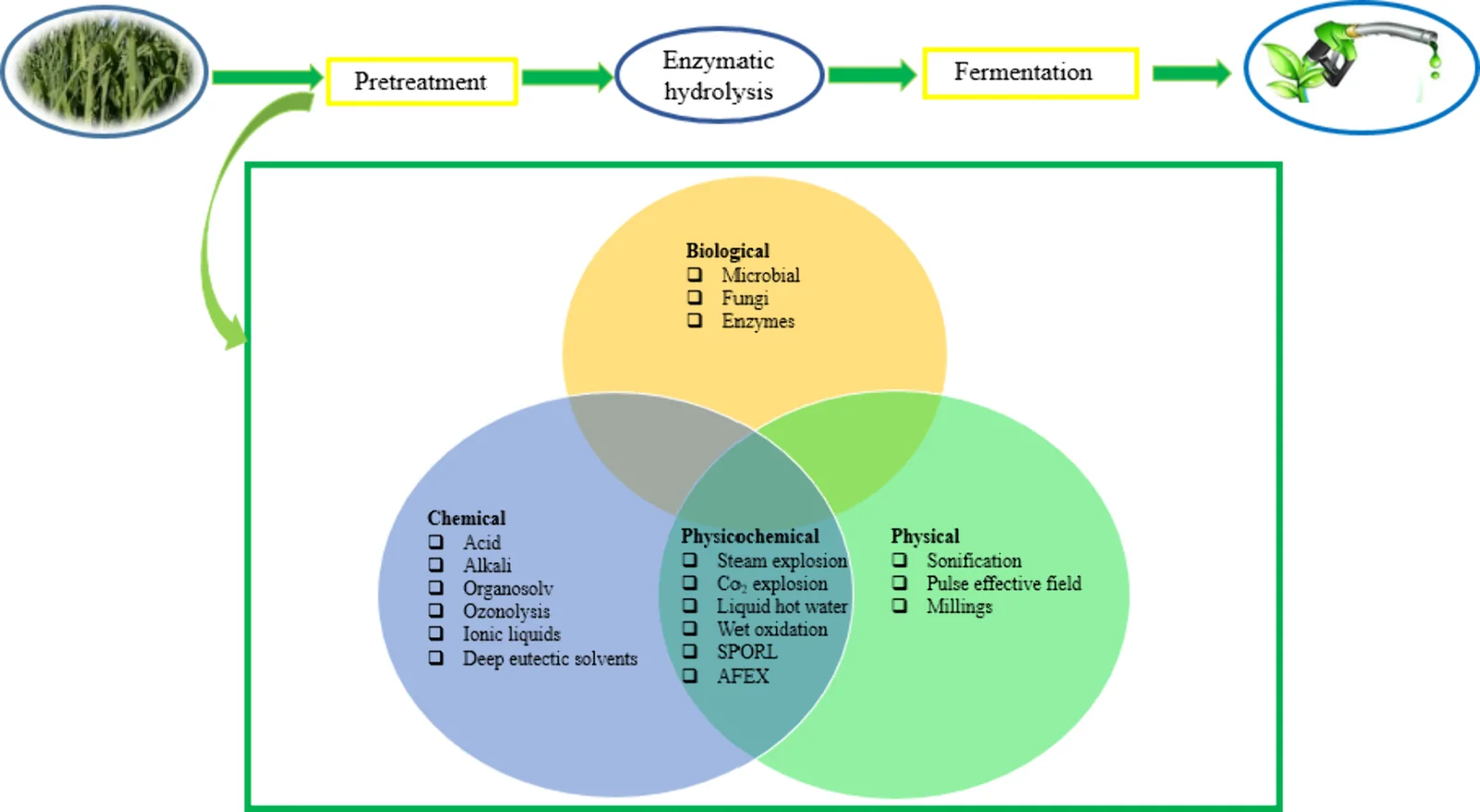
Biomass pretreatment methods that have been presented over the years.^11,66,67^

The choice of the optimum pretreatment method and conditions depends on the objective/desired product, environmental, and economic impact; therefore, it is almost impossible to define the best pretreatment.^68^ However, the product should be economically competitive and environmentally friendly. Each pretreatment method has advantages and disadvantages, which are listed in Table 2. The efficiency of each pretreatment process in obtaining a good fuel yield is influenced by its ability to fractionate the biomass components. Each of these methods has a distinct impact on the biomass composition.

**Table 2:** Advantages and Disadvantages of Lignocellulosic Pretreatment Methods^17–19^

Pretreatment Type	Pretreatment Method	Advantages	Challenges
Biological	Enzyme/Fungi	Minimum energy. Environmentally friendly, recycling is not required. No byproducts. Degrades both low and high moisture containing biomass	Requires to be carried out in sterile conditions. It is too slow. Degrades lignin to some extent
Physical	Milling	Reduces the crystallinity and degree of polymerization	Requires and consumes high energy
			Incapable of delignification
	Sonification	Easy pretreatment, high heating capacity, short pretreatment duration, less production of inhibitors, minimum energy use	Requires biomass comminution
	Pulse electric field (PEF)	Simple design and short pretreatment time, saves energy	Electrode corrosiveness can depreciate the quality of extracted chemicals
Physicochemical	Ammonia Fiber Expansion(AFEX)	Lower moisture content, less production of inhibitors, 100% guarantee of solid material recovery	High costs
			Does not delignify the biomass
	Steam explosion	Less use of hazardous chemicals, high energy efficiency and low environmental impact, short duration, cost-effective	Produces inhibitors
			Incomplete disruption of lignin
	Carbon-dioxide explosion	Uses low temperature, increases the accessible surface area	Does not alter lignin and hemicelluloses, high costs
	Liquid hot water (LHW)	Nonuse of chemicals and catalysts and no production of inhibitors, does not require expensive reactor machines.	Consumes high energy due to higher pressures and a large quantity of water
	Wet oxidation	Allows large particle sizes to process up to 50% substrate loading	High cost of hydrogen peroxide, generation of undesirable byproducts resulting from oxygen-based oxidation reactions
	Sulfite Pretreatment to Overcome Recalcitrance of Lignocellulose (SPORL)	Produces a small number of inhibitors, reduces the energy consumption required after comminution, has scalability	Commercial scalability needs to be demonstrated
Chemical	Strong Acid	No requirement for subsequent enzymatic hydrolysis, efficient degradation upon hemicellulose and lignin solubilization	Production of inhibitors, extensive washing and detoxification, necessitates rector materials capable of withstanding acid corrosion
	Alkali	Low temperature and pressure, allow access of cellulose and hemicellulose to enzymes, the lime pretreatment is less costly	Longer pretreatment time, the efficacy of sugar degradation is lower, CO_2_ can react with lime to form CaCO_3_
	Organosolv	Satisfactory separation of cellulose with minimal degradation, produces high-quality lignin	Several organic solvents used are volatile and flammable.
	Ozonolysis	Uses ambient temperature and pressure. No requirement for chemicals. No use of liquids, thus avoiding product dilution.	Highly reactive, flammable, and corrosive. It is very costly as large ozone is required and requires cooling systems

Unlike other conventional methods that use high energy and require expensive reagents and equipment, biological pretreatment uses live microorganisms to convert lignocellulosic biomass to bioenergy products.^69,70^ As lignocellulosic biomass is a complex structure with various carbohydrate polymers and lignin, biological deconstruction and conversion require a multitude of enzymes that degrade each of the existing components of biomass differently. With physical pretreatments, the biomass is either comminuted into smaller particles using mechanical comminution or the biomass specific surface area is increased by subjecting it to shear-induced “fibrillation,” otherwise known as mechanical refining.^71–73^ With an increased external surface area, cellulases have greater access to biomass, thus enhancing the hydrolysis process.^66,72^ Therefore, physical pretreatment methods are often considered important steps prior to or after chemical or biological methods. Most of these methods have been observed to alter lignin structure.^71^ However, one of the notable disadvantages of these methods is their inability to extract lignin without degrading cell wall carbohydrates, which limits the accessibility to cellulose.^71^ Physicochemical methods combine chemical and physical processes, maximizing the beneficial effects while minimizing detrimental reactions. They play a critical role in enhancing cellulose accessibility for hydrolytic enzymes.^68,74,75^ Pretreatment methods depend on the process conditions and the solvent used, which in turn affect the physical and chemical properties of the biomass.^68^ Under chemical pretreatment, the methods rely primarily on chemical reactions for lignocellulosic fragmentation. Chemical pretreatments have spiked in interest due to their ability to enhance the biodegradability of cellulose by removing lignin and/or hemicellulose and/or decreasing the degree of polymerization and crystallinity of cellulose.^27,76,77^ Moreover, some of the reported chemicals are environmentally friendly as they do not produce toxic residues and could be applied at room temperature and pressure.^76^ These chemicals include oxidizing agents, alkali, organic acids, salts, and organic solvent/inorganic acid mixtures. The latter is efficient in breaking the internal lignin and hemicellulose bonds, whereas alternative treatments are used to degrade (or solubilize) lignin, hemicellulose, and cellulose.^68,78^

In response to the growing global emphasis on sustainable development, it is essential to incorporate environmental performance criteria into the evaluation of lignocellulosic pretreatment methods,^79^ aligning with bioeconomy goals and principles. While these techniques are traditionally assessed for their effectiveness in fractionating biomass and enhancing enzymatic digestibility, they are also appraised through a sustainability lens using frameworks such as Life Cycle Assessment (LCA) and techno-environmental analyses to determine the overall feasibility and eco-compatibility of a given method.^80^ These assessments consider environmental metrics such as energy demand, greenhouse gas emissions, toxicity, waste generation, and biodegradability of residues and byproducts formation. Table 3 provides a comprehensive comparative overview of selected methods based on these environmental metrics. This holistic evaluation enables informed selection of pretreatment strategies not only based on technical effectiveness but also on long-term environmental performance. The metrics consider both direct and indirect environmental impacts. Using both Tables 2 and 3 could help with the inherent trade-off of each method. For example, biological pretreatments score high across most environmental categories, but they are often limited by long reaction time, the need for sterile conditions and partial delignification.

**Table 3:** Comparative Environmental Assessment of Lignocellulosic Biomass Pretreatment Methods

Pretreatment Type	Method	Energy Use	Emissions	Toxicity	Waste/Byproducts	Biodegradability/Sustainability
Biological	Enzyme/Fungi	Very low	Negligible	Very low	Minimal	Excellent
Physical	Milling	High	Low to moderate (electricity)	Very low	Minimal	High (no chemicals, but high energy input)
	Sonication	Moderate	Low	Low	Minimal	High (no reagents used)
	PEF	Low to moderate	Very low	Very low (corrosiveness)	None	High (nonthermal, no reagents)
Physicochemical	AFEX	Moderate to high	Low (if ammonia is recycled)	Moderate (ammonia toxicity)	Minimal	Moderate
	Steam Explosion	Moderate	Moderate	Low (if no chemicals added)	Moderate inhibitor formation	Good (no chemicals used)
	CO_2_ Explosion	Moderate (pressurization)	Very low	Low	CO_2_ is recoverable	High (solvent-free, recyclable gas)
	Liquid Hot Water	Moderate to high	Low to moderate	Low	Low; mostly hemicellulose sugars	High
	Wet Oxidation	High	Moderate to high	Moderate (oxidants involved)	Inhibitors may form	Low to moderate
	SPORL	Moderate to high	Moderate	Moderate (sulfite toxicity)	Sulfite-containing liquor	Moderate (with recovery systems)
Chemical	Acid	High	High	High (corrosive, hazardous)	High; inhibitors and acidic waste	Poor
	Alkali	High	High	Moderate to high (caustic)	Alkaline effluents	Low to moderate
	Organosolv	High	High (if solvent recovery is poor)	Moderate (flammable, volatile)	Solvent waste if not recycled	Moderate

## 4 Deep Eutectic Solvents

With respect to biorefinery and biomass dissolution, ILs are the often-costly solvents used in research and development. Many ILs are organic salts that are liquids at room temperature (melting point <100 °C).^81^ Owing to their chemical variety, ILs have been further divided into subclasses, but they all inclusively comprise ions: a bulky organic cation and an inorganic/organic anion, which are poorly coordinated.^1^ However, alternative IL formulations do exist. For example, recent research shows that ILs can be formulated based on ethoxylation-induced melting point depression using simple, low-toxic, and inexpensive ethoxylated anions (C_*x*_(EO)_*y*_CH_2_COO^–^) to liquefy various metal ions, regardless of charge.^82^ ILs possess favorable chemical and physicochemical properties that merit their use as pretreatment solvents for lignocellulose. Although advancements in ILs as green solvents have been promising, their toxicity, cost, biodegradability, and recycling challenges are important issues that often prohibit their large-scale use. Researchers have attempted to develop methods to amend these shortcomings.^83^ In 2003, Abbott et al.^20^ defined and coined the term ‘deep eutectic solvents’ (DESs) after examining the heating and resulting freezing points of a range of quaternary ammonium salts with ZnCl_2_. They discovered that using choline chloride as the ammonium salt resulted in the lowest melting point of 23–25 °C and since then, a variety of DESs have been developed.

DES definition often has terminological inaccuracy in literature, where mixtures of HBAs and HBDs that exhibit a reduced melting point are referred to as DESs. However, the proper use of the term requires mastery of the solid–liquid phase equilibrium to accurately determine the true eutectic composition.^84^ A DES is a homogeneous mixture composed of two components: a hydrogen-bonding donor (HBD) and a hydrogen-bonding acceptor (HBA), which establish a joint superlattice at a definite molar ratio.^85^ The components of DESs can be extended to three or more.^86^ At the eutectic temperature, the joint superlattice melts.^85^ The eutectic temperature has to be lower than the melting points of the respective solid-phase components, which is due to the charge delocalization from the HBA to the HBD^20,87^ through the amplified power of the hydrogen bonds formed at a eutectic point balanced via power lessening of several more cohesive interactions, the energy lattice of a HBD and HBA, the manner in which the HBD and anion bond, and the changes in entropy upon the formation of DES.^87^ Given that the eutectic temperature is lower than the melting points of the individual components, as shown in Figure 4, the DES is regarded as deep because of the deep crevice at the eutectic point on the melting point curve.^85^ For instance, Liu et al.^88^ provided clear evidence for polyethylene glycol/oxalic acid (PEG 400/OA) DES formation through comprehensive thermal and spectroscopic analysis, DSC demonstrated a substantial depression in melting point (−63.94 °C) relative to PEG 400 (−10.37 °C) and OA (98 °C), at 0.5 molar fraction, validating the formation of a true eutectic system.^88^ Building on this, future DES pretreatment studies should likewise measure the degree of the melting point depression to verify that mixtures of two or more components exhibit lower melting points than their individual components.

**Figure 4:**
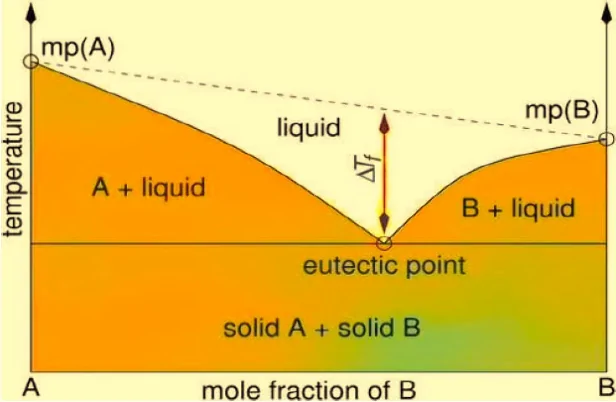
Phase diagram of the formation of eutectic mixture DES and the eutectic point.^89^ Reproduced from ref 89 with permission from the American Chemical Society. Copyright 2014 American Chemical Society.

DES can be described by the general formula: Cat^+^X^–^zY, where Cat^+^ represents any ammonium, phosphonium, or sulfonium cation; X^–^ represents a Lewis base, usually a halide ion; and z is the number of Y molecules that interact with the anion.^89,90^

### 4.1 Types of DESs

DESs are categorized into five classes based on the nature of their complexing agents, as summarized in Figure 5. Type I DESs are considered analogous to the well-studied metal halide/imidazolium salt systems.^89^ In type I DESs, there is a chloride transfer process in which excess chloride ions from the quaternary ammonium salt form complexes with its counterpart, the metal halide.^91^ Subsequently, they form metal–chloride complex stoichiometries that are not available in the pure metal chloride salt. All type 1 DES properties are influenced by this chloride-transfer behavior. However, there is a limited range of nonhydrated metal halides with a suitably low melting point compared to that of type I DESs. When using hydrated metal halides with quaternary ammonium salts instead, it gives us type II DESs.^89^ Hydrated metal halides are not air/moisture sensitive, are widely available, and are relatively less costly, which makes them desirable in industrial applications.^89^ The hydration of the metal salt results in a lower melting point compared to that of the anhydrous salt due to the decrease in the lattice energy.^89^ Therefore, the hydrated metal halides are susceptible to forming mixtures with quaternary ammonium salts that are at ambient temperatures compared to nonhydrated metal salts.

**Figure 5:**
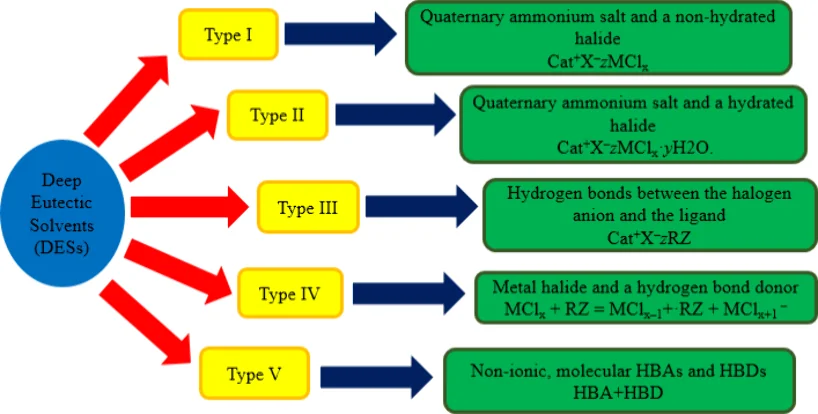
Types of DESs.

Unlike type I and II DESs, where the formation is influenced by a new anion with charge delocalization through the covalent bond between the halogen anion and the ligand, type III DESs are formed owing to the existence of hydrogen bonds between the halogen anion (HBD) and the ligand (quaternary ammonium solids).^89,92^ Among the DES types, type III is the most studied and they are usually based on choline chloride (ChCl) and various hydrogen bond donors. This is because ChCl is relatively cheap, biodegradable, has low toxicity (depending on the nature and concentration of HBD^89^) and it has been used as a natural additive for animal feed.^93^ A variety of (ChCl)-based DESs have been developed, and a range of HBDs have been employed, including amides, carboxylic acids and alcohols. Additionally, compounds such as sugars, sugar alcohols, and amino acids can be used as components of natural deep eutectic solvents (NADESs).^94^ Recently, researchers have incorporated hydrophobic compounds and long alkyl chain alcohols and carboxylic acids as hydrogen bond acceptors and donors, respectively, and these are termed hydrophobic DESs.^95^ When a DES is made with an active pharmaceutical ingredient such as ibuprofen or phenylacetic acid, it is called a pharmaceutical DES.^96^ The HBD used to prepare type III DESs greatly impacts the physical properties, and the combination with choline chloride regulates the function it serves.^97^ Owing to the wide range of availability of HBDs, these DESs can be designed for desirable applications. While type III DESs are often highlighted for their nonreactivity with water, biodegradability, cost-effectiveness, and ease of preparation,^97^ these properties should be considered within the context of their specific applications. A realistic appraisal of their limitations is important for practical application. For example, water can interact strongly with DES components, which can significantly alter properties such as viscosity.^89^ Moreover, their hygroscopic nature may affect their properties and necessitate specialized storage.^98^ Additionally, the thermal instability of some DESs and their components may pose a challenge in their preparation and application.^99^ These challenges can offset the perceived benefits of DESs and require careful consideration.

Type IV DESs have scarcely been studied, and very few of these types have been reported and are limited to metal halide-based systems. Essentially, inorganic cations do not form low-melting-point eutectics because of their high density; hence, organic cations are usually used to form ionic liquids at ambient temperature. However, previous studies have reported that the combination of metal halides and urea can form eutectics with a low melting point of <150 °C.^100^ Abbott et al.^101^ extended on this discovery and later reported that a range of transition metals can be used in ambient temperature eutectics, and the resulting eutectics were classified as type IV DESs. Therefore, these metal-cationic complexes have been used in various applications.

Type-V DESs have recently been developed. They comprise solely nonionic molecular HBAs and HBDs, which are characterized by strong negative deviations from thermodynamic ideality.^102^ Unlike the hydrophobic design of types I–IV DESs, the type V design is limited to the incorporation of large apolar ions; therefore, there is a wide range of nonionic precursors that can be used to prepare type V DESs with varying degrees of hydrophobicity compared to their ionic counterparts.^103^ These overcome the drawbacks associated with large quaternary ammonium salt-based hydrophobic eutectics, such as hydrophobicity, viscosity, and high costs. While some nonionic DESs can still exhibit high viscosities due to hydrogen bonding, the absence of ionic species generally enables lower viscosities and the presence of non-negligible vapor pressures,^104^ which enhances their use in organic and metallic solvent evaporation applications and also allows for potential recovery and regeneration of the eutectic by evaporation,^104^ a property that ionic DESs lack. The melting point characteristics of DESs are usually attributed to their ionic nature. However, type V DESs lack an ionic nature but exhibit the same properties as other DES types, which is due to the prevalent hydrogen bonding thought to be present in type V DESs.^105^ Despite the advancement and potential, literature does not go beyond the simple pairwise interaction of HBD and HBA.

Although with a small distinction to DESs, NADESs are considered a derivative of DESs instead of a type of DESs. NADESs are a class of multimolecular solvents that comprise a hydrogen-bond network formed between an HBD and HBA. This term was coined by Choi et al.^106^ to represent eutectic mixtures of two or more natural compounds, including choline chloride, citric acid, malic acid, maleic acid, acetic acid, glucose, fructose, sucrose, trehalose, and water.^107^ They are called ‘’natural’’ because their eutectic constituents are usually primary metabolite groups naturally used by the plant for survival such as sugars, organic acids and bases and amino acids.^94^ NADESs are categorized into five classes according to the nature of the constituent used in their synthesis:^94,108^

i. Ionic liquid type: made from an acid and a base
ii. Neutral type: made from sugars and/or sugar and polyalcohol
iii. Neutral-acid type: formed with sugar/polyalcohol and an organic acid
iv. Neutral-basic type: prepared with sugar/polyalcohol and an organic base
v. Amino-acid type: contains amino acids and sugar/organic acid

NADESs are considered to be environmentally friendly, green solvents that have desirable physicochemical properties such as liquidity over a wide temperature range, negligible volatility due to low pressure. These properties make them reuseable and recyclable, with the ability to decompose without the production of harmful substances. In addition NADESs are nonflammable, readily available, renewable, generally nontoxic, and biocompatible, which warrant its use in the pharmaceutical and food industry. They also exhibit thermal and chemical stability, low cost, stability at high temperatures, and ease of preparation using low energy, suitable polarity, extraction effieciency comparable to volatile organic compounds (VOCs), and tunability owing to numerous combinations of HBAs and HBDs. Although ILs, DESs, and NADESs have similar physicochemical properties, they are promising alternatives with improved environmental profiles than conventional solvents. However, the options to fit the desired application should be carefully examined.

Recently, a distinct DES category, quasi-hydrophobic DESs, relevant to biphasic extractions has emerged. They are formed from one polar (ionic or surfactant) and one apolar (surface-active or long-chain) components. Their stability is limited in aqueous or organic phases.^109^ The intermediate polarity bridges the gap between fully hydrophilic and hydrophobic DESs, offering selective solvation properties useful in targeted extraction and separation processes.^110^ Examples include a quaternary ammonium surfactant; benzalkonium chloride with octanol or other long chain alcohols^110^ and systems built from terpenes/fatty acids with small ionic or nonionic HBAs,^111^ which cause limited water miscibility due to the amphiphilic or partially apolar constituents that can solubilize moderately hydrophobic analytes while retaining some affinity for polar phases. Due to their nature, quasi-hydrophobic DESs have been explored in extraction applications such as dispersive liquid–liquid microextraction (DLLME),^110^ ultrasound-assisted microextraction,^112^ pesticide extraction,^113^ pharmaceutical residues,^114^ dyes^109,115^ and organic/inorganic analytes^116^ from aqueous matrices. Their excellent extraction performance is attributed to the formation of micellar or aggregate structures in surfactant-based DESs, which provide apolar cores for solute partitioning,^110^ improving kinetics for analytes; tunable polarity achieved by adjusting the HBA/HBD that allows fine control over solvent–solute interactions;^111^ and partial miscibility that facilitates mass transfer and dispersion compared to hydrophobic DESs.^110^ However, one notable shortcoming of quasi-hydrophobic DESs is component leaching upon water contact as the polar constituent of DES can dissolve in water over time,^117^ therefore changing the extraction performance and inhibiting DES reuse. However, this can be avoided by using lower-leaching components such as bulky HBAs, long-chain hydrophobes or polymerizable components when recycling.

### 4.2 Preparation of DESs

Researchers usually use two main methods to prepare DESs: the heating and grinding method.^118^ Experimentally, DESs are generally prepared by mixing HBA and HBD at a suitable temperature in two ways: (1) the constituent with the lower melting point is first melted, and the high melting point constituent is added to the resulting liquid and the mixture is melted together; (2) both components with high melting points are mixed and melted together.^119^ In addition to these two methods, researchers have adapted to using the other methods listed in Figure 6 and Table 4.

**Figure 6:**
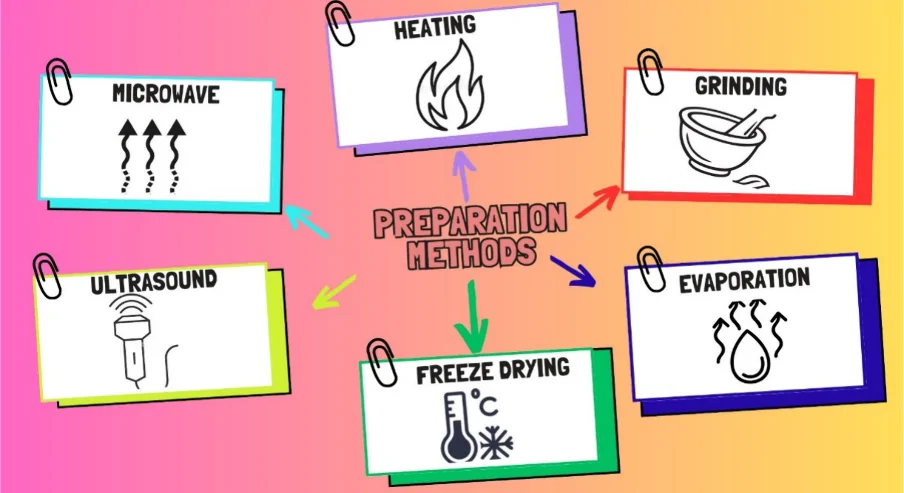
Preparation of DESs.

**Table 4:** Different Methods Have Been Used to Prepare Deep Eutectic Solvents

Method		Ref
(1). Heating	HBA and HBD are heated and stirred under an inert atmosphere until a homogeneous liquid forms.	^20^
(2). Grinding	Solid HBA and HBD are ground under a nitrogen atmosphere and/or inside a glovebox, using a pestle and mortar until a clear, homogeneous liquid is formed.	^120^
(3). Freeze-drying	Both the HBD and the HBA are dissolved separately in water. The two solutions are mixed, frozen, and subsequently freeze-dried to form a clear, homogeneous liquid.	^121^
(4). Evaporation	The constituents are dissolved in water, after which the water is evaporated at 323 K under vacuum or rotary evaporator, and the final mixture is further dried in a desiccator with silica gel until it reaches constant weight, signifying total loss of water.	^94^
(5). Microwave-assisted	It is mostly used in NADES preparation. The constituents are irradiated in a closed system at controlled power and temperature. The radiation interacts with the material, causing dipole rotation that results in a collision between the HBD & HBA constituents, causing dielectric heating that decreases the synthesis time.	^122^
(6). Ultrasound-assisted	When sound energy is applied, the sound waves cause cavitation. When the bubble reaches a critical size and implodes, heat and pressure are released, and that energy assists with the interactions between the HBD and HBA, resulting in the formation of NADES.	^123^

### 4.3 Properties of DESs

While DESs and ILs have different chemical properties, they share similar physical properties, such as designability, low vapor pressure, wide range of liquidity, and nonflammability.^89^ DESs present numerous advantages over conventional ILs, and their properties are illustrated in Figure 7. For example, depending on their composition, some DESs are less viscous than ILs, and they can also be acquired from inexpensive, toxicologically well-characterized renewable resources, and thus can be easily shipped for large-scale applications;^89^ ChCl is available as chicken feed, and urea is found in fertilizers.^124^ Choline, the most common HBA, is a vitamin B constituent and nutritional supplement for livestock. It is produced annually in a megaton, which reflects its wide range of availability.^89^ However, ChCl has regulatory and safety concerns. Marchel et al.^125^ studied the thermal stability of DESs at 80, 100, and 120 °C for 2 h using high-performance liquid chromatography (HPLC) to assess them and headspace gas chromatography–mass spectrometry (HS–GC–MS) to confirm the presence of volatile compounds. They reported that ChCl-based DESs exhibit thermal instability at moderate temperatures (≥80 °C), which can lead to chemical degradation and the formation of volatile byproducts.^125^ Upon heating, they begin to release volatile compound such as methylene chloride and methylamine, accompanied by discoloration and unpleasant odors.^125^ Among the studied systems, ChCl/malonic acid demonstrated the highest thermal sensitivity even at relatively low temperatures.^125^ This degradation also compromises the solvent’s effectiveness and recyclability in long-duration or high-temperature pretreatment processes. Although naturally occurring, industrial-scale ChCl is primarily produced from petrochemical-derived precursors such as ethylene oxide and trimethylamine,^89^ which alarms its sustainability credentials. Its safety profile restricts its use in sensitive applications such as cosmetics and food because it is a quaternary ammonium salts and a neurotransmitter. Moreover, its hygroscopic nature can affect storage and handling, physical properties, and reproducibility.^126^ For high-temperature applications, DES formulations could be improved either by incorporating stabilizers or cosolvents, or by exploring alternative HBAs with enhanced thermal stability.

**Figure 7:**
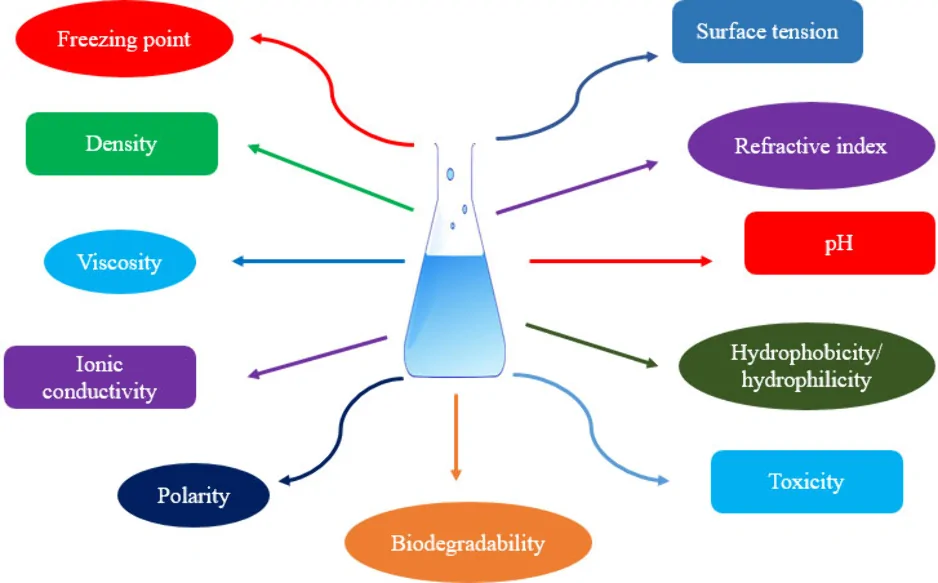
Properties of DESs.

DESs can be easily prepared by mixing the two components, without any further purification or waste generation steps, at mild temperature and atmospheric pressure.^124^ However, this simplicity applies to laboratory-scale under controlled conditions; in practice, reproducibility and scalability can be compromised by the hygroscopic and thermal instability of some DES components.^98,127^

DESs are also environmentally friendly because they do not require any organic solvents, have low toxicity, are nonflammable, have low volatility, and have tunable polarity, which merits DESs as potential substitutes for over 600 volatile organic compounds (VOCs), high biodegradability and biocompatibility.^128^ Moreover, because of their insensitivity to water, specifically hydrophobic DESs, they are incapable of activating enzymes during the conversion of biomass, which will be useful for undried biomass with high moisture content. However, these materials are generally less chemically inert.^89^ Although DESs present more benefits than traditional ionic liquids, their large-scale industrial implementation remains limited due to challenges such as viscosity, thermal instability, and difficulties in solvent recovery and recyclability. It is crucial to understand the physicochemical properties of DESs not only to optimize their laboratory performance but also to address these limitations in an industrial context.

#### 4.3.1 Freezing Point (T_f_)

DES is generally characterized by a lower freezing point compared to that of individual parent constituents,^129^ as depicted in Figure 4. The low depression of the freezing point results from the interaction between the halide anion and the hydrogen bond donor constituent.^130^ While many DESs exhibit a depressed freezing or melting point (Tm), it is important to note that some DESs lack a distinct melting transition but instead exhibit only a glass transition temperature (Tg), reflecting their amorphous or highly viscous nature.^20^ DESs with a freezing point lower than 50 °C are favorable because they can be employed as inexpensive and safe solvents in various fields. HBD has a great influence on the formation of DESs because of its stronger ability to form hydrogen bonds with HBA.^20^ For instance, using carboxylic acids or sugar-derived polyols as HBDs can yield room-temperature liquid DESs.^131^ Moreover, acids with the lowest molecular weight as HBDs showed the largest depression of the freezing point.^87^ Similarly, HBA can impact the freezing point, nature of organic salts (ammonium or phosphonium salts), and anions of the salts.^20^ For instance, in urea- and choline-based DESs, the freezing point decreases in the order F^–^ > NO_3_^–^ > Cl^–^ > BF_4_^–^, denoting a relationship with hydrogen bond strength.^20^ The organic salt/HBD molar ratio also has a significant effect on the DES freezing point.^132^ Overall, as proposed by Abbott et al.,^87^ the freezing point of HBD-salt eutectic mixtures is dependent on the (1) DES lattice energy, (2) anion-HBD interaction, and (3) entropy changes resulting from the formation of a liquid phase.

The thermal stability of DESs is a critical factor that influences their applicability in high-temperature processes. A common parameter used to evaluate thermal stability is the onset decomposition temperature (T_onset_), which is the temperature at which noticeable mass loss of DES begins, signaling its breakdown. A systematic investigation reported in *Acta Physico-Chimica Sinica* ^133^ examined 40 distinct DES systems using thermogravimetric analysis (TGA) to assess their T_onset_. Unlike ILs where one ionic species decomposes first at elevated temperature, followed by its ionic counterpart, DES decomposition occurs via the weakening of hydrogen bonds the HBA/HBD, releasing the constituents, which then volatilize.^133^ HBDs, due to their lower thermal stability, tend to degrade first while HBAs degrade at higher temperatures.^133^ T_onset_ is influenced by several factors. The strength of the hydrogen bonds is important as it enhances thermal resistance by stabilizing the eutectic structure and delaying component volatilization, therefore requiring greater energy to disrupt, leading to a higher T_onset_.^133^ The intrinsic thermal stability of HBDs also influences the T_onset_; the more stable it is, the higher the T_onset_.^133^ The anion of the HBA also affects thermal stability; due to its high electronegativity, Cl^–^ is more capable of forming hydrogen bond networks with HBDs compared to Br^–^ and I^–^; however, the resulting differences in ΔT_onset_ values are minimal.^133^ In addition, the BHA/HBD molar ratio affects the strength of the hydrogen bonding; optimized ratios can enhance thermal stability; changing ChCl/glycerol from 2:1 to 1:1 lowered the T_onset_ by ∼15–30 °C because relative to HBA molecules, HBD molecules are fewer, reducing the strength of the hydrogen bond network.^133^ Lastly, during thermogravimetric analysis, 5 °C min^–1^ is an ideal heating rate to study thermal behavior as a higher heating rate would lead to the temperature hysteric effect due to kinetic lag, potentially overestimating the long-term thermal stability of DES.^133^ However, slow heating rates may allow compositional changes before or during measurements such as partial evaporation or predecomposition.^134^ Additionally, temperature increases rapidly at a fixed rate and the sample passes through its critical decomposition point rapidly, and significant mass loss may not be detected.^125^ Liu et al.^134^ proposed the use of High-Resolution TGA (HR-TGA) as it provides better resolution to distinguish multiple steps of decomposition that may overlap and improve the reliability of derived kinetic parameters. Moreover, the team complemented the HR-TGA with mass spectrometry to track volatiles, which yields more accurate kinetic parameters.^134^ As such, both formulation and experimental conditions must be controlled when assessing the thermal stability of DESs. Table 5 presents the freezing temperatures of the selected DESs.

**Table 5:** Freezing Temperature of Selected DESs Adapted from Smith et al.^89^

DES	T_f_/°C	Ref
1:2 ChCl/urea	12.0	^87^
1:2 ChCl/thiourea	51.0	^135^
1:2 ChCl/1-methylurea	29.0	^20^
1:2 ChCl/1.3-dimethylurea	70.0	^20^
1:2 ChCl/1.1-dimethylurea	149.0	^20^
1:2 ChCl/acetamide	51.0	^20^
1:2 ChCl/benzamide	92.0	^20^
1:1 ChCl/adipic acid	85.0	^87^
1:1 ChCl/benzoic acid	95.0	^87^
1:1 ChCl/citric acid	69.0	^87^
1:1 ChCl/malonic acid	10.0	^87^
1:1 ChCl/oxalic acid	34.0	^87^
1:1 ChCl/phenylacetic acid	25.0	^87^
1:1 ChCl/phenylpropionic acid	20.0	^87^
1:1 ChCl/succinic acid	71.0	^87^
1:1 ChCl/tricarballylic acid	90.0	^87^
1:1 ChCl/MgCl_2_·6H_2_O	16.0	^136^
Methyltriphenylphosphonium bromide/glycerol	–4.0	^137^
Methyltriphenylphosphonium bromide/ethylene glycol	–49.3	^137^
Methyltriphenylphosphonium bromide/2,2,2-trifluoroacetamide	–69.3	^137^
Benzyltriphenylphosphonium chloride/glycerol	50.4	^137^
Benzyltriphenylphosphonium chloride/ethylene glycol	47.9	^137^
Benzyltriphenylphosphonium chloride/2,2,2-trifluoroacetamide	99.7	^137^
ZnCl_2_/urea	9.0	^101^
ZnCl_2_/acetamide	–16.0	^101^
ZnCl_2_/ethylene glycol	–30.0	^101^
ZnCl_2_/hexanediol	–23.0	^101^

#### 4.3.2 Viscosity

Viscosity is another important physical property of DES, as it influences the mass transport of the DES and therefore impacts their conductivities.^138^ Most DESs exhibit relatively high viscosities (>100 mPas) at room temperature.^130^ The high viscosity of DESs is due to the extensive hydrogen bond network across each constituent and the hydrogen bond network with other interactions such as van der Waals and electrostatic forces between HBA and HBD, which causes lower mobility of free species within the solvent system.^129^ For instance, a study by Abbott et al.^139^ revealed that the viscosity of 1:2 ChCl/glycerol significantly decreased upon addition of an organic salt due to the disruption of 3D intermolecular interactions in the glycerol network. This shows how HBA, HBD, and their interactions affect viscosity. The chemical nature of both HBA and HBD (ion size, type of salt, etc.) notably impacts the DES viscosity as well.^140^ For example, the viscosities of HBA/glycerol DESs decreased in the following order: benzyltrimethylammonium chloride > diethylethanolammonium chloride > choline chloride > triethylmethylammonium chloride,^141^ and the viscosities of choline chloride/HBD DESs decreased in the following order: zinc chloride > malonic acid > triethylene glycol > urea > levulinic acid > P-cresol > phenol > ethylene glycol.^142^ The use of derived carboxylic acids and sugars leads to high viscosities, owing to the presence of a more robust 3D intermolecular hydrogen-bond network.^143^ Another factor that influences DES viscosity is the HBA/HBD molar ratio. As shown in Table 6, the viscosities of 1:1 ChCl/fructose and 2:1 ChCl/fructose were 652.34 mPas^144^ and 11733.10 mPas,^145^ respectively. Temperature also affects DES viscosity. This is extensively proven by Sanchez et al.,^146^ using l-proline and betaine-based DESs. This can be explained by the hydrogen bond network between HBAs and HBDs, which decreases the DES viscosity with an increase in temperature.^142^ Moreover, Sanchez et al.^146^ also reported that incorporating water into a DES mixture significantly reduced viscosity due to the disruption of extensive hydrogen bonding within the DES (as seen in Table 6). The viscosity of DESs is also dependent on the free volume; therefore, hole theory can also be used to design low-viscosity DESs.^147^ Small cations or fluorinated hydrogen-bond donors were used to prepare low-viscosity DESs. Low viscosity DESs are considered valuable due to their potential applications as a green media.

**Table 6:** Viscosities of Selected DES at 25 °C Adapted from Omar and Sadeghi^142^ and Hansen et al.^129^

DES	η/mPas	Ref
1:2 ChCl/glycerol	302.0	^150^
1:2 Diethylethanolammonium chloride/glycerol	433.0	^141^
1:2 Benzyltrimethylammonium chloride/glycerol	716.6	^141^
1:2 Triethylmethylammonium chloride/glycerol	236.6	^141^
1:0.75 Tetrabutylammonium hydrogen sulfate/ninhydrin	48500.0 (at 50 °C)	^151^
1:0.75ChCl/ninhydrin	30500.0 (at 50 °C)	^151^
1:2 ChCl/urea	750.0	^152^
1:2 ChCl/ethylene glycol	44.4	^153^
1:2 ChCl/phenol	99.8	^154^
1:2 ChCl/malonic acid	1124.0	^152^
1:2 ChCl/ZnCl_2_	85000.0	^155^
1:2 ChCl/levulinic acid	216.0	^120^
1:2 ChCl/p-cresol	102.0	^154^
1:2 ChCl/triethylene glycol	839.0	^156^
1:3 ChCl/triethylene glycol	66.0	^157^
1:2 ChCl/phenol	99.8	^154^
1:3 ChCl/phenol	446.0	^158^
1:1 ChCl/fructose	652.3	^144^
2:1 ChCl/fructose	11733.1	^145^
1:2 ChCl/ethylene glycol	36.0 (at 20 °C)	^90^
1:2 ChCl/ethylene glycol	37.0	^152^
1:2 ChCl/glycerol	376.0 (at 50 °C)	^90^
1:2 ChCl/glycerol	259.0	^152^
3:7 Tetrabutylammonium bromide/imidazole	810.0	^159^
1:2 Tetrabutylammonium chloride/decanoic acid	429.0	^160^
1:3.5 ZnCl_2_/urea	11340.0	^101^
3:7 ChCl/imidazole	15.0 (at 70 °C)	^155^
1:3 ChCl/phenol	44.0	^158^
1:3 ChCl/o-cresol	77.0	^158^
1:1 ChCl/citric	9126.0	^87^
1:1 ChCl/malonic	1638.0	^87^
1:1 ChCl/oxalic	597.0	^87^
1:1 ChCl/succinic	1489.0	^87^
1:1 ChCl/acetic	162.0	^161^
1:1.6 ChCl/acrylic acid	115.0 (at 21 °C)	^162^
1:2 l-proline/_DL_-lactic acid	997.4 (at 50 °C)	^146^
1:2 l-proline/_DL_-lactic acid	246.1 (at 70 °C)	^146^
1:2 l-proline/_DL_-levulinic acid	219.6 (at 50 °C)	^146^
1:2 l-proline/_DL_-levulinic acid	73.4 (at 70 °C)	^146^
1:2 Betaine/_DL_-lactic acid	1266.0	^146^
1:2 Betaine/_DL_-lactic acid	190.2 (at 50 °C)	^146^
1:2 Betaine/_DL_-lactic acid	65.9 (at 70 °C)	^146^
1:5 Betaine/_DL_-lactic acid	245.3	^146^
1:5 Betaine/_DL_-lactic acid	50.6 (at 50 °C)	^146^
1:5 Betaine/_DL_-lactic acid	20.6 (at 70 °C)	^146^
1:2 Betaine/levulinic acid	1342.0	^146^
1:2 Betaine/levulinic acid	194.2 (at 50 °C)	^146^
1:2 Betaine/levulinic acid	67.0 (at 70 °C)	^146^
1:1:1 Betaine/_DL_-lactic acid/water	473.2	^146^
1:1:1 Betaine/_DL_-lactic acid/water	92.0 (at 50 °C)	^146^
1:1:1 Betaine/_DL_-lactic acid/water	37.9 (at 70 °C)	^146^
2:1:6 Betaine/citric acid/water	258.9 (at 50 °C)	^146^
2:1:6 Betaine/citric acid/water	90.9 (at 70 °C)	^146^

Viscosity measurement for DESs is time-consuming and there are many factors that affect it, inhibiting their applications. In their investigation of DES viscosities, Shi et al.^148^ employed and compared four machine learning algorithms to train viscosity predictive models: support vector regression (SVR), random forest (RF), neural network (NN), and extreme gradient boosting (XGBoost) using a data set of DES systems from reported literature, with 994 experimentally measured viscosities, while considering features such as basic properties (molecular mass of HBA and HBD, HBA/HBD molar ratio and temperature), molecular structure (encoded using Morgan fingerprints), and water content. Among these, XGBoost demonstrated the highest predictive accuracy, the best-performing model was XGBoost with all incorporated features, achieving a coefficient of determination (*R* ^2^) of 0.9861 and a mean squared error (MSE) of 0.0422, outperforming the other models in key metrics and this performance was attributed to its ability to effectively model complex, nonlinear relationships while maintaining resistance to overfitting when using data with diverse conditions due to its regularization.^148^ Given the inconsistencies and unreliability of traditional methods to determine feature importance,^149^ the team developed and integrated a powerful interpretation method; SHapley Additive explanation (SHAP) to interpret feature contributions and influence on DES viscosity. In doing so, Shi et al.^148^ could rapidly predict viscosity values for new DESs and gain mechanistic insight into how features influence viscosity behavior. For example, using SHAP, it was evident that the intrinsic physicochemical properties of HBDs have a higher effect on viscosity than those of HBAs. Lower content of HBD has a high SHAP value; however, 1:1 and 2:1 molar ratio could both have a positive effect on viscosity but different ranges, which can be explained by the molar mass of the HBD; a high molar mass HBD leads to high viscosity. However, low molecular mass HBDs such as amides can have high SHAP values due to their ability to form extensive hydrogen bonds, which lowers the movement of free ion species within the DES. Furthermore, the functional group of HBDs directly influences the hydrogen bond network. HBAs mostly influence the viscosity by their alkyl chain length and cation size, which restricts the mobility of ion species. The same effect was observed in HBDs when longer chain HBDs increased their molecular dimension, hindering their free movement. Lastly, high temperature and high-water content reduce viscosity,^148^ and although the study incorporated water content as an input feature in the XGBoost model, it did not explicitly report the SHAP analysis results regarding the effect of water on DES viscosity. This predictive modeling provides valuable insights into the structure–property relationships governing DES viscosity and offers a practical framework for the rational design of new, low-viscosity DESs.

#### 4.3.3 Polarity

Until recently, polarity was not usually considered when studying DESs. This is important because it reflects the overall solvation capacity of the solvents.^118^ Most DESs are polar, and their polarity is often estimated using solvatochromic parameters that consider the hypochromic (blue) shift or bathochromic (red) shift of UV–vis bands for negatively solvatochromic dyes such as Reichardt’s betaine dye or positively solvatochromic dyes such as Nile red, as a function of the solvent’s polarity.^163^ The betaine dye 30 is commonly used for measuring DES polarity,^142^ denoted at ET; the empirical scale of solvent polarity. However, it is unstable in ethaline, glyceline, maline, and reline based DESs so it can be replaced by Betaine dye 33, which is stable and the calculations are done the same as ET (30) for Betaine dye 30.^164^ In 2014, Pandey et al.^164^ conducted a study on the polarity of choline-based DESs using Betaine dye 30 and Betaine 33. 1:2 choline/glycerol has ET (30) of 57 and ET (33) of 66.4 kcal·mol^–1^. The ET (33) was also high (65.4 kcal·mol^–1^) for 1:2 ChCl/urea, while ET (30) was 58 kcal·mol^–1^. Moreover, both ET (30) and ET (33) of glycerol (57 kcal·mol^–1^ and 66.3 kcal·mol^–1^, respectively) increase upon the formation of DESs.

Another polarity scale is the multiparameter scale of Kamlet and Taft (hydrogen bond donating ability α, hydrogen bond accepting ability β, and dipolarity/polarizability π*).^165^ It is impossible to compare the polarity parameters obtained by different solvatochromic probes because polarity scales are probe-dependent and not universal.^166^ The polarity of the DESs was influenced by the interaction between HBA and the HBD of the DES. Zhang et al.^167^ reported the polarity of ChCl/glycerol in Betaine 30 with varying molar ratios. For 1:1, 1:1.5, 1:2, and 1:3 ChCl/glycerol systems, the ET (3) is 58.49 kcal·mol^–1^, 58.28 kcal·mol^–1^, 58.21 kcal·mol^–1^, and 57.96 kcal·mol^–1^, respectively. Florindo et al.^168^ reported that the chemical structure of the DES components significantly influences their polarity. ChCl-based DESs exhibited higher polarity due to the presence of a polar ionic component and hydroxyl functionality.^168^ In contrast, tetrabutylammonium chloride-based DESs showed markedly lower polarity, attributed to their bulky, hydrophobic alkyl chains that weaken dipole interactions.^168^ Nonionic HBAs such as menthol also resulted in low-polarity DESs, reflecting weaker hydrogen bonding and lower dipolar character.^168^ Similarly, variations in the HBD had a notable impact on polarity; stronger proton-donating HBDs such as levulinic acid and malonic acid led to higher polarity, while polyol-based HBDs also exhibit high polarity owing to their ability to form extensive hydrogen bonding networks.^168^ Furthermore, an increase in the alkyl chain length of the HBD decreases the polarity of the DESs.^168,169^ Therefore, for high-polarity solvent applications, a eutectic mixture consisting of ChCl and a small carboxylic acid HBD is particularly suitable.

Zhang et al.^170^ examined 56 DESs to evaluate how their Kamlet–Taft parameters influence polarity and dissolution performance in major lignocellulosic components (cellulose, lignin and xylan). Corroborating existing literature, the group reported that Kamlet–Taft parameters were significantly affected by the DES structure; however, while Kamlet–Taft parameters are helpful in guiding solvent selection, they do not guarantee performance, as other factors (molecular structure, physicochemical properties of DES and solutes) also significantly influence dissolution.^170^ Furthermore, Kamlet–Taft parameters and solubility varied with DESs and lignocellulosic components; DESs with β = 0.80–1.20 and β-α = 0.35–1.90 were able to dissolve cellulose, but those with β-α below 0.35 failed to dissolve cellulose well, highlighting the importance of balanced basicity for cellulose dissolution. Interestingly, the study discovered a strong linear correlation between lignin solubility and net acidity (α-β) and between xylan and β, while other Kamlet–Taft parameters did not show a strong correlation with the solubility of lignin and xylan. The relationship between DES components and DES polarity has been confirmed using a computational, predictive model; COSMO-RS,^171^ enabling accurate estimation of solvent polarity based on molecular structure and eliminating solvatochromic measurements. Results demonstrated strong agreement with experimental data, particularly for hydrophilic DESs.^171^ The polarity parameters of the selected DESs are presented in Table 7.

**Table 7:** Polarity Parameter of Selected DESs Adapted from Omar and Sadeghi^142^

DES	ET(30) kcal/mol	ET (33) kcal/mol	Ref
1:1 ChCl/glycerol	58.49	-	^167^
1:1.5 ChCl/glycerol	58.21	-	^167^
1:2 ChCl/glycerol	58.28	-	^167^
1:3 ChCl/glycerol	57.96	-	^167^
1:2 ChCl/glycerol	58.00	66.40	^164^
1:2 ChCl/glycerol	-	66.49	^168^
1:2 ChCl/urea	-	65.75	^168^
1:2 ChCl/urea	57.00	65.40	^164^
1:2 ChCl/ethylene glycol	57.30	65.70	^164^
1:2 ChCl/levulinic acid	-	50.26	^168^
1:2 ChCl/malonic acid	-	64.61	^168^
1:2 ChCl/glycolic acid	-	50.83	^168^
1:2 ChCl/ethylene glycol	-	65.91	^168^
Glycerol	57.00	66.30	^164^
1,2-Ethanediol	57.10	65.50	^164^
1:1 _DL_-menthol/levulinic acid	-	62.62	^168^
1:1 _DL_-menthol/octanoic acid	-	63.10	^168^
1:1 _DL_-menthol/dodecanoic acid	-	62.90	^168^
1:1 _DL_-menthol/acetic acid	-	62.45	^168^
1:2 tetrabutylammonium chloride/octanoic acid	-	61.52	^168^
1:2 tetrabutylammonium chloride/decanoic acid	-	60.23	^168^
1:2 tetrabutylammonium chloride/dodecanoic acid	-	61.57	^168^

#### 4.3.4 pH

The acidity or alkalinity of DESs is considered important as it merits the use of DESs in various industrial fields.^172,173^ For instance, the weak alkalinity of the ChCl/urea DES merits its use in CO_2_ absorption.^167,174^ DESs are composed of Brønsted acids and bases, so the acidity/alkalinity of HBA and HBD governs the DES pH.^142^ However, in pure and anhydrous DESs, it is difficult to quantify pH because standard pH relies on hydrogen ion activity in water,^175^ which is absent in these solvents. The pH values reported for DESs are often measured in aqueous-diluted systems or “apparent pH” ^176,177^ values from specialized methods such as spectrophotometric techniques with acid–base indicators,^178^ computational predictions based on donor–acceptor properties,^179^ or glass electrodes^178^ in the aqueous phase to estimate relative acidity. A few factors can shift the pH spectrum of DESs. A study by Hassan et al.^145^ discovered that increasing the molar ratio of HBD in ChCl/fructose DES systems shifts the pH of the DESs from acidic to basic, as can be seen in Table 8. Abbott et al.^178^ further proved that the addition of chloride ions to a ChCl/glycerol DES mixture reduced the DES acidity and shifted it to neutrality. Furthermore, the DES pH is also influenced by temperature. The pH of the DES decreases as the temperature increases, and it is also influenced by the hydrogen bond donor.^180^

**Table 8:** pH of Selected DESs

DES	pH	Ref
1:1 ChCl/fructose	6.10	^145^
1:1.5 ChCl/fructose	6.80	^145^
1:2 ChCl/fructose	6.90	^145^
1:2.5 ChCl/fructose	7.10	^145^
1:1 ChCl/citric acid	–0.30	^187^
1:1 ChCl/maleic acid	–0.30	^187^
1:2 ChCl/urea	10.22	^188^
1:2 ChCl/triethanolamine	10.66	^156^
1:5 Potassium carbonate/glycerol	13.24	^189^
1:5 Benzyltriphenylphosphonium Chloride/glycerol	7.06 (55 °C)	^190^
1:3 Benzyltriphenylphosphonium Chloride/ethylene glycol	5.74 (55 °C)	^190^
1:2 ChCl/glycerol	4.721	^180^
1:2 ChCl/ethylene glycol	4.66	^180^
1:1 ChCl/malic acid	2.08	^180^
2:1 ChCl/malic acid	2.45	^180^
1:10 ChCl/lactic acid	2.30	^180^
1:5 ChCl/lactic acid	2.25	^180^
1:1 ChCl/monohydrate citric acid	2.30	^180^
2:1 ChCl/monohydrate citric acid	1.58	^180^
9:1 Lactic acid/glycine	2.80	^180^
9:1 Lactic acid/alanine	2.68	^180^
1:1 ChCl/*p*-toluenesulfonic acid	4.60	^191^
1:1 ChCl/oxalic acid	1.74	^191^
1:2 ChCl/levulinic acid	4.42	^191^
1:1 ChCl/malonic acid	2.10	^191^
1:1 ChCl/malic acid	2.43	^191^
1:1 ChCl/citric acid	4.08	^191^
2:1 ChCl/tartaric acid	3.70	^191^

While mineral acids are traditionally used for their strong, proton-donating ability, acidic DESs have superseded them due to their catalytic behavior and sustainable nature. Dilute, inorganic and organic acid pretreatments^181^ are usually carried out at high temperatures (160–220 °C) with low substrate concentration (5–10 wt %) for effective hemicellulose hydrolysis.^181,182^ However, such conditions are energy-intensive (elevated temperatures) and can lead to the degradation of released monomeric sugars and soluble oligomers into fermentation inhibitors such as furfural and 5-hydroxymethylfurfural (HMF), which are strong inhibitors to microbial fermentation.^168,169,183^ These inhibitors necessitate additional detoxification steps, increasing the cost and complexity of downstream processing.^182^ For complete hydrolysis of both hemicellulose and cellulose, concentrated pure acids are used at a temperature ≤ 160 °C and a high biomass concentration (10–40% of substrate).^181^ Concentrated pure acids are noted for their extreme corrosiveness, which demands special reactor material (corrosion-resistant alloys/nonmetallic materials) to withstand the harsh chemical environment,^182^ which substantially inflates capital and operational costs. Additionally, their corrosive nature requires extensive neutralization post-treatment, which generates significant acid waste.^181^

In contrast, acidic DES pretreatments have a low operating temperature range, which facilitates their reduced energy consumption compared to acid pretreatments. At 60 °C, Kumar et al.^184^ achieved a high purity lignin of 60% (*w/w*) from rice straw using 1:5 ChCl/lactic acid. Additionally, the formation of inhibitors is relatively low. A comprehensive analysis was conducted on potential inhibitors (HMF, furfural, gallic acid, ferulic acid, and coumaric acid) generated during DES pretreatment by Procentese et al.,^185^ assessing their impact on subsequent enzymatic hydrolysis and sugar fermentation using NREL protocols. HMF and furfural concentrations remained low, under 0.015 g L^–1^ while gallic, ferulic, and coumaric acids were below detection thresholds (<0.1 g L^–1^), all lower than the typical threshold of enzymatic hydrolysis and fermentation,^184^ indicating that no additional detoxification step is necessary following DES pretreatment.^185^ Nevertheless, despite these promising results, the analysis could be strengthened by broadening the inhibitor screening to include phenolic aldehydes and organic acids under severe pretreatment conditions. Additionally, potential inhibitor accumulation should be evaluated following multiple cycles of solvent reuse. Moreover, acidic DESs are generally thermally stable and exhibit low volatility,^186^ which reduces the need for pressurized reactors, which are capital costly.

#### 4.3.5 Hydrophobicity and Hydrophilicity

The hydrophobicity of DESs has a very influential role in the field of biphasic systems and extraction.^142^ Most DESs are hydrophilic, while some are hydrophobic. However, the application of hydrophilic DESs is inhibited by their polarity and hydrophobic DESs are mostly used in most applications.^142^ Hydrophobic DESs are composed of hydrophobic constituents such as decanoic acid, 1-nonanol as HBDs, and quaternary ammonium salts and fatty acids.^103^ However, a recent study has shown that at all ratios of aqueous phases, quaternary ammonium salts remained stable.^111^ At equal phases ratio, tetrabutylammonium bromide/fatty acids-based DESs are destroyed due to the salt dissolution and dissociation in the aqueous phase, such solvents are termed quasi-hydrophobic.^111^ Moreover, tetraoctylammonium bromide-based DESs showed higher stability in the aqueous phase than tetrabutylammonium bromide-based DESs due to the steric hindrance between the larger hydrocarbon chains of tetraoctylammonium and water, suggesting that it is water-miscible and not hydrophobic.^111^ Hydrophobic DESs are characterized by low densities and moderate viscosities at room temperature,^142^ which enhance mass transfer during pretreatment. These effects are influenced by the chemical nature of both HBA and HBD; the large anion size results in high viscosity, and the hydrophobic long alkyl chain also results in lower hydrophobicity owing to the existing steric hindrance that inhibits the core salt charged with water.^142^ In hydrophobic DESs, long-chain carboxylic acids such as decanoic acids, as HBDs, are typically used in contrast to short-chain acids. For example, the density and viscosity of 1:2 tetrabutylammonium chloride/decanoic acid and tetraheptylammonium chloride/decanoic acid were 0.916 g/cm^3^, 265.26 η/mPas, and 0.890 g/cm^3^, 172.87 η/mPas,^103^ respectively, as shown in Table 9. Furthermore, the increase in temperature weakens the hydrogen bonding interactions within the DES, which contributes to enhanced hydrophobic behavior. This is often accompanied by a reduction in both density and viscosity.^142^ Their hydrophobic interactions and π–π stacking with lignin’s aromatic structures are good for selective delignification.^192^ Moreover, due to their inherent hydrophobicity, hydrophobic DESs maintain their integrity in water due to their intrinsic water immiscibility, which minimizes leaching of the DES components and facilitates efficient biphasic separation, enabling selective lignin partitioning into the DES-rich layer.^193^ However, their low polarity can inhibit effective biomass penetration.^193^ Additionally, challenges such as slow kinetics, high cost and unknown environmental profiles relating to longer-chain components restrict their broader application.^193^

**Table 9:** Hydrophobicity of Selected DESs was Adapted from Omar and Sadeghi^142^

DES	ρ/g cm^–3^	η/mPa s	Ref
1:2Tetrabutylammonium chloride/decanoic acid	0.916	265.260	^103^
1:2 Tetraheptylammonium chloride/decanoic acid	0.890	172.870	^103^
1:2 Tetraoctylammonium chloride/decanoic acid	0.888	472.580	^103^
1:8Tetraethylammonium iodide/1-Nonanol	0.857	14.760	^194^
1:8Tetrapropylammonium bromide/1-Nonanol (quasi-hydrophobic)^111^	0.894	136.820	^194^

### 4.4 Applications of DESs

Owing to their desirable properties, DESs have various potential applications. Many are still in their early developmental stages and are not exhausted. These applications are versatile, but ongoing research overlaps in many areas owing to the sharing of one desirable property.^129^ For instance, the solubility capability of DESs warrants their use in biomass processing and in pharmaceutical and medical research as drug delivery vehicles. DES is often recognized as a ‘greener’ alternative to ILs, so DESs can be applied in IL applications as a greener substitute.^195^ Figure 8 demonstrates versatility and research thrusts on the promising avenues that are being explored. However, most of these studies are still in the research and development stages. Using different HBAs and HBDs, the properties of DESs can be tailored for specific applications, and the results can be determined by the existing intermolecular relationship between HBA and HBD. Given the immeasurable number of HBA/HBD combinations, finding the most favorable DES mixture for any application would require exhaustive trial and error through millions of combinations.^129^ Computational approaches can significantly streamline this process using computational modeling and simulation techniques to explore a vast array of HBA/HBD combinations in silico.

**Figure 8:**
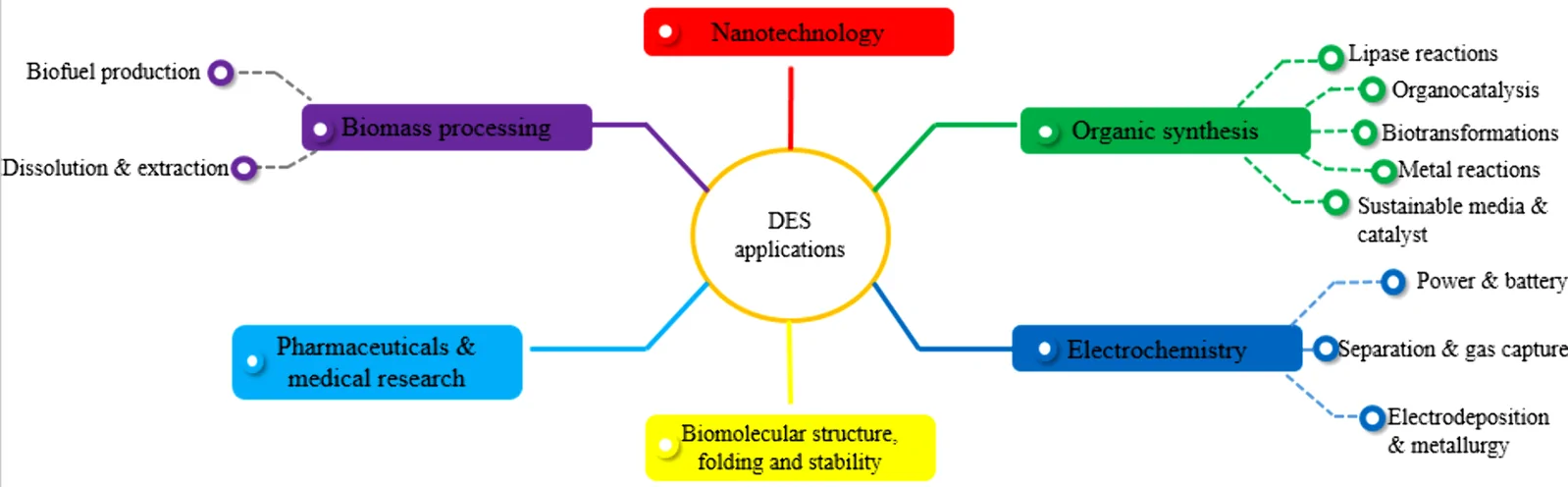
DESs applications.^89,129,167^

### 4.5 DES Pretreatment on Lignocellulose Biomass

In this section, we will discuss the application of DESs in biomass pretreatment. DESs are recognized as potential solvents for solubilization, extraction, and subsequent production of biobased products from lignocellulosic biomass. The search for novel DES mixtures effective for cellulose dissolution will continue to be an important research topic in the green industry.

#### 4.5.1 Biomass Fractionation

An essential aspect of lignocellulosic biomass pretreatment is the efficient disruption of the rigid biomass matrix. DESs have the ability to penetrate and partially or selectively dissolve the biomass component, therefore facilitating their fractionation while preserving the integrity of the polymeric network.^196^ Unlike ILs which exhibit superior cellulose-dissolving capabilities due to their highly ordered structure, DESs show significantly lower cellulose solubility.^197,198^ In ILs, upon contact with cellulose, the ordered structure is disrupted which leads to a notable entropy gain that facilitates cellulose dissolution. In contrast, DESs inherently have a less ordered conformation that limits this entropy gain, resulting in reduced efficiency in cellulose dissolution.^197,198^ Therefore, DES-mediated biomass fractionation primarily targets hemicellulose hydrolysis and lignin extraction through cleavage of key linkages within the LCC, including ether bonds between lignin units and ester/ether bonds between lignin and hemicellulose.^196^ Solid recovery yield serves as a practical and comparative indicator for evaluating the performance of various DES formulations across different biomass types and processing conditions. It refers to the proportion of biomass that remains undissolved after pretreatment.^199^ A high solid recovery percentage means less dissolved solids (lignin and hemicellulose) after DES pretreatment.^199^ Solid recovery is calculated by eq 1:

1 $$\mathrm{Solid recovery}\left(\mathrm{wt}\;\%\right)=\frac{\mathrm{Initial solid mass}-\mathrm{final solid mass}}{\mathrm{initial solid mass}}\times 100$$

There are several interrelated factors that influence the extent to which DESs can dissolve lignocellulosic biomass and understanding how these factors interact is crucial for optimizing both DES formulations and process conditions to enhance lignocellulose solubilization. One important factor is DES composition and type.^196^ For example, DESs with polyol-based HBDs are good for increasing enzymatic polysaccharide conversion due to their excellent enzyme stability. Hossain et al.^200^ investigated the pretreatment of rice straw using ChCl/polyol-based DESs and reported a low 66.10% solid recovery when using ChCl/ethylene glycol. These DESs possess abundant hydroxyl groups that contribute to their strong intermolecular hydrogen bonds which can affect their performance in lignocellulose fractionation but incorporating electron-withdrawing groups can improve xylan removal.^196^ Moreover, increasing the diol’s alkyl chain improves delignification due to increased hydrophobicity, which aligns with the lignin’s hydrophobic phenylpropanoid structure.^201^

Generally, the performance of polyol-based DESs is inferior to the acid-based HBDs owing to their lack of acidity, which lessens the removal of xylan and lignin.^202^ Moreover, the type of acid used is crucial in determining the effectiveness of the pretreatment process, as the acid’s functional groups are key to forming DESs capable of facilitating biomass separation.^196^ Moran-Aguilar et al.^203^ reported solid recovery of 37.15%, 44.80% and 22.93% from ChCl/acetic acid, ChCl/citric acid and ChCl/lactic acid pretreatments of sugar cane bagasse, respectively. This was attributed to −OH and −COOH groups of the HBDs that facilitated lignin dissolution; the hydroxyl groups in alpha hydroxyl acid DESs (citric and lactic acid) facilitate biomass fractionation due −OH group increasing the DES polarity, making it easier for hydrogen bonds to form with the biomass and two or more −COOH groups would inhibit it because a high number of hydrogen bonds and dimer chain lengths increases viscosity and reduces mass transfer between the biomass and DES.^196^ For instance, the solid recovery increased from tetramethylammonium chloride/lactic acid (50.41%) > tetraethylammonium chloride/lactic acid (52.64%) > tetrapropyl ammonium chloride/lactic acid (54.49%) > tetrabutyl ammonium chloride/lactic acid (58.48%) from the pretreatment of corn stover.^204^ Even though less extensively explored than HBDs, the HBA also influences the DES performance on fractionation. Lactic acid-based DESs with HBAs: glycine, betaine, and alanine were applied for the delignification of wheat straw. Among these, the alanine/lactic acid DES achieved a modest delignification of 24% after pretreatment at 60 °C for 24 h. In contrast, the other two showed no measurable delignification under the same conditions.^205^

Another important factor is the HBA/HBD molar ratio that not only influences the physicochemical properties of DES but also key performance metrics such as solid recovery and selective lignin removal and hemicellulose degradation.^196^ For example, the molar ratio of tetraethylammonium chloride/oxalic acid influenced the effectiveness of DES pretreatment of *Dendrocalamus sinicus*.^206^ As can be seen in Table 10, among the tested ratios, 2:1 exhibited the lowest solid recovery (62.51%), followed by 1:1.5 (63.16%), 1:2 (64.71%), and 1:1 (67.08%).^206^ Similarly, a lower solid recovery was also observed with a 3:1 glycolic acid/lactic acid DES (55.2%) compared to the 1:1 ratio (71.4%) and 3:1 glycolic acid/ChCl (48.5%) compared to the 1:1 ratio (72.6%) and this was attributed to an increase in glycolic acid content that enhanced the deconstruction of biomass in these DESs.^207^ Temperature is also important. Current research focuses on determining the optimal pretreatment temperature to enhance DES performance. While high temperatures generally facilitate delignification and xylan removal, they also increase carbohydrate degradation, so achieving a balance thermal setting for efficient biomass processing is important.^196^ For instance, with the ChCl/glycerol pretreatment of switchgrass, the solid recovery decreased from 56.65% < 49.41% < 48.44% for 110 °C, 120 °C, and 130 °C,^208^ respectively. Another significant factor is solid loading. The usual operating solid loading for DES pretreatment ranges from 5 to 10%,^209^ as higher solid loading has been shown to inhibit lignin and hemicellulose removal.^210^ However, DES pretreatment operating at high solid loading can improve the industrial feasibility of the process when integrated with other complementary technologies. Ai et al.^211^ used 30–50% solid loadings for ChCl/glycerol-mediated twin screw extrusion for the pretreatment of sorghum bagasse, achieving high sugar recovery but no significant change in solid recovery. Procentese et al.^185^ also observed no noticeable change in solid recovery when the solid loading was varied (1.25, 3.12, and 6.25 wt %).

**Table 10:** Solid Recovery of Different DESs Using Different Pretreatment Conditions

Biomass Type	DES	Pretreatment Conditions	Solid Recovery (%)	Ref
Rice straw	1:2 ChCl/ethylene glycol	150 °C, 15 h, 5 wt % biomass loading	66.10	^200^
	1:2 ChCl/glycerol	150 °C, 15 h, 5 wt % biomass loading	69.80	
	1:1 ChCl/xylitol	150 °C, 15 h, 5 wt % biomass loading	67.40	
	1:1 ChCl/sorbitol	150 °C, 15 h, 5 wt % biomass loading	67.60	
	1:1 ChCl/ethylene glycol	120 °C, 3 h, 5 wt % biomass loading	78.40	^201^
	1:1 ChCl/1,2-propanediol	120 °C, 3 h, 5 wt % biomass loading	76.10	
	1:1 ChCl/1,3-propanediol	120 °C, 3 h, 5 wt % biomass loading	76.10	
	1:1 ChCl/glycerol	120 °C, 6 h, 5 wt % biomass loading	79.50	
	1:1 ChCl/xylitol	120 °C, 3 h, 5 wt % biomass loading	81.10	
	1:1 ChCl/xylitol	120 °C, 24 h, 0.5 wt % biomass loading	∼5.10	^144^
	1:1 ChCl/fructose	120 °C, 24 h, 0.5 wt % biomass loading	∼6.50	
	1:1 acetylcholinium chloride/xylitol	120 °C, 24 h, 0.5 wt % biomass loading	∼4.40	
	1:1 acetylcholinium chloride/fructose	120 °C, 24 h, 0.5 wt % biomass loading	∼5.30	
	1:1 benzyldimethyl(2-hydroxyethyl)ammonium chloride/xylitol	120 °C, 24 h, 0.5 wt % biomass loading	∼2.00	
	1:1 benzyldimethyl(2-hydroxyethyl)ammonium chloride/fructose	120 °C, 24 h, 0.5 wt % biomass loading	∼4.80	
Corn stover	1:1 ChCl/lactic acid	130 °C, 2 h, 10 wt % biomass loading	48.83	^204^
	1:1 tetramethyl ammonium chloride/lactic acid	130 °C, 2 h, 10 wt % biomass loading	50.41	
	1:1 tetraethyl ammonium chloride/lactic acid	130 °C, 2 h, 10 wt % biomass loading	52.64	
	1:1 tetrapropyl ammonium chloride/lactic acid	130 °C, 2 h, 10 wt % biomass loading	54.49	
	1:1 tetrabutyl ammonium chloride/lactic acid	130 °C, 2 h, 10 wt % biomass loading	58.48	
	1:1 methyl triethyl ammonium chloride/lactic acid	130 °C, 2 h, 10 wt % biomass loading	50.53	
	1:1 benzyltrimethyl ammonium chloride/lactic acid	130 °C, 2 h, 10 wt % biomass loading	48.39	
	1:1 benzyltriethyl ammonium chloride/lactic acid	130 °C, 2 h, 10 wt % biomass loading	50.13	
	1:1 chlorocholine chloride/lactic acid	130 °C, 2 h, 10 wt % biomass loading	52.09	
	1:1 allyl trimethyl ammonium chloride/lactic acid	130 °C, 2 h, 10 wt % biomass loading	52.68	
	1:2 ChCl/ethylene glycol-sodium bicarbonate	80 °C, 3 h, 5 wt % biomass loading	87.54	^40^
	1:2 ChCl/ethylene glycol-sodium bicarbonate	100 °C, 3 h, 5 wt % biomass loading	∼86.00	
	1:2 ChCl/ethylene glycol-sodium bicarbonate	120 °C, 3 h, 5 wt % biomass loading	∼85.00	
	1:2 ChCl/ethylene glycol-sodium bicarbonate	140 °C, 3 h, 5 wt % biomass loading	80.49	
	1:2 ChCl/ethylene glycol-sodium bicarbonate	160 °C, 3 h, 5 wt % biomass loading	∼69.00	
	1:1 ammonium thiocyanate/urea	80 °C, 1 h, 20 wt % biomass loading	86.30	^214^
	1:1 ammonium thiocyanate/urea	80 °C, 1 h, 33 wt % biomass loading	85.60	
	1:1 ammonium thiocyanate/urea	100 °C, 1 h, 20 wt % biomass loading	83.10	
	1:1 ammonium thiocyanate/urea	100 °C, 1 h, 33 wt % biomass loading	82.00	
	1:1 ammonium thiocyanate/urea	80 °C, 1 h, 20 wt % biomass loading	82.80	
	1:1 ammonium thiocyanate/urea	80 °C, 1 h, 33 wt % biomass loading	83.00	
	1:1 ammonium thiocyanate/urea	100 °C, 1 h, 20 wt % biomass loading	84.30	
	1:1 glycolic acid/lactic acid	120 °C, 30 min, 5 wt % biomass loading	71.40	^207^
	3:1 glycolic acid/lactic acid	120 °C, 30 min, 5 wt % biomass loading	55.20	
	3:1 glycolic acid/ChCl	120 °C, 30 min, 5 wt % biomass loading	72.60	
	3:1 glycolic acid/ChCl	120 °C, 30 min, 5 wt % biomass loading	48.50	
Corn stalk	benzyl trimethyl ammonium chloride/glycolic acid/ethylene glycol	1:1:1	71.97	^215^
		1:1:2	65.67	
		1:1:3	66.90	
		1 h	75.63	
		2 h	72.60	
		3 h	65.67	
		4 h	64.44	
		100 °C	82.22	
		110 °C	74.48	
		120 °C	65.67	
		130 °C	64.06	
		140 °C	56.67	
		150 °C	50.53	
		160 °C	51.03	
Wheat straw	1:2:0.05 triethylbenzyl ammonium chloride/glycerol/aluminum chloride hexahydrate	100 °C, 1 h, 6.66 wt % biomass loading	55.09	^216^
		120 °C, 1 h, 6.66 wt % biomass loading	45.80	
		140 °C, 1 h, 6.66 wt % biomass loading	41.11	
*Dendrocalamus sinicus*	2:1 tetraethylammonium chloride/oxalic acid	120 °C, 7 h, 8.33 wt % biomass loading	62.51	^206^
	1:1 tetraethylammonium chloride/oxalic acid	120 °C, 7 h, 8.33 wt % biomass loading	67.08	
	1:1.5 tetraethylammonium chloride/oxalic acid	120 °C, 7 h, 8.33 wt % biomass loading	63.16	
	1:2 tetraethylammonium chloride/oxalic acid	120 °C, 7 h, 8.33 wt % biomass loading	64.71	
	1:1 tetraethylammonium chloride/oxalic acid	100 °C, 7 h, 8.33 wt % biomass loading	69.54	
	1:1 tetraethylammonium chloride/oxalic acid	110 °C, 7 h, 8.33 wt % biomass loading	68.09	
	1:1 tetraethylammonium chloride/oxalic acid	130 °C, 7 h, 8.33 wt % biomass loading	63.64	
	1:1 tetraethylammonium chloride/oxalic acid	120 °C, 5 h, 8.33 wt % biomass loading	61.96	
	1:1 tetraethylammonium chloride/oxalic acid	120 °C, 6 h, 8.33 wt % biomass loading	68.86	
	1:1 tetraethylammonium chloride/oxalic acid	120 °C, 8 h, 8.33 wt % biomass loading	63.74	
	1:1 ChCl/oxalic acid	130 °C, 6 h, 10 wt % biomass loading	71.55	
	1:1 ChCl/oxalic acid/1% ethylene glycol	130 °C, 6 h, 10 wt % biomass loading	65.05	^217^
	1:1 ChCl/oxalic acid/2% ethylene glycol	130 °C, 6 h, 10 wt % biomass loading	61.05	
	1:1 ChCl/oxalic acid/3% ethylene glycol	130 °C, 6 h, 10 wt % biomass loading	61.56	
	1:1 ChCl/oxalic acid/5% ethylene glycol%	130 °C, 6 h, 10 wt % biomass loading	61.54	
	1:1 ChCl/oxalic acid/ethylene glycol	110 °C, 6 h, 10 wt % biomass loading	65.30	
	1:1 ChCl/oxalic acid/ethylene glycol	120 °C, 6 h, 10 wt % biomass loading	62.04	
	1:1 ChCl/oxalic acid/ethylene glycol	130 °C, 6 h, 10 wt % biomass loading	61.05	
	1:1 ChCl/oxalic acid/ethylene glycol	140 °C, 6 h, 10 wt % biomass loading	60.08	
Sugar cane bagasse	1:4 ChCl/acetic acid	130 °C, 1.5 h, 6.66 wt % biomass loading	37.15	^203^
	1:1 ChCl/citric acid	130 °C, 1.5 h, 6.66 wt % biomass loading	44.80	
	1:4 ChCl/lactic acid	130 °C, 1.5 h, 6.66 wt % biomass loading	22.93	
Switchgrass	1:2 ChCl/glycerol	110 °C, 1 h, 10 wt % biomass loading	56.64	^208^
		120 °C, 1 h, 10 wt % biomass loading	49.41	
		130 °C, 1 h, 10 wt % biomass loading	48.44	
Grapevine	1:2 ChCl/urea	130 °C, 6 h, 10 wt % biomass loading	66.84	^218^
	1:5 K_2_CO_3_/ethylene glycol	130 °C, 6 h, 10 wt % biomass loading	56.35	
	1:4 ChCl/triethanolamine	130 °C, 6 h, 10 wt % biomass loading	71.63	
Oil palm empty fruit	1:2 ChCl/urea	120 °C, 8 h, 10 wt % biomass loading	93.56	^219^
	1:6 K_2_CO_3_/glycerol	120 °C, 8 h, 10 wt % biomass loading	74.80	
Aloe vera leaves rind	1:2 ChCl/glycerol	80 °C, 4 h, 10 wt % biomass loading	58.88	^220^

The choice of post-treatment washing method/solvent also influences the recovery of the solid residue. The high viscosity of DES leads to the solvent ‘sticking’ to the pretreated biomass surface which might cause low surface enzyme accessibility, so the type of solvents used to wash the DES-treated matrix is important. For example, Tenhunnen et al.^212^ used spectroscopic techniques to show that mild ethanol washing was ineffective in removing ChCl residual as the cationic groups of ChCl were electrostatically attached to the pulp’s anionic groups and reflected by the pulp’s nitrogen content. Sometimes, lignin redeposits onto the surface of the biomass after DES pretreatment, therefore increasing the solid yield.^213^ Yang et al.^213^ investigated five washing methods to remove surface lignin: distilled water, hot water, ethanol, sodium carbonate and sodium hydroxide. Distilled water and hot water washings had similar lignin removal of 48.01% while ethanol, sodium carbonate and sodium hydroxide had lignin removal of 85%, 82%, and 88%, respectively. Due to the lignin removal, the solid recovery also significantly decreased. The upcoming sections discuss how DES interacts with and alters lignocellulose components.

#### 4.5.2 Cellulose Degradation

A smart design of DES composition is important both for cellulose and DES, which are characterized by a strong hydrogen-bond network and their dissociation is not thermodynamically desirable.^221^ In terms of biomass dissolution, DESs are favorable compared to conventional organic solvents as they offer advantages such as stabilization of carbohydrates and furan derivatives through hydrogen-bond interactions, compatibility with enzymes, decrease in polyol viscosity, and inhibition of water reactivity to some extent.^222^ Crystallinity is an important parameter that plays a crucial role in the enzymatic hydrolysis of lignocellulosic biomass. Biomass crystallinity/crystallinity index (CrI) is a function of cellulose content in the biomass. Crystallinity is usually measured using techniques such as cross-polarization magic-angle spinning (CP/MAS) NMR,^223–225^ X-ray diffraction (XRD), Fourier transform infrared (FTIR), Raman spectroscopy, sum frequency generation (SFG), differential scanning calorimetry (DSC), and enzymatic cellulose binding molecules (CBMs).^225^ The crystalline structure of cellulose significantly influences the biomass recalcitrance, making the effect of pretreatment complex.^210^ Degradation of the amorphous regions leads to increased crystallinity, while destroying the crystalline regions further reduces cellulose crystallinity.^210^ DES pretreatment can also disrupt supramolecular structure on oriented crystalline cellulose chains in the microfibrils, therefore reducing the degree of polymerization of cellulose and increasing enzymatic accessibility.^226^ The lower the CrI, the higher the availability of substrate-binding sites, thus improving the biomass conversion yields. Moreover, while a lower CrI indicates biomass decrystallization, it does not conclusively imply cellulose has lost its crystalline structure. For instance, Procentese et al.^227^ studied the DES pretreatment of corn cob using 3 DESs (ChCl/urea, ChCl/glycerol, and ChCl/imidazole) at temperatures of 80 °C, 115, and 150 °C, as depicted in Table 11. The CrI for the untreated corn cob was 30.07 and upon ChCl/urea pretreatment, the CrI was 23.62 and 36.54 at 80 and 115 °C, respectively. The CrI was high because the removal of amorphous hemicellulose and lignin during pretreatment resulted in an increase in biomass crystallinity, whereas cellulose crystallinity was not appreciably lowered by DES. In essence, CrI is a relative measure reflecting the proportion of crystalline to amorphous material. After the removal of amorphous content, the relative amount of crystalline cellulose appears higher, even if the crystalline regions are unaltered. For ChCl/imidazole, the biomass CrI increases linearly with temperature; the CrI was 31.58, 40.08, and 49.22 at 80 °C, 115, and 150 °C, respectively. The crystallinity increases as the pretreatment temperature increases. The CrI increase does not necessarily reflect cellulose crystallinity, but the cellulose crystallinity fraction in the biomass sample. ChCl/urea at 80 °C was recognized as the optimal DES medium for biomass dissolution because it had the lowest CrI. The low CrI is likely due to changes in the cellulose fraction, and enhanced enzymatic digestibility is expected.^227^ However, a study by Nor et al.^228^ defied the temperature trend; they discovered that low crystallinity occurs at high-temperature pretreatment of oil palm when using 1:2 ChCl/urea at 110 °C (34.99) as compared to the untreated oil palm biomass (38.27) and ChCl/urea at 80 °C (39.43). Using a relatively low pretreatment temperature removes only the amorphous hemicellulose and lignin, while the cellulose remains intact and using a high temperature for the pretreatment results in the hydrolysis of para-crystalline cellulose. This shows that the removal of hemicellulose and lignin is crucial for biomass pretreatment. The subsequent disruption of cellulose depends on the temperature and chemical nature of the DES mixture. As can be attested by the CrI above, cellulose dissolution by DES pretreatment has proven difficult. Pan et al.^229^ conducted an investigative study of 1:2 ChCl/urea pretreatment of rice straw; the amorphous cellulose fraction was dissolved, but the α-cellulose did not dissolve. The group also reported that glucose yield did not consistently correlate with CrI,^229^ suggesting that CrI is not a reliable standalone indicator of cellulose digestibility and should be used in isolation to assess pretreatment effectiveness. Microcrystalline cellulose also showed no solubilization in 1:2 ChCl/urea^212^ and 1:2 ChCl/ZnCl_2_,^230^ ChCl and HBDs, oxalic acid, phenylacetic acid, malonic acid, glycerine, acetamide, and formamide.^900^ These reports indicate that the ChCl-based DESs are ineffective for cellulose dissolution. Unlike *N*-methylmorpholine N-oxide (NMMO) and ILs which have analogous physicochemical properties to DESs and have demonstrated significant cellulose solubility (up to 20 wt %^232^ and 45 wt %,^233^ respectively), DESs generally exhibit considerably lower cellulose dissolution capacity due to a combination of structural and thermodynamic reasons. In the case of NMMO, cellulose swelling and dissolution are driven by a substantial rearrangement of the hydrogen bond network within the system, enabling the disruption of the hydrogen bond network and the formation of new interactions between cellulose and NMMO.^234^ Similarly, ILs effectively dissolve cellulose through cooperative interactions involving both the anion and cation, which form hydrogen bonds with cellulose’s hydroxyl groups.^235^ Additionally, the IL’s high order contributes to a favorable entropic gain during dissolution further enhancing their solvating ability.^236^ In contrast, ChCl-based DESs have protic −OH groups capable of interacting with cellulose,^212^ however, the strong internal hydrogen bond network between the HBA and HBD restricts the availability of free interactions with cellulose, and the −OH groups in DES compete with cellulose to interact with Cl^–^ ions; lack of free binding sites reduces cellulose solubility in DESs.^237^ Moreover, DESs are not highly ordered which results in a smaller entropy gain upon cellulose dissolution, weakening the overall thermodynamic driving force.^236^ Nevertheless, DESs have shown potential in dissolving lower molecular weight carbohydrates. Hakkinen and Abbott^236^ systematically investigated the solubility of various carbohydrates, including glucose, sucrose, erythritol, cellobiose, starch and cellulose in ChCl-based DESs. Their findings indicated that DESs with lower viscosity and strong hydrogen bond formation enthalpy were effective at solvating mono- and disaccharides.^236^ The poor solubility of cellulose and starch was attributed to a high degree of polymerization and molecular weight.^236^ To improve cellulose solubility in ChCl-based DESs, Ren et al.^238^ activated cellulose using ultra-assisted saturated calcium chloride solution. Cellulose solubility was 1.43 and 2.48 wt % in ChCl/urea and ChCl/imidazole, respectively. The latter was improved to 4.57 wt % by adding 5 wt % polyethylene glycol (PEG). Malaeke et al.^239^ also observed a maximum of 6.10 wt % cellulose solubility in 1:1 ChCl/resorcinol assisted by ultrasound radiation. Generally, the mechanism of action is that the intermolecular hydrogen bonds in cellulose are broken down by the DESs to promote the formation of new hydrogen bonds between the – OH groups in cellulose and DES.^202^ HBA weakens the inter- and intramolecular hydrogen-bonding cellulose, which speaks to the hydrogen bond basicity (β) of DES.^86^ The hydrogen bond basicity (β) is affected by the presence of anions such as Cl^–^, HCOO^–^, OAc^–^, and (MeO)_2_PO_2_^–^, and others.^240^ CrI can be useful as a qualitative indicator of compositional change but as a predictor for digestibility, a more comprehensive evaluation including lignin and hemicellulose removal, sugar recovery and enzyme accessibility, is necessary to fully characterize the impact of DES pretreatment.

**Table 11:** Crystallinity Index (CrI) of Different Lignocellulosic Biomass after DES Pretreatment

Biomass	DES	Temperature (°C)	CrI	Ref
Corn cob	Untreated	-	30.07	^227^
Corn cob	ChCl/glycerol	80	31.20	^227^
Corn cob	ChCl/glycerol	115	29.79	^227^
Corn cob	ChCl/glycerol	150	44.81	^227^
Corn cob	ChCl/urea	80	23.62	^227^
Corn cob	ChCl/urea	115	36.54	^227^
Corn cob	ChCl/imidazole	80	31.58	^227^
Corn cob	ChCl/imidazole	115	40.08	^227^
Corn cob	ChCl/imidazole	150	49.22	^227^
Corn cob	Untreated	-	31.60	^241^
Corn cob	ChCl/lactic acid	90	38.60	^241^
Corn cob	ChCl/glycolic acid	90	30.60	^241^
Corn cob	ChCl/Levulinic acid	90	32.00	^241^
Corn cob	ChCl/malonic acid	90	29.50	^241^
Corn cob	ChCl/glutaric acid	90	30.80	^241^
Corn cob	ChCl/oxalic acid	90	31.60	^241^
Corn cob	ChCl/malic acid	90	31.70	^241^
Corn stover	untreated	-	31.10	^22^
Corn stover	ChCl/formic acid	130	57.20	^22^
Rice straw	untreated	-	37.90	^242^
Rice straw	Cholinium lysine	90	62.80	^242^
Rice straw	Cholinium glycine	90	65.40	^242^
Rice straw	Cholinium serine	90	68.90	^242^
Oil palm empty fruit bunch	Untreated	-	38.27	^228^
Oil palm empty fruit bunch	ChCl/urea (1 h)	80	39.23	^228^
Oil palm empty fruit bunch	ChCl/urea (4 h)	80	38.84	^228^
Oil palm empty fruit bunch	ChCl/urea (1 h)	110	34.99	^228^
Oil palm empty fruit bunch	ChCl/urea (4 h)	110	37.16	^228^
Date palm residues	untreated	-	27.44	^243^
Date palm residues	ChCl/glycerol	70	31.89	^243^
Date palm residues	Hydrothermal pretreatment followed by ChCl glycerol	70	33.57	^243^
Wheat straw	Untreated	-	41.20	^244^
Wheat straw	ChCl/acetamide	90	42.00	^244^
Wheat straw	ChCl/urea	90	43.50	^244^
Wheat straw	ChCl/glycerol	90	43.80	^244^
Wheat straw	ChCl/methylethanolamine	90	47.60	^244^
Wheat straw	ChCl/diethanolamine	90	50.30	^244^
Wheat straw	ChCl/monoethanolamine	90	53.90	^244^

#### 4.5.3 Hemicellulose Degradation

As previously discussed, hemicellulose is one of the polymers that contribute to biomass recalcitrance owing to the existing covalent and hydrogen bonding networks between the functional groups of lignin and hemicellulose.^245^ Therefore, pretreatment methods seek to cleave LCCs to enhance enzyme accessibility and hydrolysis yield.^124^ DESs hydrolyze the existing LCC linkages by disrupting bonding interactions and developing new and competing hydrogen bonds between the DES ion (i.e., chloride ion) and hydroxyl groups in the complex.^124^ Essentially, lignin is covalently linked to hemicellulose through benzyl ester, benzyl ether, and phenyl glycoside bonds, and further stabilized by strong hydrogen bonding.^246,247^ During DES pretreatment, these hydrogen-bond interactions are disrupted due to competitive hydrogen bonding between the Cl^–^ ions in the DES and the hydroxyl groups of lignin and carbohydrates, facilitating the separation of lignin from hemicellulose.^246^ The extent of hemicellulose removal varied according to the pretreatment conditions. Hemicellulose degrades relatively easily, especially when subjected to heat and acidic conditions, such as acid-based DESs (ADESs). In contrast, Hou et al.^248^ reported an approximately 20% hemicellulose hydrolysis yield after ChCl/urea pretreatment, which is a weakly basic DES, at 120 °C for 12 h, while Kumar et al.^184^ observed no hemicellulose hydrolysis after a 12 h ChCl/lactic acid and betaine/lactic acid (pH ∼ 2) pretreatment at 60 °C. However, Hou et al.^248^ reported 95.8% hemicellulose hydrolysis within 4 h at 120 °C with the strongly acidic DES ChCl/oxalic acid. Therefore, elevated temperatures ≥ 120 °C are required to degrade hemicellulose in strongly acidic DESs, whereas slight hemicellulose degradation was observed in mildly acidic DESs.

Enzyme preparation is also crucial, depending on pretreatment conditions and DES type; hemicellulose-rich enzyme preparations are important for xylan-rich pretreatment residues, and the same applies for lignin residues for pretreatment.^124^ For acid DES pretreatment, hemicellulose cannot be easily recovered, and consequently receives limited attention in research.^249^ Equal attention should be paid to the fate of hemicelluloses, as has been focused on cellulose and lignin, as solubilized hemicellulose can be valorized into many value-added products such as alcohols, carboxylic acids, and biopolymers.^245^

ADESs have attracted interest because of their excellent performance in biomass processing and enhancement of cellulose digestibility. There are Brønsted acid-based DESs (BADES) that can donate active protons to catalyze the intermolecular linkage cleavage in LCCs and release hemicelluloses from the lignocellulose matrix, which is then further hydrolyzed and solubilized in BADES.^248,250^ These DESs are composed of carboxylic acids, such as lactic acid, as HBDs. The performance of the lactic acid-based BADESs varied according to the paired HBA.^249^ Under the same conditions, ChCl/lactic acid and guanidine hydrochloride/lactic acid (GH/LA) removed 48% and 77.6% xylan from castor stalk, respectively. In another study, benzyltrimethylammonium chloride/lactic acid (BTMAC/LA) and benzyltriethylammonium chloride/lactic acid (BTEAC/LA) removed 80.8% and 66% of xylan, respectively, after 2 h of pretreatment at 140 °C.^251^ Another study by Liang et al.^204^ found similar xylan removal yields after the pretreatment of corn stover for 2 h at 130 °C. To enhance this effect, the ratio of Brønsted acids in the BADESs should be increased. Chloride ions in HBA can form new and competing hydrogen bonds with the hydroxyl groups of hemicellulose and lignin, thereby disrupting the hydrogen bond interactions in LCCs.^246^ Additionally, smaller HBAs have stronger electrostatic interactions between the nitrogen cation and the carboxyl group of lactic acid.^252^ Other BADESs containing HBDs, rather than lactic acid, have been used, and some present nearly 90% xylan removal efficiency. These include formic acid, acetic acid, glycolic acid, and levulinic acid.^249^

Lignin-based DESs have also been employed to synthesize BADESs for pretreatment and have shown promising results. Kim et al.^253^ pretreated switchgrass with ChCl/ *p*-coumaric acid (PCA) at 160 °C for 3 h, which led to a 70% xylan removal. Under the same pretreatment conditions, Wang et al.^254^ observed 82.3% hemicellulose removal from poplar biomass, and 90.8% and 88.9% glucose and xylan yields (after 72 h of enzymatic hydrolysis), respectively, using ChCl/p-hydroxybenzoic acid (PB). Ryu et al.^255^ investigated the effect of methoxy-based HBDs with ChCl: choline chloride/4-hydroxybenzaldehyde (ChCl/HBA), choline chloride/vanillin (ChCl/VA), and choline chloride/syringaldehyde (ChCl/SA) on DES properties and biomass pretreatment performance; ChCl/4-hydroxybenzaldehyde (HBA), which has no methoxy group, showed 83.3% and 50.1% glucose and xylan yields, respectively, after 72 h of enzymatic hydrolysis. Another type of acid-based DESs is Lewis’s acids (LADESs) which include metal chlorides such as AlCl_3_, CuCl_2_, and ZnCl_2_. These metal chlorides improve the hydrogen bond acceptor strength in acid-based DES and enhance the breakdown of hydrogen bonds in LCCs.^256^ They also act as active acidic site holders, which provide active protons to enhance the cleavage of linkages in LCCs and hemicellulose.^256^ A 1 h pretreatment of a ternary DES composed of ChCl/guaiacol/AlCl_3_ at 130 °C removed 100% hemicellulose from wheat straw, while ChCl/guaiacol alone was much less effective.^252^ Similarly, Huang et al.^257^ reported that a ternary guaiacol-based DES system not only achieved high delignification (∼79%) and excellent glucan retention (>90%) at 110 °C, but also exhibited strong recyclability with more than 90% of DES recovered and reused for the next cycle. Additionally, during the preceding cycling, the solid recovery progressively increased from 53.12% to 80.50% in the fourth cycle, denoting decreased pretreatment efficiency. The reduced pretreatment efficiency could be attributed to the consumption of AlCl_3_ during the process, which weakened both the acidity and hydrogen-bonding capability of the DES, and the accumulation of impurities from lignin and hemicellulose degradation, which contaminated and impaired the DES performance.^257^ The DES enabled efficient enzymatic conversion of glucan (96.2%) even after reuse. Another ternary DES of ChCl/Glycerol/FeCl_3_ produced a near 100% hemicellulose removal of *Pennisetum* at 120 °C for 6 h.^258^ Even though LADESs are just as effective as BADESs, they are not widely used because their synthesis uses metal chlorides, whose disposal may be problematic.^249^

Pretreatment conditions such as temperature, time, and solid loading affect the removal and degradation of hemicellulose in ADES pretreatment. Higher temperatures and prolonged pretreatment times are known to enhance hemicellulose removal efficiency.^249^ Lin et al.^259^ saw an increase in xylan removal from 47.7% to 68.7% upon temperature increase from 110 to 130 °C after ChCl/lactic acid pretreatment of bamboo biomass. A similar trend was observed for poplar biomass under the same pretreatment conditions and DES, as xylan removal increased by 35.3%. As extended times improve hemicellulose removal, severe pretreatment conditions may lead to increased xylan degradation. Lee et al.^260^ discovered that prolonging pretreatment time from 1 to 2 h actually doubles the furfural production during ChCl/oxalic acid (ChCl/OA) pretreatment of oil palm fronds. When Isci et al.^261^ conducted a microwave-assisted ChCl/formic acid (ChCl/FA) pretreatment of wheat straw, there was no furfural production after 50 s of microwave heating, but there was a marginal increase when prolonged to 120 s. A good way of reducing furfural production during pretreatment would be to achieve a higher solid loading.^249^ High solid loading increases viscosity and reduces mass transfer, which subsequently decreases xylan hydrolysis and its conversion into furfural. It also enhances efficiency owing to the handling of more biomass in a single reactor.^249^ Another way of reducing xylan conversion to furfural is to add water to the acid-based DES system to decrease the system acidity and therefore reduce xylose dehydration and furfural formation rate.^249^ Isci et al.^261^ added 5% water to ChCl/formic acid, and almost 68% furfural production was reduced. The same observation was made by Morais et al.^262^ upon the addition of 5% water to the ChCl/malic acid (ChCl/MA) and ChCl/glycolic acid (ChCl/GA) DES systems. Admittedly, the stronger the acid, the more efficient the hemicellulose removal. However, a higher ratio of acidic HBDs increases acidity and induces proton availability, which catalyzes the hydrolysis of xylan into xylose and xylose dehydration into furfural.^263^ Therefore, adjusting the HBA/HBD ratio in ADESs changed the fate of hemicellulose degradation. As the acidity of DESs plays a role in hemicellulose degradation, some studies have suggested the use of neutral or basic DESs to counteract the effect of acidity. However, neutral DESs are ineffective for biomass fractionation, and while basic DESs are effective for hemicellulose preservation, they are not often effective for biomass fractionation and require high pretreatment severity.^202,210^ The HBA type also influences hemicellulose transformation and degradation; choline in ChCl can form choline xyloside with xylose and hasten furfural formation.^264^ However, chloride ions are important for facilitating LCC cleavages and hemicellulose removal, so methyl triethylammonium chloride (MTAC), as an HBA, can serve as an alternative source of chloride ions.^204^ A deeper insight into the relationship between acid-based DESs properties and xylan degradation needs to be carried out to provide guidance for the future designs of acid-based DESs with good fractionation performance and xylan preservation. Alternatively, a DES biphasic system leverages DES to enhance the efficiency and furfural production through selective extraction in two distinct phases. Jinyuan et al.^265^ proposed a ChCl/*p*-toluene sulfonic acid monohydrate (PTSA)/γ-valerolactone (GVL) biphasic system to significantly enhance hemicellulose-furfural conversion from *Neosinocalamus affinis*, owing to the GVL that inhibits the condensation of hemicellulose degradation products, therefore yielding up to 68.6% furfural at mild conditions. Similarly, an aqueous ChCl/EG/acetone biphasic system pretreatment of switchgrass was proposed by Chen and Wan,^266^ which reported a 75% furfural yield, with more than 90% furfural selectively extracted into the acetone phase. Under optimized conditions, Sun et al.^267^ observed a furfural yield of 54.7% from *Eucalyptus*, following 100% hemicellulose degradation using the ChCl/LA/2-methyltetrahydrofuran biphasic system. The biphasic system is economically viable and produces high furfural yields. Additional research is required to optimize efficiency and explore potential improvements for large-scale implementations. The hemicellulose removal performance of ADES is presented in Table 12.

**Table 12:** Hemicellulose Removal Performance of ADESs

Biomass	DES	Pretreatment Conditions	Hemicellulose Removal	Ref
Corncob	1:2 BTMAC/lactic acid	140 °C, 2 h	66.0%	^268^
Corncob	1:2 BTEAC/lactic acid	140 °C, 2 h	80.8%	^268^
Corncob	1:1 Ethylamine hydrochloride/lactic acid	150 °C, 30 min	87.9%	^269^
Corn stover	1:2 ChCl/lactic acid	130 °C, 2 h	77.6%	^204^
Corn stover	1:2 ChCl/lactic acid	Microwave assisted	90.1%	^270^
		152 °C, 45 s		
Corn stover	1:2 BTMAC/lactic acid	130 °C, 2 h	73.9%	^204^
Corn stover	1:2 BTEAC/lactic acid	130 °C, 2 h	70.9%	^204^
Corn stover	1:7 BTEAC/lactic acid	140 °C, 1 h 30 min	90.1%	^271^
Corn stover	1:2 ChCl/formic acid	130 °C, 2 h	66.2%	^22^
Rice straw	1:5 ChCl/lactic acid	60 °C, 12 h	16.5%	^184^
Rice straw	1:5 ChCl/lactic acid	121 °C, 1 h	93.6%	^272^
Switchgrass	1:2 ChCl/lactic acid	130 °C, 30 min	80.4%	^273^
Switchgrass	1:2 ChCl/lactic acid	Microwave assisted	83.4%	^270^
		152 °C, 45 s		
Switchgrass	1:2 ChCl/PCA	160 °C, 3 h	70.7%	^253^
Switchgrass	50:97:3 GH/ethylene glycol/p-toluenesulforic acid	120 °C, 6 min	79.4%	^273^
Akebia herbal residues	1:2 ChCl/formic acid	120 °C, 8 h	87.0%	^274^
Akebia herbal residues	1:6 ChCl/acetic acid	120 °C, 8 h	55.4%	^274^
Akebia herbal residues	1:4 ChCl/glycolic acid	120 °C, 8 h	89.7%	^274^
Akebia herbal residues	1:4 ChCl/levulinic acid	120 °C, 8 h	47.2%	^274^
Wheat straw	1:9 BTEAC/lactic acid	100 °C, 12 h	78.4%	^275^
Wheat straw	1:2 ChCl/formic acid	Microwave assisted	90.0%	^261^
		120 s		
Wheat straw	25:50:1 ChCl/Gal/AlCl_3_	130 °C, 1 h	100.0%	^252^
Sugar cane	1:2 ChCl/lactic acid	120 °C, 3 h	90.0%	^276^
Sugar cane	1:2 ChCl/formic acid	110 °C, 2 h	95.6%	^277^
Poplar	1:2 ChCl/lactic acid	130 °C, 1 h 30 min	64.3%	^278^
Poplar	1:2 ChCl/PBA	160 °C, 3 h	82.3%	^254^
Bamboo	1:8 ChCl/lactic acid	130 °C, 1 h 30 min	77.1%	^259^
Bamboo	2:1:2 ChCl/ethylene glycol/p-toluenesulforic acid	100 °C, 10 h	93.4%	^269^
Castor stalk	1:2 ChCl/lactic acid	130 °C, 30 min	48.0%	^251^
Castor stalk	1:2 GA/lactic acid	130 °C, 30 min	77.6%	^251^
Eucalyptus	1:10 ChCl/lactic acid	110 °C, 6 h	94.3%	^279^
*Micanthus*	1:2 ChCl/formic acid	Microwave assisted	77.5%	^263^
		152 °C, 45 s		
*Micanthus*	ChCl/gly/succinic acid	120 °C, 3 h	58.5%	^268^
*Pinus bungeana* Zucc	1:10 ChCl/lactic acid	Microwave assisted	74.7%	^280^
		120 °C, 8 min		
*Abaca*	10:20:1 ChCl/ethylene glycol/AlCl_3_	110 °C, 30 min	94.8%	^276^
*Pennisetum*	62:124:1 ChCl/Gly/FeCl_3_	160 °C, 6 h	93.6%	^258^
Alkaline hydrogen peroxide-pretreated oil palm fronds	1:1 ChCl/CuCl_2_	100 °C, 4 h	100.0%	^281^
Reed straw	1:6 BTEAC/formic acid	130 °C, 3 h	60.0%	^282^

#### 4.5.4 Lignin Degradation

The initial step for the enzymatic breakdown of cellulose and lignin valorization is the efficient extraction of lignin from biomass, which increases cellulose accessibility. This process is termed delignification. Type III DESs are the most commonly used DES solvent systems for lignocellulosic biomass delignification.^21^ The delignification performance of DESs is influenced by the type of biomass (hardwood, softwood, and herbaceous grass) and treatment conditions (pretreatment time, temperature, particle size, pretreatment method, and DES: biomass ratio).^283^ As previously mentioned in Section 4.5.1, DESs can dissolve and depolymerize lignin by disrupting various aryl ether and ester bonds within it.^196^ The phenolic compounds obtained from depolymerization can therefore be valorized into functional materials.^196^ DES extracted lignin has structural changes; the molecular size is small with a narrow distribution, but it has a large number of phenolic hydroxyl functional groups. This was also supported by Provost et al.,^284^ when 78% of the extracted lignin exclusively possessed G-units, 61% of which contained β-O-4 interunit linkages, with a narrow molecular weight distribution. However, despite efficient depolymerization, the competing process of lignin recondensation or repolymerization can occur, which could explain the low overall yield of monomer products during DES pretreatment.^196^

Xu et al.^283^ did an extensive comprehensive variable analysis of parameters of choline-based DESs that influence the efficacy of delignification and besides the above-mentioned factors, DES conditions have a significant impact. For instance, the HBA: HBD molar ratio affects the strength of DES hydrogen bonds, which subsequently determines the mobility and biomass degradation ability of DES molecules. Using corncob, Zhang et al.^241^ discovered that lignin removed after ChCl/lactic acid DES pretreatment increased with an increase in molar ratio: 64.7%, 77.9%, 86.1%, 93.1% for 1:2, 1:5, 1:10, and 1:15 ChCl/lactic DES mixtures, respectively. Another factor is DES constituents; there is a number of chemicals that are suitable to be used as HBDs. The delignification yield varies according to the nature and number of functional groups in the HBDs.^21^ To contextualize the effect of the HBD nature, DESs with acidic HBDs are more effective for lignin and hemicellulose removal than polyol/alcohol HBDs and amide HBDs.^241,285^ This can be explained by the acidity coefficient or acid dissociation constant (p*K*_a_); DESs with a lower p*K*_a_ value are strongly acidic and therefore influence delignification.^178^ This is due to the presence of active protons in carboxylic groups, which promote the proton-catalyzed cleavage of different chemical linkages in lignin-carbohydrate complexes (LCC) and lignin.^254^ Their effects were independent of the HBA used. It is therefore important to evaluate the individual contributions of HBAs and HBDs to optimize lignin solubility. In some cases, the individual DES constituents demonstrate superior delignification efficiency compared to the DES itself; Sosa et al.^286^ showed that ChCl exhibited significant hydrotropic effects, markedly enhancing lignin solubility in aqueous solutions compared to ChCl/glycerol and ChCl/ethylene glycol. This is attributed to the ChCl’s intrinsic affinity for lignin dissolution and tendency to self-organize around lignin macromolecules in aqueous solution, therefore maintaining their solubility as DES components in aqueous solution, there is competition for these interactions.^286^

On the other hand, DESs with a hydroxyl HBD show poor results for biomass delignification due to an extensive hydrogen bond network that weakens the ability of HBA to compete with intramolecular bonding in lignin and increases the DES viscosity, which means additional energy is required to loosen the bonded structure to initiate interactions with lignin.^21,256^ With amine/amide HBDs, the pH is basic with moderate delignification performance.^219^ The stronger the basicity, the greater the delignification performance. This was ascribed to the deprotonation of the phenolic OH units of lignin in alkaline solutions. Zhao et al.^244^ reported that the delignification performance decreased from 82.0%, 73.5%, and 44.6% to 27.7% for ChCl:monoethanolamine (pH 10.9), ChCl:diethanolamine (pH 10.5), and ChCl:methyldiethanolamine (pH 10.4) to ChCl/urea (pH 8.2), respectively. Recently, phenyl- or lignin-derived compounds have been explored as HBDs and have shown great potential for the removal of lignin. These include vanillin, catechol, (PCA), p-hydroxybenzoic acid (PB), p-hydroxybenzaldehyde (PHA), p-hydroxybenzyl alcohol, gallic acid (GA), and guaiacol.^21^ They have been used in the pretreatment of various biomasses, such as poplar,^253^ hybrid poplar,^287^ *Arabidopsis thaliana*,^288^ *Cortex albiziae* herb residues,^289^ woody biomass,^254^ and wheat straw.^252^ Their delignification performance is due to the ability of phenolic compounds to form new strong π–π stacking interactions and H-bonds with lignin moieties, thus reducing or destroying the original interactions in LCC in lignocellulosic biomass.^246^ The active protons donated by carboxyl, phenolic OH groups, and phenyl groups facilitate proton-induced bond-cleavage in lignin.

To contextualize the effect of the number of functional groups in HBD, monocarboxylic acid-based HBDs exhibit optimal lignin removal capacity compared to di- and tricarboxylic acids.^219^ HBDs with shorter alkyl chain lengths are efficient in lignin fractionation because of electron donation from the alkyl group to the oxygen in the OH group, thereby intensifying the hydrogen bonding strength.^290^ The HBAs also play a crucial role in lignin fractionation. Halide anions in HBA tend to form hydrogen bonds with the hydroxyl groups in lignin because of their electronegativity, as shown in Figure 9, and they also contribute to the breakage of β–O–4 bonds and prevent lignin condensation.^291^ Furthermore, the coordinating ability of cations and functional groups also affects the performance of delignification.^292^ For instance, in the pretreatment of corncob using BTMAC/LA and BTEAC/LA in the temperature range of 100–140 °C, the lignin removal efficiency for BTMAC/LA is relatively higher at temperatures of 100 and 140 °C, owing to the shorter alkyl chain for BTMAC, which is less bulky than that for BTEAC, which is attributed to less steric hindrance during lignin removal.^268^

**Figure 9:**
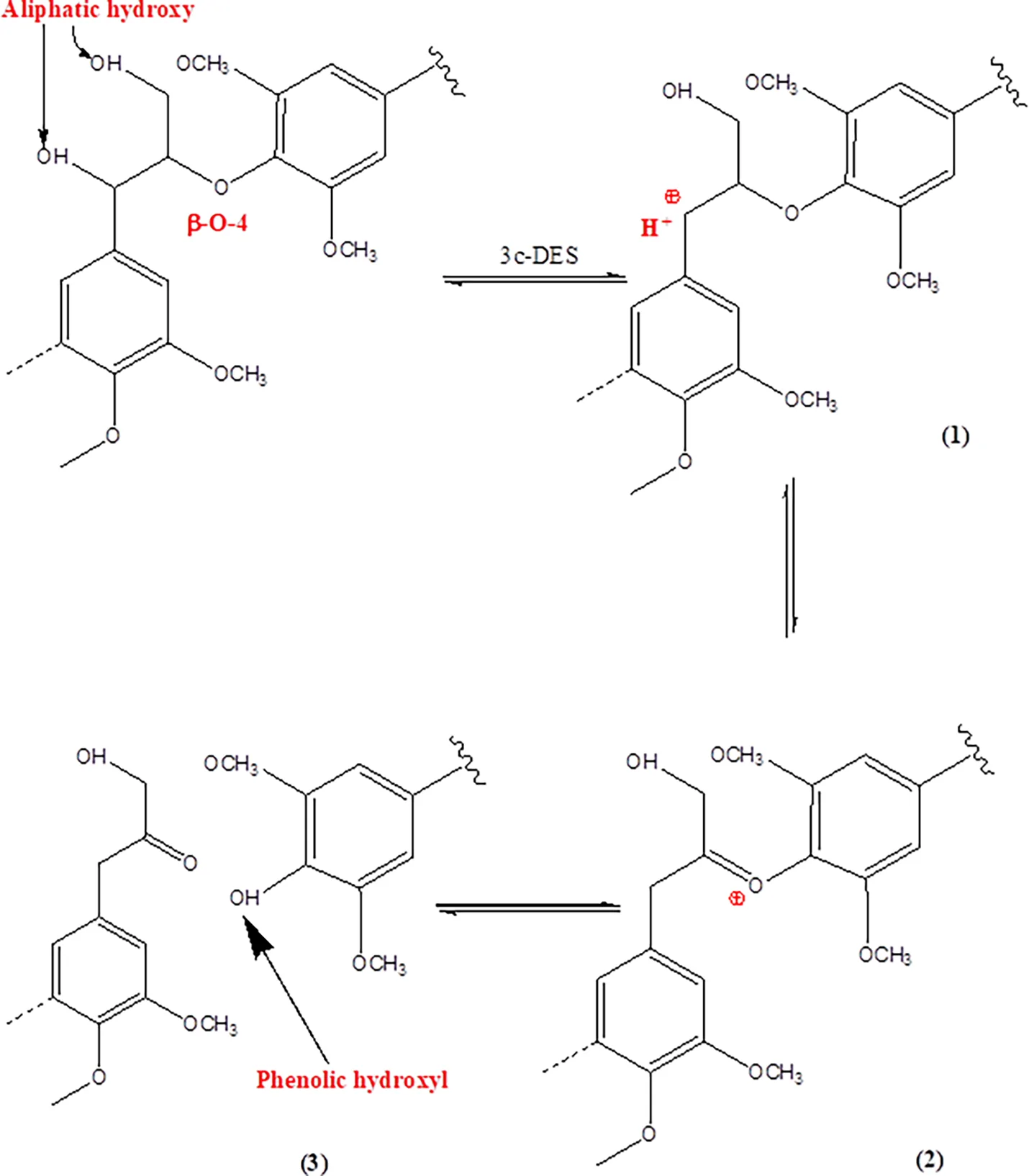
Schematic diagram showing the possible reaction pathway of the cleavage of the β–O–4 compound.^256^ Reproduced from ref 256 with permission from the Royal Society of Chemistry. Copyright 2018 The Royal Society of Chemistry.

Recently, researchers have explored ways to enhance DES lignin fractionation performance, either by additional constituents (catalyst or cosolvent) or by integration with other pretreatment technologies. In the pretreatment of hybrid *Pennisetum* energy grass using ChCl/glycerol, Wang et al.^293^ incorporated lewis acids to enhance high purity and antioxidative lignin yield. This enhancement is ascribed to the enhanced hydrogen-bonding acceptor strength and acidity of ChCl/glycerol. AlCl_3_·6H_2_O has also been used to markedly improve lignin fractionation based on an acidic multisite theory. Huang et al.^252^ also reported significant improvements in lignin degradation using a ChCl/guaiacol DES system combined with AlCl_3_. In the absence of AlCl_3_, the ChCl/guaiacol DES had a negligible effect on biomass deconstruction, with solid recovery remaining around 95% across the 80–130 °C pretreatment range. This indicated that ChCl/guaiacol alone was insufficient to compete with the internal hydrogen bonding network of wheat straw. However, with AlCl_3_ added, the pretreatment efficiency significantly increased, as shown by the decline in solid recovery (93.50% to 57.63%), with rising temperature. The DES system effectively degraded lignin and enhanced enzymatic digestibility.

In a study by Xia et al.,^256^ the lignin fractionation efficiency improved from 3.61% to 95.46%. The coordination of AlCl_3_·6H_2_O provides active protons and acidic sites that enhance the proton-catalyzed bond cleavage in lignin. The incorporation of small quantities of H_2_SO_4_ into ChCl/glycerol during the pretreatment of switchgrass also significantly improved lignin fractionation.^208^ Heteropoly acids, such as silicotungstic, phosphotungstic, and phosphomolybdic acids, have also been used.^294^ In addition to acids, other conventional solvents, such as water, can be used. While the addition of water to a DES system decreases DES viscosity and enhances diffusity,^21^ there is a report of an opposite trend where the addition of 20–40 wt % of water decreases the delignification performance of acidified ChCl/glycerol. This is ascribed to the fact that water competes for association with the Cl^–^ anion, thus disrupting the hydrogen bond network and forming a new one, thereby altering the supramolecular network of the DES and losing important eutectic properties.^21,295^ Using FTIR and ^1^H NMR, Dai et al.^296^ demonstrated that there is progressive weakening of hydrogen bonding interactions upon water addition to NADESs, with these interactions entirely disappearing at 50% (v/v) water addition, resulting in the loss of DES integrity and solvent functionality. Similarly, Zhang et al.^297^ reported that although modest water addition reduces viscosity, higher water content reduced delignification efficiency as water molecules compete with DES components for hydrogen bonding. The competition subsequently disrupts the supramolecular network, which diminishes its capacity to dissolve lignin. Therefore, the addition of water may be either harmful or beneficial, depending on the nature of DES constituents.

##### 4.5.4.1 Integral Methods with DES

To further enhance DES lignin removal, pretreatment methods such as ultrasound, microwave, hydrothermal, inorganic salt incorporation, and torrefaction can be used.^21^ The principles and determining factors of DES-integrated methods to enhance lignin removal are summarized in Table 13. Ultrasound irradiation enhances lignin fractionation via cavitation and mechanical effect.^21^ During ultrasonification, the mechano-acoustic effects cause microbubble cavitation, microjets shooting on the surface, and strong agitation, thereby increasing the micropores for a higher surface and contact with the DES, subsequently enhancing lignin fractionation.^21^ The factors determining the ultrasonification efficiency are power, frequency, and time, and they should be designed in a way that maximizes lignin fractionation while minimizing cost.^298^ Another promising method is a microwave-assisted DES strategy. This process saves time and energy and decreases the severity of DES pretreatments.^299^ Its efficiency owes to the electric dipole of DESs; microwave radiation maximizes the DES ionic character and increases their molecular polarity by interacting with polar molecules of DES, resulting in rapid, uniform volumetric heating through dipole rotation and ionic conduction with a lower energy loss.^300^ In addition to faster heating rates, microwave radiation also promotes selective bond cleavage during lignin depolymerization because lignin bonds such as C_β_–O in β–O–4 are stretched due to the application of an electric field during microwave irradiation.^301^ The factors to be considered during this method are the microwave penetration depth, DES properties, biomass type, and costs.^21^

**Table 13:** Principles and Determining Factors of DES-Integrated Methods to Enhance Lignin Removal

	Principle	Determining Factors
Ultrasound radiations	Uses high-frequency sound waves to generate microbubbles in the DES solution	Ultrasound power and frequency
		Treatment duration
Microwave-assistance	Microwave causes the polar DES molecules to oscillate, facilitating their ionic character and solvent penetration into the LCC matrix	Microwave power and frequency
		Heating time and temperature
		DES composition
		Biomass type
Surfactants	Reduces the DES surface tension, increases the surface area of the biomass, and emulsifies lignin from the DES system.	Surfactant type and concentration
		Stirring and mixing conditions
		Interaction with DES and its components
Hydrothermal pretreatment	Uses high temperature and pressure to weaken the LCC matrix and enhance lignin extraction	Temperature and pressure
		Reaction time
		DES/water ratio

Surfactants are also recognized as potential novel chemicals for enhancing biomass processing performance. Their amphiphatic nature has merited them an application as they can decrease the surface tension between two liquid phases, create structural aberrations, and increase the surface area of the biomass, enhancing its solubility, and mass transfer capacity.^302,303^ Moreover, surfactants prevent the nonproductive bonding of lignin to cellulolytic enzymes, which was further proved experimentally by Li et al.^217^ using Tween 80. The results showed 85.72% glucose, and 91.05% xylose yields in the enzymatic hydrolysis of an integrated system of Tween 80 and ChCl/oxalic acid/ethylene glycol. Ling et al.^277^ also incorporated Tween 80 during the ChCl/formic acid pretreatment of sugar cane bagasse and glucose production was improved while the hydrolysis time was reduced from 72 to 48 h. Hemicellulose and lignin removal rates were 95.6% and 72.6%, respectively. Further research on this topic needs to be conducted, as this application is still in its infancy. Hydrothermal pretreatment can also be used prior to DES pretreatment to deconstruct biomass cell walls and to cleave lignin into smaller fragments. Yang et al.^304^ developed an integrated system of AlCl_3_-catalyzed hydrothermal pretreatment followed by DES treatment for the delignification from poplar biomass and they achieved the delignification yield of 96.94%. This method is a well-established approach that is inexpensive and produces few inhibitors.^17^ It uses the heating effects of either liquid or water vapor, which selectively disrupts the hemicellulose structure to increase hydrolysis efficiency and partially removes lignin, which subsequently removes amorphous cellulose fibrils.^17,305^

Hydrothermal pretreatment alone can facilitate the Kraft pulping performance of some biomass feedstocks, but cannot enhance delignification.^304^ The sequential hydrothermal-ChCl/ethylene glycol/AlCl_3_ pretreatment studied by Ma et al.^276^ on poplar biomass showed promising results for the fractionation of hemicelluloses, such as xylooligosaccharides, monosaccharides, and xylan-type hemicelluloses. Consequently, the integration of hydrothermal pretreatment, known for its high hemicellulose removal efficiency, with deep eutectic solvents (DESs), which are highly effective in lignin extraction, represents a promising strategy for advancing biomass valorization. Wang et al.^306^ developed a synergistic hydrothermal-DES ChCl/lactic acid pretreatment method to simultaneously fractionate hemicelluloses and lignin from moso bamboo. There was a high hemicellulose yield of 98.2 and 80.1 wt % of lignin yield. The microwave/DES and ultrasound/DES pretreatment methods are recognized as great enhancers for delignification, but not for the total disruption of the whole biomass. On the other hand, hydrothermal/DES technology separates cellulose, hemicellulose, and lignin from the biomass, resulting in maximum product yields. In addition to these methods, DESs can also be integrated with other pretreatment strategies such as inorganic salts; a two-stage pretreatment with dilute NaOH before or after DES treatment results in high lignin fractionation. ChCl/urea pretreatment of sorghum straw had lignin removal rates of 49.0%, 79.3%, and 78.4% before and after the incorporation of dilute NaOH.^307^ All these reports have demonstrated great potential for enhancing the performance of DESs in the pretreatment of lignocellulose and may function at lower solid-to-liquid ratios, thus lowering the reaction mixture viscosity. Table 14 summarizes the delignification performance of various DESs on biomass.

**Table 14:** Delignification Performance of Various DESs on Biomass

Biomass	DES Mixture	Conditions	Lignin (%)	Ref
Corn cob	1:2 ChCl/imidazole	115 °C, 15 h	70.0	^227^
Corn cob	1:2 ChCl/imidazole	150 °C, 15 h	88.0	^227^
Corn cob	1:2 ChCl/urea	115 °C, 15 h	24.8	^227^
Corn cob	7:3 ChCl/glycerol	115 °C, 15 h	4.4.0	^241^
Corn cob	1:2 ChCl/lactic acid	90 °C, 24 h	64.7	^241^
Corn cob	1:5 ChCl/lactic acid	90 °C, 24 h	77.9	^241^
Corn cob	1:10 ChCl/lactic acid	90 °C, 24 h	86.1	^241^
Corn cob	1:15 ChCl/lactic acid	90 °C, 24 h	93.1	^241^
Corn cob	1:2 ChCl/glycolic acid	90 °C, 24 h	56.4	^241^
Corn cob	1:2 ChCl/levulinic acid	90 °C, 24 h	43.0	^241^
Corn cob	1:1 ChCl/malonic acid	90 °C, 24 h	56.5	^241^
Corn cob	1:1 ChCl/glutaric acid	90 °C, 24 h	34.3	^241^
Corn cob	1:1 ChCl/oxalic acid	90 °C, 24 h	98.5	^241^
Corn cob	1:1 ChCl/malic acid	90 °C, 24 h	22.4	^241^
Corn cob	1:1 ChCl/ethylene glycol	90 °C, 24 h	87.6	^241^
Corn cob	1:1 ChCl/glycerol	90 °C, 24 h	71.3	^241^
Corn cob	1:2 BTMAC/lactic acid	140 °C, 2 h	63.4	^268^
Corn cob	1:2 BTEAC/lactic acid	140 °C, 2 h	56.6	^268^
Corn straw	1:2 ChCl/lactic acid	120 °C, 6 h	51.8	^310^
Corn straw	1:2 Betaine/lactic acid	120 °C, 6 h	35.7	^310^
Corn stover	1:2 ChCl/lactic acid	Microwave, 4 s	79.6	^208^
Corn stover	1:2 ChCl/formic acid	130 °C, 2 h	23.8	^22^
Corn stover	1:2 ChCl/glycerol	180 °C, 2 h	∼60.0	^311^
Wheat Straw	1:9 ChCl/lactic acid	60 °C, 24 h	14.6	^312^
Wheat Straw	1:10 ChCl/lactic acid	60 °C, 24 h	29.1	^312^
Wheat Straw	1:1 ChCl/oxalic acid.2H_2_O	60 °C, 24 h	57.9	^312^
Wheat Straw	1:1 ChCl/malic acid	80 °C, 24 h	21.6	^312^
Wheat Straw	1:1 ChCl/malonic acid	60 °C, 24 h	3.8	^312^
Wheat Straw	1:2 ChCl/urea	80 °C, 24 h	1.3	^312^
Wheat Straw	1:2 ChCl/lactic acid	120 °C,12 h	78.6	^313^
Wheat Straw	1:9 TEBAC/lactic acid	100 °C, 10 h	79.7	^275^
Wheat Straw	1:2 ChCl/glycerol	90 °C, 12 h	24.7	^244^
Wheat Straw	1:6 ChCl/monoethanolanine	90 °C, 12 h	81.0	^244^
Wheat Straw	1:8 ChCl/diethanolamine	90 °C, 12 h	73.5	^244^
Wheat Straw	1:10 ChCl/methyldiethanolamine	90 °C, 12 h	44.6	^244^
Wheat Straw	1:2 ChCl/acetamide	90 °C, 12 h	3.4	^244^
Wheat Straw	1:2 ChCl/urea	120 °C, 8 h	27.7	^244^
Wheat Straw	ChCl/glycerol/lactic acid	120 °C, 0.5 h	45.0	^299^
Rice straw	1:2 ChCl/lactic acid	60 °C, 12 h	51.0	^184^
Rice straw	1:5 ChCl/lactic acid	60 °C, 12 h	60.0	^184^
Rice straw	1:9 ChCl/lactic acid	60 °C, 12 h	59.0	^184^
Rice straw	1:2 ChCl/urea	130 °C, 8 h	44.7	^229^
Rice straw	1:2 ChCl/oxalic acid	120 °C, 1 h	75.9	^314^
Rice straw	1:1 ChCl/malic acid	80 °C, 6 h	51.3	^314^
Rice straw	1:2 Betaine/lactic acid	60 °C, 12 h	52.0	^184^
Rice straw	3:1 Proline/malic acid	120 °C, 12 h	48.7	^248^
Rice straw	1:1 ChCl/ethylene glycol	120 °C, 6 h	26.2	^201^
Rice straw	1:1 ChCl/1.2-propanediol	80 °C, 6 h	26.5	^201^
Rice straw	1:1 ChCl/1.3-propanediol	80 °C, 6 h	29.2	^201^
Rice straw	1:1 ChCl/formamide	120 °C, 6 h	45.8	^201^
Switchgrass	1:1 ChCl/PHA	160 °C, 3 h	0.4	^253^
Switchgrass	1:1 ChCl/catetol	160 °C, 3 h	49.0	^253^
Switchgrass	1:2 ChCl/vanilin	160 °C, 3 h	52.5	^253^
Switchgrass	1:1 ChCl/PCA	160 °C, 3 h	60.8	^253^
Switchgrass	1:2 ChCl/LA	Microwave, 4 s	72.0	^208^
Switchgrass	1:2 ChCl/glycerol	110 °C, 1 h	76.6	^208^
Switchgrass	1:2 ChCl/ethylene glycol	130 °C, 30 min	78.9	^263^
Switchgrass	1:1.94:0.06 GH/EG/PTSA	120 °C, 6 min	81.0	^273^
Switchgrass	1:2 + 1% + 10% ChCl/EG + H_2_SO_4_+water	130 °C, 30 min 20% solid loading	57.6	^263^
Switchgrass	1:2 + 1% + 10% ChCl/ethylene glycol + H_2_SO_4_+water	130 °C, 30 min 27% solid loading	51.8	^263^
Switchgrass	1:2 + 0.9% ChCl/glycerol + H_2_SO_4_	120 °C, 1 h	63.8	^208^
Switchgrass	1:2 + 1% + 20% ChCl/ethylene glycol + H_2_SO_4_+water	130 °C, 30 min	80.0	^291^
Poplar	1:1 ChCl/oxalic acid	Microwave, 3 min	80.0	^246^
Poplar	1:2 ChCl/lactic acid	145 °C, 6 h	78.5	^315^
Poplar	1:9 ChCl/lactic acid	120 °C, 6 h	98.1	
Poplar	1:2:0.28 ChCl/glycerol/AlCl_3_·6H_2_O	120 °C, 4 h	95.5	^256^
Poplar	1:2 ChCl/lactic acid	110 °C, 1.5 h	31.1	^304^
Poplar	1:2 ChCl/lactic acid	130 °C, 1.5 h	66.4	^304^
Poplar	1:10 ChCl/lactic acid	110 °C, 1.5 h	51.5	^304^
Poplar	1:10 ChCl/lactic acid	130 °C, 1.5 h	89.3	^304^
Poplar	1:1 ChCl/PCA	160 °C, 3 h	15.5	^254^
Poplar	3:2 ChCl/PBA	160 °C, 3 h	69.0	^254^
Poplar	1:2 ChCl/PHA	160 °C, 3 h	30.7	^254^
Poplar	2:1 ChCl/GA	160 °C, 3 h	50.0	^254^
Oil palm empty fruit bunch	1:5 ChCl/lactic acid	120 °C, 8 h	88.0	^219^
Oil palm empty fruit bunch	1:15 ChCl/lactic acid	120 °C, 8 h	61.0	^292^
Oil palm empty fruit bunch	1:2 ChCl/formic acid	120 °C, 8 h	50.0	^292^
Oil palm empty fruit bunch	1:1 ChCl/citric acid	120 °C, 8 h	20.6	^292^
Oil palm empty fruit bunch	1:2 ChCl/propionic acid	120 °C, 8 h	20.4	^292^
Oil palm empty fruit bunch	1:1 ChCl/succinic acid	120 °C, 8 h	10.7	^292^
Oil palm empty fruit bunch	1:1 ChCl/glucose	120 °C, 8 h	17.0	^219^
Oil palm empty fruit bunch	1:2 ChCl/urea	120 °C, 8 h	34.0	^219^
Oil palm empty fruit bunch	1:6 ChCl/glycerol	120 °C, 8 h	51.0	^219^
Oil palm frond	ChCl/CuCl_2_·2H_2_O	90 °C, 3 h+alkaline	55.4	^281^
		Hydrogen peroxide		
Douglas fir	1:2 ChCl/lactic acid	145 °C, 6 h	58.2	^315^
*Miscanthus*	1:2 ChCl/lactic acid	Microwave, 45 s	65.2	^208^
Eucalyptus	1:10 ChCl/lactic acid	110 °C, 6 h	80.0	^279^
*Eucalyptus camaldulensis*	1:10 ChCl/lactic acid	130 °C, 6 h	93.2	^279^
*Eucalyptus globulus*	1:2 + 25% Propionic acid:Urea + PTSA	190 °C, 8 h	80.04	^316^
Castor seed coats	1:2 ChCl/lactic acid	160 °C, 8 h	34.0	^317^
Castor seed coats	1:2 ChCl/acetic acid	160 °C, 8 h	31.0	^317^
Castor seed coats	1:2 ChCl/formic acid	160 °C, 8 h	33.0	^317^
Castor seed coats	1:2 ChCl/malonic acid	160 °C, 8 h	3.0	^317^
Castor seed coats	1:2 ChCl/glycerol	160 °C, 8 h	4.0	^317^
Castor seed coats	1:2 ChCl/ethylene glycol	160 °C, 8 h	8.0	^317^
Castor seed coats	1:2 ChCl/oxalic acid	160 °C, 8 h	9.0	^317^
Sorghum	1:2 ChCl/ethylene glycol	145 °C, 6 h	78.0	^318^
Willow	1:10 ChCl/lactic acid	120 °C, 6 h	91.8	^319^
Walnut endocarp cells	1:2 ChCl/lactic acid	145 °C, 6 h	64.3	^320^
Peach endocarp cells	1:2 ChCl/lactic acid	145 °C, 6 h	70.2	^320^
*Cunninghamia lanceolata*	1:10 ChCl/lactic acid	120 °C, 12 h	81.7	^307^
Pine	1:2 BTMAC/formic acid	150 °C, 2 h	80.6	^321^
*Arabidopsis thaliana* mutant	1:2 ChCl/vanillin	80 °C, 3 h	24.6	^322^
*Arabidopsis thaliana* mutant	1:2 ChCl/PHA	80 °C, 3 h	32.4	^322^
*Akebia*’ herbal residues	1:4 ChCl/formic acid	120 °C, 8 h	58.4	^274^
*Akebia*’ herbal residues	1:2 ChCl/lactic acid	120 °C, 8 h	40.7	^274^
Sugar cane bagasse	1:5 ChCl/formic acid	80 °C, 12 h	50.6	^323^
*Pinus pinaster* Ait.	1:1:4 ChCl/tartaric acid/LA	150 °C, 2 h	95.0	^324^
*Cortex albiziae*	1:1 ChCl/PCA	160 °C, 7 h	30.8	^289^
*Cortex albiziae*	1:1 ChCl/PCA	180 °C, 7 h	64.0	^289^

##### 4.5.4.2 Mechanism of Lignin Fractionation

Despite numerous studies on lignin fractionation from lignocellulosic biomass using DESs as the solvent media, only a few mechanisms have been proposed. Xia et al.^256^ postulated that for ChCl-based DESs, there is a possible interaction between Cl^–^ anion and the polysaccharide in lignocellulose. The Cl^–^ tends to form strong hydrogen bonds with hemicellulose hydroxyl groups due to its electronegativity, therefore enabling fractionation of the LCC. The resulting hydrogen bond strength was greater than that of the OH–O hydrogen bond in lignin. This indicates that the multisite nature of Cl^–^ is necessary to effectively compete with lignin and disrupt the hydrogen-bond network in lignocellulose. Additionally, the weak C–O bond strength in lignin makes it more prone to cleavage than the stronger C–C bond.^256^ As shown by the mechanism in Figure 9, acidolysis occurred in 3c-DES, leading to the formation of a positive proton at the Cα position (1). A resonance-stabilized positive charge then develops after the deprotonation and protonation steps, as shown in (2). Ultimately, after the hydration and deprotonation of Cβ, the ether bond is broken, resulting in the formation of ketone and phenol groups, as can be seen in (3).^256^

Furthermore, the coordinating role of cations in DES also affects the efficiency of lignin removal, the longer the alkyl chain, the lower the lignin removal yield because of the reduced interactions between the charged centers of the cations and anions. In addition, symmetric cations show better lignin removal performance than asymmetric cations because symmetric cations possess a larger intermolecular potential than asymmetric cations. Nevertheless, while Cl^–^ facilitates lignin solubilization, the Ch^+^ present in ChCl-based DESs contains a hydroxyl group capable of forming hydrogen bonds.^308^ These interactions can stabilize the crystalline structure of cellulose, thereby impeding its dissolution.^308^ As such, the presence of hydroxyl groups in the solvent, particularly on the choline cation, may limit the effectiveness of cellulose solubilization.^308^

Another possible mechanism, as proposed by Schutyser et al.,^309^ involves three essential steps. First, as shown in Figure 10, the benzyl carbon (Cα) is Cβ deprotonated, followed by hydrolysis leading to cleavage. Second, there is dehydration and formation of an active (Cα) cation and finally, the ether bonds are cleaved in either of two possible routes, depending on the acid used; loss of formaldehyde from C_β_ and deprotonation at Cα (predominates when using H_2_SO_4_) or protonation at C_β_ (predominates when using HCl/HBr). The intermediates resulting from β–O–4 aryl ether cleavage could also react with one another, thereby forming condensed lignin, and depending on the DES type and biomass type, C—C bonds in lignin may also undergo cleavage.^291^ However, the precise mechanism of cleavage of DESs remains unclear. Hemicellulose can be further depolymerized into oligomers and monomers, whereas cellulose is often recalcitrant.

**Figure 10:**
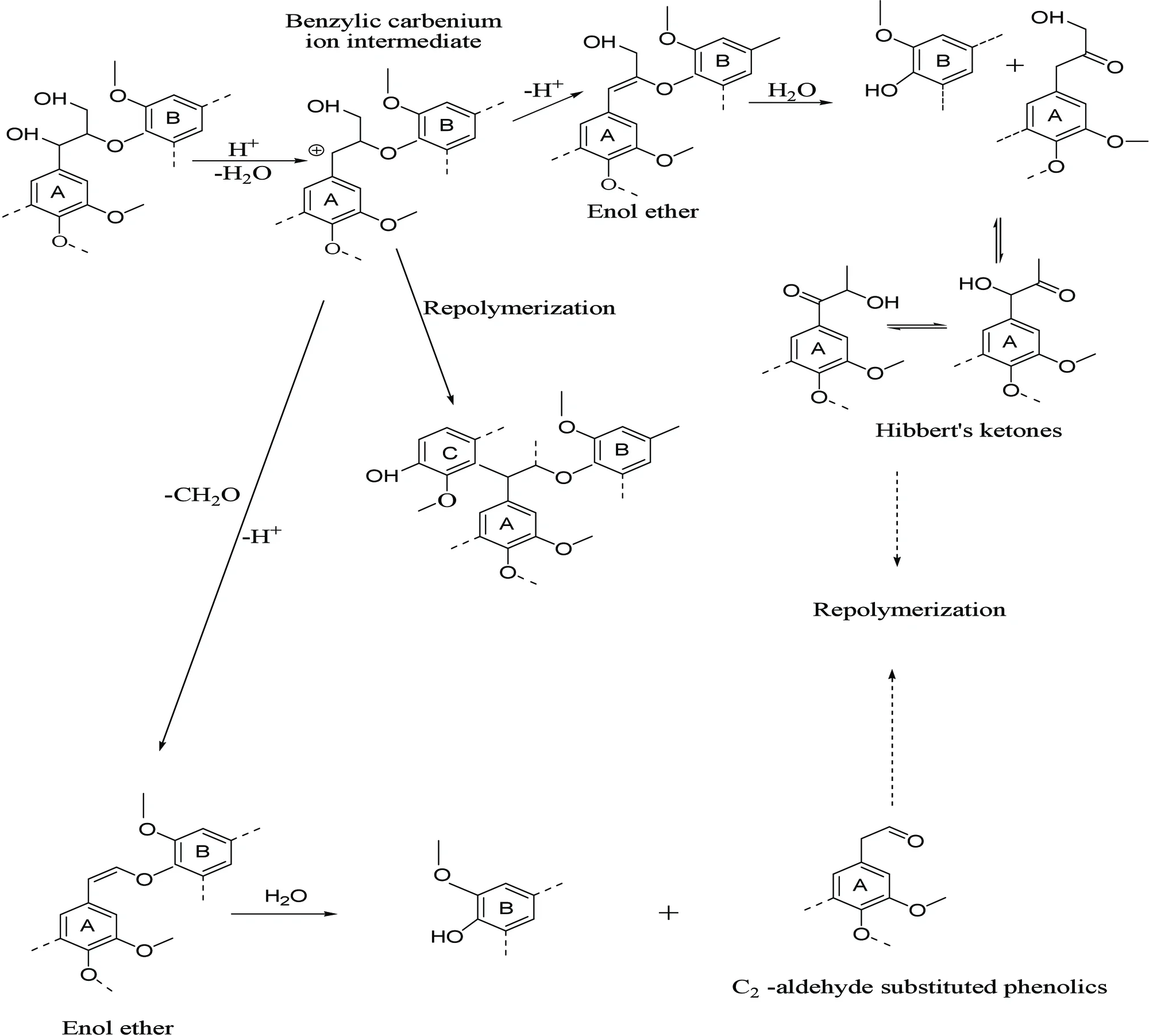
Acid-catalyzed lignin mechanism of the cleavage of the β–O–4 compound, where A,B,C represent phenolic rings.^309^ Reproduced from ref 309 with permission from the Royal Society of Chemistry. Copyright 2018 The Royal Society of Chemistry.

## 5 Enzymatic Hydrolysis

For biorefinery applications, the fundamental objective of lignocellulosic pretreatment is to reduce its recalcitrance with a subsequent increase in fermentable sugar yield, which is referred to as enzymatic hydrolysis. Biofuel production, especially the second generation, includes three major steps: pretreatment, which alters the biomass structure and chemical composition to enable efficient enzyme access and hydrolysis to fermentable sugars.^325^ Hydrolysis converts plant polysaccharides to monomeric sugars; and in the fermentation stage, these sugars are fermented into liquid fuels using microorganisms that are natural or genetically modified.^326^ Hydrolysis can occur chemically (using acids) or enzymatically (using hemicellulolytic enzymes).^327^ The latter is preferred as it requires less energy and mild environmental conditions and produces fewer fermentation inhibitors.^328^ Enzymatic hydrolysis is complex; the lignocellulose structural features make it recalcitrant, and some enzymes can be absorbed by condensed lignin, which lowers the hydrolysis yield by nonspecific linkages of these enzymes.^329^ Enzymatic hydrolysis of pretreated lignocellulosic biomass is influenced by enzyme and substrate-related parameters.^17^ DES hydrogen bond interactions may stabilize or activate enzymes in DES to hasten the reactions and enhance sugar yields, while minimizing pretreatment time, a favorable property in industrial-scale operation processes.^17^ When Kulshrestha et al.^330^ investigated the menthol/lactic acid pretreatment of microalgal biomass and determined a subsequent 3.2-fold high enzymatic hydrolysis, the results showed the highest sugar yield of 85% and discovered that DES reduces the pretreatment time. Different DESs have distinct effects on enzymatic saccharification, resulting in better product yields, as shown in Table 15. Furthermore, the biomass and substrate type, pretreatment temperature, and HBA: HBD molar ratio significantly affected enzymatic hydrolysis.

**Table 15:** DES Pretreatment Efficiency of Enzymatic Hydrolysis

Biomass	DES	Conditions	Enzymatic Hydrolysis Efficiency	Ref
Rice husk	-	50 °C, 0.5 h	0.2 mM glucose	^336^
Rice husk	1:2 ChCl/glycerol	115 °C, 3 h	0.7 mM glucose	^336^
Corncob	Untreated	–	32.8% glucose, 15.5% xylose	^227^
Corncob	7:3 ChCl/glycerol	80 °C, 15 h	39.9% glucose, 17.7% xylose	^227^
Corncob	7:3 ChCl/glycerol	115 °C, 15 h	79.1% glucose, 61.3% xylose	^227^
Corncob	7:3 ChCl/glycerol	150 °C, 15 h	91.5% glucose, 95.5% xylose	^227^
Corncob	1:2 ChCl/urea	80 °C, 15 h	51.0% glucose, 22.9% xylose	^227^
Corncob	1:2 ChCl/urea	115 °C, 15 h	58.6% glucose, 31.2% xylose	^227^
Corncob	1:2 ChCl/imidazole	80 °C, 15 h	92.3% glucose, 59.5% xylose	^227^
Corncob	1:2 ChCl/imidazole	115 °C, 15 h	94.0% glucose, 84.0% xylose	^227^
Corncob	1:2 ChCl/imidazole	150 °C, 15 h	94.6 glucose, 84.8% xylose	^227^
Corncob	Untreated	–	22.1% glucose	^241^
Corncob	1:2 ChCl/lactic acid	90 °C,24 h	81.6% glucose	^241^
Corncob	1:5 ChCl/lactic acid	90 °C,24 h	83.5% glucose	^241^
Corncob	1:10 ChCl/lactic acid	90 °C,24 h	83.2% glucose	^241^
Corncob	1:15 ChCl/lactic acid	90 °C,24 h	79.1% glucose	^241^
Corncob	1:2 ChCl/glycolic acid	90 °C,24 h	67.3% glucose	^241^
Corncob	1:2 ChCl/levulinic acid	90 °C,24 h	62.0% glucose	^241^
Corncob	1:1 ChCl/malonic acid	90 °C,24 h	61.5% glucose	^241^
Corncob	1:1 ChCl/glutaric acid	90 °C,24 h	40.7% glucose	^241^
Corncob	1:1 ChCl/oxalic acid	90 °C,24 h	45.2% glucose	^241^
Corncob	1:1 ChCl/malic acid	90 °C,24 h	37.4% glucose	^241^
Corncob	1:1ChCl/ethylene glycol	90 °C, 24 h	85.3% glucose	^241^
Corncob	1:1 ChCl/glycerol	90 °C, 24 h	96.4% glucose	^241^
Rice straw	1:5 ChCl/lactic acid	60 °C, 12 h	36.0%	^184^
Oil palm trunk	-	–	25.0%	^337^
Oil palm trunk	1:2 Ethylammonium chloride/ethylene glycol	100 °C, 48 h	74.0%	^337^
Pine	Untreated	–	10.0%	^85^
Pine	1:2 ChCl/formic acid	155 °C, 2 h	70.0%	^85^
Microcrystalline cellulose	Untreated	–	49.0%	^335^
Microcrystalline cellulose	5:2 ChCl/boric acid	80 °C, 24 h	49.0%	^335^
Microcrystalline cellulose	1:1 ChCl/glycerol	80 °C, 24 h	∼65.0%	^335^
Microcrystalline cellulose	1:1 Betaine/glycerol	80 °C, 24 h	∼65.0%	^335^
Eucalyptus dissolving pulp	Untreated	–	62.0%	^335^
Eucalyptus dissolving pulp	1:1 ChCl/glycerol	80 °C, 24 h	∼100.0%	^335^
Eucalyptus dissolving pulp	5:2 ChCl/boric acid	80 °C, 24 h	∼100.0%	^335^
Eucalyptus dissolving pulp	1:1 Betaine/glycerol	80 °C, 24 h	∼100.0%	^335^
Wheat straw	Untreated	–	18.0%	^335^
Wheat straw	5:2 ChCl/boric acid	80 °C, 24 h	33.0%	^335^
Wheat straw	1:1 ChCl/glycerol	80 °C, 24 h	<20.0%	^335^
Wheat straw	1:1 Betaine/glycerol	80 °C, 24 h	<20.0%	^335^
Spruce saw dust	Untreated	–	8.0%	^335^
Spruce saw dust	1:1 ChCl/glycerol	80 °C, 24 h	<20.0%	^335^
Spruce saw dust	5:2 ChCl/boric acid	80 °C, 24 h	<20.0%	^335^
Spruce saw dust	1:1 Betaine/glycerol	80 °C, 24 h	<20.0%	^335^
Switchgrass	1:1 ChCl/4-hydroxybenzyl alcohol	160 °C, 3 h	32.0% glucose, 19.2% xylose	^253^
Switchgrass	1:1 ChCl/catechol	160 °C, 3 h	77.0% glucose, 42.4% xylose	^253^
Switchgrass	1:2 ChCl/vanillin	160 °C, 3 h	79.8% glucose, 35.3% xylose	^253^
Switchgrass	1:1 ChCl/p-coumaric acid	160 °C, 3 h	85.7% glucose, 28.8% xylose	^253^

### 5.1 DESs-Enzyme Biocompatibility

Recent research has shown that enzymes can also be incorporated into the pretreatment stage to reduce the pretreatment time. DESs have good compatibility with enzymes and microorganisms and are used as solvents for subsequent enzymatic hydrolysis and/or fermentation.^124^ This phenomenon was adapted from one-pot ionic liquid pretreatments, in which three stages were carried out in the same vessel.^124^ Xu et al.^331^ designed a one-pot pretreatment system of aqueous choline-based ILs that allowed over 75% ethanol yield with 30–34% solid loadings of corn stover and high enzyme loading of 20 mg/g glucan. Individual DES constituents, such as choline chloride and urea, can inactivate proteins, but when combined to form a DES, inactivation can be lowered several-fold.^332^ Gunny et al.^333^ studied the stability and synergistic action of ChCl/ethylene glycol for the digestion of rice husk. The results showed that 90% of cellulase activity was retained after 1 h of incubation with different concentrations of ChCl/ethylene glycol at 30 °C. Therefore, the biomass pretreatment step, which is rather energy- and time-consuming, requires high infrastructure and high use of chemicals, and can be replaced by dilution of the DES/biomass solution with the buffer solution. The regenerated cellulose recovery step can be replaced by simple in situ hydrolysis of cellulose/hemicellulose by cellulases or xylanases.^334^ The consolidated ultrasound-assisted ChCl/sorbitol pretreatment of *Parthenium hysterophorus* biomass developed by Nargotra et al.^334^ and a sequential in situ enzymatic hydrolysis and fermentation showed high sugar and ethanol yield of 148.54 mg/g biomass and 110 mg/g biomass, respectively.

### 5.2 Factors That Influence Enzymatic Hydrolysis of DES Pretreated Biomass

#### 5.2.1 Type of Biomass and DES

The increase in hydrolysis yield after DES pretreatment of biomass is primarily influenced by the disruption of crystalline cellulose and lignin removal.^312^ Each biomass resource had a different distribution of lignocellulosic components, which affected the hydrolysis yield. Following an extensive investigation of 5:2 ChCl/boric acid, 1:1 ChCl/glycerol, and 1:1 betaine/glycerol pretreatments of four biomass types (kraft-dissolving eucalyptus pulp, cellulose, wheat straw, and spruce sawdust), Wahlstrom et al.^335^ discovered that the hydrolysis yield increased for kraft-dissolving eucalyptus pulp (62% to ∼100%), cellulose (49% to ∼65%), wheat straw (18% to 33%), and spruce saw dust (marginal increase from 8% for all DESs). DES pretreatments were effective with agricultural biomass such as wheat straw and low-recalcitrant substrates such as amorphous cellulose and kraft-dissolving eucalyptus pulp. Highly recalcitrant woody biomass, such as spruce sawdust, has a subpar enzymatic hydrolysis efficiency.^335^ DES constituents also influence enzymatic hydrolysis of pretreated biomass. 5:2 ChCl/boric acid had better efficiency than 1:1 ChCl/glycerol and 1:1 betaine/glycerol. For wheat straw, ChCl/boric acid had 33% efficiency, whereas 1:1 ChCl/glycerol and 1:1 betaine/glycerol had less than 20% efficiency.^335^ In another study, different ChCl/acid-based DESs showed that the acidity coefficient greatly influences enzymatic hydrolysis. ChCl/formic acid (p*K*_a_ 3.75) > ChCl/lactic acid (p*K*_a_ 3.86) > ChCl/acetic acid (p*K*_a_ 4.75) were used to pretreat pine residues, revealing that ChCl/formic acid had the highest hydrolysis yield of 70%.^85^ In another study by Zhang et al.^241^ where corncob was treated with monocarboxylic-based, dicarboxylic-based, and polyalcohol-based DESs. Monocarboxylic-based DESs showed the highest glucose yield (83.5%) compared to dicarboxylic-based DESs, which was due to the high p*K*_a_ values of dicarboxylic-based DESs. The polyalcohol-based DESs, on the other hand, showed great efficiency in glucose yield due to their hydroxyl groups, which interact with the hydroxyl groups of lignin, and their low viscosity. Another DES property that enhances enzymatic hydrolysis is the HBA/HBD molar ratio. Using 1:2, 1:5. 1:10 and 1:15 ChCl/lactic acid pretreatment on corncob, Zhang et al.^241^ discovered that the yield slightly increased with increasing molar ratio and the decrease at 1:10 and 1:15. Fundamentally, agricultural and low-recalcitrant feedstock respond better to DES pretreatment compared to woody bioresource. The DES type and acidity influence performance, though results may vary with solvent ratios and biomass type. These findings remain limited to lab-scale experiments and lack assessment of process scalability, which is essential for industrial applications.

#### 5.2.2 Pretreatment Severity

A high pretreatment temperature has been shown to enhance the enzymatic hydrolysis of pretreated biomass. Procentese et al.^227^ achieved the highest enzymatic hydrolysis yield of 91.5% at 150 °C after ChCl/glycerol pretreatment of corncob at temperature of 80 °C (39.9%) and 115 °C (79.1%). When varying the HBD, they noticed that the ChCl/imidazole ratio only had a marginal increase (0.6%) at the highest temperature. However, even at the lowest temperature of 80 °C, the hydrolysis yields were equally good (92.3% and 94% at 80 and 115 °C, respectively). In contrast, the hydrolysis yield of ChCl/urea-pretreated biomass (corncob) increased from 51% to 58.6% as the temperature increased from 80 to 115 °C. Zhang et al.^241^ studied the ChCl/lactic acid pretreatment of corncob at a temperature range of 70–110 and 90 °C was found to be the optimal temperature for glucose yield (78.7%). At a temperature beyond 90 °C, the glucose yield was constant due to balance effects of greater losses of polysaccharides and enhanced enzymatic hydrolysis digestion. The increase in hydrolysis yield was also affected by pretreatment time. The same study by Zhang et al.^241^ showed that elongation of the pretreatment time from 1 h 30 min to 36 h increased delignification and subsequent enzymatic hydrolysis due to prolonged contact of the DES and the substrate. Twenty-four hours was deemed the optimal pretreatment time, and there was only a marginal increase in the hydrolysis yield from 24 to 36 h. Overall, while temperature and reaction time are critical in improving the enzymatic hydrolysis of DES-pretreated biomass depending on the DES composition, excessively high temperatures or prolonged treatment time can lead to inhibitor formation which increases sugar degradation and inhibits downstream processes. Process optimization is important to balance thermal energy input for optimal hydrolysis yield.

## 6 Biofuel Production

The overexploitation of fossil fuels has prompted the global production of biofuels, such as bioethanol and biodiesel, in an attempt to replace current nonrenewable resources. Fermentation is the conversion of monomeric sugars obtained from enzymatic hydrolysis into bioproducts using microorganisms obtained naturally or genetically^338^ such as *Saccharomyces cerevisiae*, *Pichia stipitis*, *Saccharum spontaneum*, *Escherichia coli*, *Zymomonas mobiliz*, *Kluyveromyces marxianus*, and *Candida brassicae*.^17,334,339,340^ The most studied microorganisms among these is the yeast *Saccharomyces cerevisiae* due to its inherent pH, high temperature, and inhibitor resistance.^341^ It is important that fermentation microorganisms are temperature-resistant because of the high temperature required for enzymatic hydrolysis.^326^ For integral processes, the fermentation temperature should be as close as possible to the optimal enzymatic hydrolysis temperature as possible.^326^ Alternatively, for xylitol and butanol production, hemicellulose hydrolysate can be used for xylose fermentation.^326^ Bioethanol production from the fermentation of sugar hydrolysate obtained from DES-pretreated biomass has been reported in numerous studies. Vaid et al.^302^ ran a consolidated one-pot pretreatment of calcium hydroxide-assisted ChCl/glycerol on *Saccharum spontaneum* biomass and reported a bioethanol production yield of 173.61 mg/g. In another study by Xu et al.,^311^ 149 g/kg of bioethanol was produced from ChCl/glycerol pretreatment of corn stover, with a 77.5% theoretical yield based on the initial glucose present in the biomass samples. Similarly, Okuofu et al.^342^ Bambara groundnut haulmat biomass produced bioethanol concentrations of 11.57 g/L and 0.38 g/gL sugar after fermentation with *Saccharomyces cerevisiae* BY4743 yeast.

### DESs for Biodiesel Production

Another biofuel recognized as efficient and sustainable for curbing the global energy crisis, especially the increasing CO_2_ emissions from the diesel-based transportation industry, is biodiesel. The conversion of waste oils to diesel occurs via transesterification.^343^ However, there are shortcomings associated with this process and other chemical-based processes, such as lipid/biomass extraction, pretreatment, and purification.^344^ These disadvantages include emulsification and corrosion, high waste treatment, high costs, nonbiodegradability, toxicity, and energy-intensive procedures for the extraction of lipids.^17^ DESs can be applied in biodiesel chemical and enzymatic catalytic processing and for removing impurities, such as glycerol, from diesel. Gu et al.^345^ reported the successful transesterification of rapeseed oil using 1:2 ChCl/glycerol; 98% of fatty acid methyl ester (FAME) yield, from which biodiesel quality fuel can be obtained. Similarly, Liu and Wang^346^ studied 3:1 ChCl/*p*-toluenesulfonic acid DES as a heterogeneous acid catalyst in the transesterification of soybean oil (8 wt % relative to oil) with methanol (8:1 relative to oil) to produce biodiesel. The transesterification yield could reach 98.66% under optimized conditions. Moreover, due to the DES’s heterogeneity, product separation and catalyst recovery were easy.^346^ DESs have been explored as extraction solvents in biodiesel production. Eighteen ChCl- and methyltriphenylphosphonium bromide (MTPB)-based DESs were prepared and applied for the removal of residual catalyst KOH from palm oil-based biodiesel.^347^ Glycerol-based DESs exhibited the highest extraction efficiency, removing 98.59% (ChCl) and 97.57% (MTPB) of KOH.^347^ Moreover, the DESs effectively reduced the water content in biodiesel to meet international quality standards and demonstrated good reusability over five cycles with only minimal loss in performance.^347^ DES pretreatment of microalgal biomass produces higher lipid yields. A maximum lipid yield of 19.25% was obtained from the ChCl/acetic acid DES of *Chlorella pyrenoidosa* biomass. In another study, Pan et al.^348^ performed a ChCl/acetic acid pretreatment, with wet and unbroken algal biomass of *Chlorella* sp. and *Chlorococcum* sp. for lipid extraction with a 30% improvement in the FAME yield in a consolidated method. The unpretreated *Chlorella* sp. lipid recovery is 52.03% and increases to 80.90%, 66.92%, and 75.26% lipid recovery for ChCl/oxalic acid, ChCl/ethylene glycol, and urea/acetamide pretreatments, respectively. Similarly, Wichaphian et al.^349^ demonstrated a zero-waste biorefinery approach using ultrasonically assisted ChCl/acetic acid DES pretreatment of *Chlorella* biomass, achieving a high lipid yield of 139.5 mg/g and producing biodiesel that met biodiesel quality standards, while deoiled biomass residue was repurposed as a hydroponic biofertilizer. Vegetable oils can also be used for biodiesel production via DES pretreatment. Recently, He et al.^213^ reported that the biodiesel yield increased from 91.8% to 98.5% after the ChCl/glucose DES addition to liquid lipase *Yarrowia lipolytica* system, with optimized conditions. To further examine the effect of DES on the enhancement of lipase performance, they also studied the transesterification of DES alone and lipase with each DES component; DES + lipase showed better performance. Additionally, DES improved the enzyme reusability with 99.6% biodiesel yield in the second reuse trial. These findings suggest that DESs are promising solvents for biodiesel production via transesterification in various ways, such as cosolvents, catalysts, and solvating promoters. The commercial and economic aspects of this framework must be studied. Life cycle analysis, techno-economic analysis, exergetic life cycle assessment, and energy analysis of bioenergy production can provide sufficient evidence for large-scale industrial application of biofuel production in the future to develop novel and sustainable technologies for biofuel production.

## 7 Recycling and Recovery of DESs

The recovery and reuse of DESs are one of the favorable advantages that warrant their application in low-cost, high-volume industrial uses such as biomass processing.^350^ Economically and environmentally, DESs recycling is important because it minimizes the overall cost of pretreatment processing by reducing waste steam generation and chemical consumption.^21^ A large amount of water is required to wash the DES-pretreated lignocellulosic biomass and precipitate lignin, resulting in the dilution of DES and formation of hydrated DES mixtures, which must be separated prior to reuse. This involves recovering (separation), refining (purification), and reuse in successive stages.^17^

DES recycling is easier than ILs recycling because their synthesis and regeneration do not involve chemical reactions and only involve the formation or disruption of the hydrogen bond network between DES constituents (HBA and HBD).^17,124^ However, this does not imply that the process is straightforward, particularly when large quantities of water of volatile solvents are involved in separation and purification stages. Pretreatment studies have rarely investigated the recyclability of DESs,^257^ although it is widely recognized as an important research parameter for the commercial usefulness of biomass-based biorefinery.^124^ A few recovery methods to recover DESs have been introduced in the past: evaporation/distillation, addition of antisolvent, membrane filtration, crystallization, solid–liquid extraction, liquid–liquid extraction, density separation, and supercritical fluid extraction,^17,184,351,352^ summarized in Table 16.

**Table 16:** Comparison of DES Recovery Methods

Recovery Strategy	Principle	Advantage	Limitation	Effect on DES Pretreatment
Antisolvent	Water, acetone, or ethanol added to precipitate solutes	Fast, simple, industrially scalable; allows fractionation of lignin/hemicellulose	High energy for solvent removal; requires high antisolvent concentrations	Preserves DES activity if careful; may require multiple steps for fractionation
Evaporation/distillation	Water or solvent is evaporated from hydrated DES mixture	Simple and widely applicable; high recovery efficiency; maintains DES structure and activity; multiple reuse cycles possible	Energy-intensive for large volumes; less effective for dilute or highly viscous DESs	Recycled DES maintains performance, glucan digestibility, and glucose yield even after multiple cycles
Recrystallization	Water precipitates biomass; centrifugation isolates solid particles; acetone separates DES and water; water is evaporated	Can recover DES as solid; does not require chemical reactions; allows selective recovery of components; adaptable to hydrated DES	Multistep process; requires vacuum evaporation; energy input needed for drying; may be slower than simpler methods	DES can be recovered with minimal loss in activity, maintains performance if impurities are controlled
Solid–liquid extraction	Adsorption of DES components on macroporous resin; desorption using water to break hydrogen bonds and facilitate separation	High recovery efficiency; selective recovery; effective for complex mixtures	Expensive resins; high energy consumption; limited commercial applications; scale-up challenges	Preserves DES performance; allows repeated use with minimal loss of activity in lab-scale studies
Liquid–liquid extraction	Immiscible organic solvent is used to selectively separate DES components based on their differential solubility.	Allows selective recovery of DES components; simple separation via decanting; effective for biomass-derived solutes	Requires potentially toxic organic solvents, handling of solvents adds complexity; additional solvent recovery may be needed	Maintains DES performance if residual solvents are removed properly; can prevent performance inhibition caused by contaminants
Supercritical fluid	Uses supercritical CO_2_ to separate DES from extracted compounds; uses expansion for product	Nontoxic and nonflammable; moderate critical temperature/pressure; simple product separation; gas can be recycled	High energy consumption; high-pressure operation; expensive; limited commercial applicability	Potential for DES recycling without chemical degradation; effectiveness demonstrated for target compound recovery
Density separation	Separation based on density and viscosity differences using centrifugation	Simple process; easy scale-up; no additional energy required; minimal equipment	Only applicable when feedstock and DES have significant density/viscosity differences; limited to certain mixtures	Maintains DES efficiency over multiple cycles; allows repeated use in esterification and biodiesel production
Integral methods	Combines different methods to fractionate solutes and recover DES	Can selectively isolate multiple components; high recovery potential	Complex operation; high energy consumption; expensive membranes; multiple steps; may introduce impurities	Reduced efficacy due to impurities; decreases enzymatic hydrolysis or delignification capacity after repeated cycles

These are applied in the recovery of DES, regardless of DES application, and not necessarily in biomass processing. We can certainly take guidance from other academic and industrial studies that have utilized DESs and recycled them in different fields. For example, simple filtration of the catalyst was used to recover ChCl/urea after the epoxide reaction, and ChCl/urea was recycled several times with a marginal decrease in activity.^353^ In another study on nucleophilic substitution with nucleophiles, ChCl/ZnCl_2_ was recovered using water extraction and vacuum drying four times with no loss of activity.^354^ Among these recovery methods, evaporation/distillation is the most commonly used method for recycling and recovering DESs in the biomass pretreatment process.^17^ Yan et al.^351^ studied the ChCl/oxalic acid pretreatment of corncob biomass and after recycling ten times using evaporation, the recycled DES maintained the glucan digestibility and glucose recovery. After three cycles of pretreatment with ChCl/lactic acid and betaine/lactic acid recycling by evaporation, Kumar et al.^184^ observed no significant loss of DES reusability strength. Recently, Kim et al.^253^ investigated the recovery, recyclability, and subsequent efficacy of choline-chloride-based DESs during the pretreatment of switchgrass by rotary evaporation. Mass balance analysis showed that approximately 95% of DES was recovered after three sequential cycles with no loss of efficiency.

The antisolvent addition method is the simplest and fastest method for DES recovery.^17^ The commonly used antisolvents are water, acetone, and ethanol, all of which can be recovered from the DES or its aqueous mixtures through evaporation, and the DES can be recycled back into the system.^355^ Antisolvents are usually added at high concentrations to assist in the disruption of the hydrogen bonds between HBAs and HBDs, thereby precipitating the solubilized components of the pretreated lignocellulosic biomass.^17^ Owing to the volatility of antisolvents, they can be easily covered through evaporation, centrifugation, and/or precipitation.^355^ These processes are well-known unit operations available on a commercial scale, which merit highlighting. After the pretreatment of dry beech wood sawdust using ChCl/oxalic acid, Mamilla et al.^356^ used acetone as an antisolvent to recover DES from the reaction mixture. The DES was obtained in solid form after the remaining mixture of water and the DES was evaporated to remove water. Similarly, water was added as an antisolvent to recycle ChCl/lactic acid after pretreatment of rice straw biomass, and the recycled DES exhibited excellent delignification strength.^357^ Antisolvents can be used sequentially to fractionate each lignocellulose component; water is usually used to precipitate lignin, while ethanol is used to precipitate hemicellulose-rich fractions. Li et al.^357^ studied the ChCl/lactic acid pretreatment of rice straw and used water to fractionate 64.8% lignin, and 3 volumes of 96% ethanol to regenerate hemicellulose-rich fractions. One of the notable shortcomings of this method is the relatively high energy required for recovering DES from hydrated mixtures, because the process uses high concentrations of antisolvents.

The Mamilla et al.^356^ study is also a good example of the recrystallization/extraction recovery method using an antisolvent. Water was used to precipitate the dissolved biomass, followed by centrifugation to isolate the solid particles. Subsequently, acetone was sequentially added to separate the deep eutectic solvent (DES) from the reaction mixture, resulting in the formation of two distinct layers: a DES-rich layer and a product-rich layer. The water was then evaporated under vacuum, and the DES was recovered as a solid.^356^ Either one or both DES constituents can recrystallize by disrupting their networks. The core principle of this process is that DES preparation does not involve chemical reactions. In cases where DES is hydrated due to processing with water, selective disruption of hydrogen bonding can still enable recovery of individual components. Therefore, introducing any agent capable of disrupting intermolecular interactions can lead to the recrystallization of one or both components of DES,^355^ and the recrystallized fractions can be separated by filtration or centrifugation. Yu et al.^358^ designed a catalytic system (ChCl/formic acid, SnCl_4_·5H_2_O) for the conversion of furfural derivatives from lignocellulosic biomass at 140 °C. As the system cooled to room temperature, SnCl_4_^–^ disrupted the intermolecular interactions between ChCl and formic acid, leading to the formation of a white precipitate, ChCl.

Another effective method for recovering DES is liquid–liquid extraction. This approach uses an immiscible organic solvent to selectively separate DES components based on their differential solubility. The process capitalizes on the distinct chemical affinities between the DES and the immiscible organic solvent,^355^ allowing the target components to migrate into the solvent phase and facilitating efficient separation and recovery. The separation of the organic solvent from the DES is simple and can be performed by decanting. It is generally used to separate solutes from DES after biomass conversions.^355^ This method has been well explored for the conversion of lignocellulose to furfural derivatives^359–362^ using different organic solvents, such as methyl isobutyl ketone, hexane, 1-butanol, ethyl acetate, diethyl ether, 2-methyltetrahydrofuran, and ethyl *n*-butyrate.^355^ Researchers have also studied the separation of DES from waste oil-based biodiesel production using *n*-hexane and subsequently 1-butanol to remove the excess glycerol that hinders the recycled DES performance.^363^ Xu et al.^364^ tested a 2-stage liquid–liquid extraction to recover citrus peel waste flavonoids from ChCl/levulinic acid/*N*-methyl urea using ethyl acetate and *n*-butanol, yielding 95.9% and 86.3% of polymethoxylated flavonoids and glycosides of flavonoids, respectively. In addition to a DES recycling method, Smink et al.^365^ discovered that this process can be used as an alternative to an antisolvent to separate lignin from DES and found that cold water precipitation yielded a heavy molar weight fraction, and a combination of both approaches is important for full lignin recovery in industrial applications.^365^ One significant drawback of this recovery method is the toxicity of organic solvents, such as hexane.

Another effective method for recovering DES is solid–liquid extraction using a macroporous resin, which is based on the adsorption of target compounds in a liquid mixture on solid macroporous resin.^355^ This method leverages the unique properties of macroporous resins to selectively adsorb and subsequently desorb DES from mixtures. Essentially, the DES-containing system passes through a column packed with macroporous resin, which captures the DES constituents only. After the adsorption phase, the DES was separated from the resin by eluting the water through the column. Water can break the hydrogen bonds between the components of DES and enhance DES separation.^366^ The DES is removed from the column using a washing liquid, which can be removed from the DES through evaporation. Despite the requirement for expensive macroporous resins, limited applications, limited commercial usage, and high energy consumption,^355^ they yield optimal DES recovery results. Using simultaneous ultrasound/microwave-assisted extraction, grape pomace anthocyanins were extracted using ChCl/citric acid. A total of 96.8% of DES was recovered using solid–liquid extraction. Grillo et al.^366^ reported 79.5% DES recovery after using Sepabeads SP 825 L macroporous resins for extracting anthocyanins from blueberry peels.

Recently, Plaza et al.^367^ discovered the application of supercritical fluids as a DES-recovery approach. Two hydrophilic DESs, 1:1 ChCl/citric acid and 1:2 ChCl/ethylene glycol, were used to extract bioactive compounds from olive mill waste and leaves. Supercritical CO_2_ was used to recover the target compounds from the DES extracts: 81.1% and 57% from olive mill waste and olive leaf extracts, respectively. Even though recycling experiments were not conducted, the supercritical fluid approach effectively retrieved the target hydroxytyrosol, tyrosol, and oleupein while regenerating DES that could be recycled and reused within the system. Supercritical carbon dioxide is frequently utilized because of its nontoxic and nonflammable nature and relatively moderate critical temperature and pressure.^368^ In addition to the absence of toxic substances, the primary advantage of supercritical fluid extraction is its ability to facilitate the easy separation and drying of the product through simple expansion. Furthermore, the gas used in the process can be efficiently recovered, recycled, and reused, thereby eliminating the need for additional purification steps.^369^ However, factors such as high energy consumption, complex processes that require high pressure, high costs, and limited applicability limit their application at the commercial stage and in DES-related processes.^355^

In a case where the liquid feedstock and the DES have different density and viscosity, DES can be simply recovered by separation, usually by centrifugation. Hayyan et al.^370^ explored a laboratory-scale two-stage process for the bioproduction of low-grade crude palm oil (LGCPO)-based diesel. Owing to the density and viscosity differences, centrifugation was used to recover the DES from the mixture, and the DES was recycled four times for esterification without loss of activity. The same group further explored this process by evaluating the effects of a different salt (*N*,*N*-diethylenethanol ammonium chloride) as a novel recyclable catalyst. As the DES and treated palm oil exhibited different densities and viscosities, the DES was recovered from the mixture by centrifugation. Four recycling runs were performed, and a slight change in the biodiesel yield was observed after the second run.^371^ They also studied ChCl as an HBA to enhance their study and recorded a 1% decrease in DES efficiency after three recycling runs.^372^ This explains the influence of DES constituents on their efficiency. The merits of this method are highlighted by its simple process and scale-up, no additional energy requirement, and the absence of toxic substances.

The integration of two or more recovery methods is another method for recovering biomass fractions from DES systems.^355^ Typically, integrated methods involve antisolvent addition and membrane-based processes such as ultrafiltration, dialysis, and electrodialysis.^355^ These are versatile separation techniques that selectively fractionate solutes and recycle of permeates. The aforementioned methods are mostly used for DES recovery after pretreatment of biomass.^355^ For instance, Wahlstrom et al.^373^ used an integral system of an antisolvent and dialysis to separate and concentrate DES-extracted protein from brewer’s spent grain (BSG). The solids were then regenerated from the extract and washed with water. The wash water and extract were combined prior to dialysis. The maximum protein extraction yield was 79%; however, dialysis reduced it to 42%. The DES recovery and recycling were not evaluated in this study. Kim et al.^253^ studied the ultrafiltration/antisolvent integral system to recover ChCl/*p*-coumaric from switchgrass pretreatment. Ethanol and water were used to precipitate the cellulose-rich fraction. The DES-ethanol/water mixture was subjected to ultrafiltration to isolate the lignin. A total of 95% of the DES was recovered. To study DES recycling efficacy, the enzymatic hydrolysis yields of two runs of recycled DES were investigated. The yield decreased from 87% to 78% and 70%, for second and third trials, respectively. This decrease was attributed to impurities from the decomposition of carbohydrates and phenolic compounds from lignin after each run. Liang et al.^374^ used a 3 method integral system (antisolvent, ultrafiltration, and electrodialysis) to recover ChCl/ethylene glycol after a 2 stage pretreatment (hydrothermal and DES pretreatment methods). Water was used to regenerate lignin, which was then removed from the DES mixture by ultrafiltration. The filtrate and DES constituents were separated by electrodialysis. To evaluate the recovery and recyclability of DES, DES was resynthesized using the recovered DES constituents, and the recovered DES decreased the delignification capacity. Other integral recovery methods explored are distillation with liquid–liquid extraction^262^ to recover 89% of DES-produced furfural from beechwood xylan. Three recycling runs were achieved, with over 69% loss in furfural yield.^262^ Integral methods are complex, require more energy, and have high investment costs owing to the high membrane costs.

Some studies have shown that recycled DESs reduce the efficacy of biomass pretreatment and subsequent pretreatment cycles due to contaminants and minerals from the biomass, which can change the DES properties.^17^ Furthermore, the thermal instability and susceptibility to biomass impurities that alter the hydrogen bonding between DES constituents inhibit the application and recycling of DESs.^92^ Shen et al.^279^ pretreated Eucalyptus biomass using ChCl/lactic acid DES and recovered approximately 90% of the used DES with unaltered structures. However, after four cycles, the pretreatment performance decreased from 94.3% to 73.8%. In another study by Chen et al.^289^ to study the efficacy of phenyl-based DES (ChCl/p-coumaric acid) pretreatment of herbal residues, using ethanol as an antisolvent. The recycled DES showed poor pretreatment performance and a significant decrease in enzymatic hydrolysis yields. Although several promising DES recovery strategies have been demonstrated, each comes with inherent challenges which limit scalability. For example, thermal stability of DESs is a crucial factor influencing their recyclability and reuse. While DESs are generally thermally stable, as discussed in Section 4.3.1, for DES pretreatment methods carried out at temperatures exceeding 100 °C, the prolonged heated and repeated cycles at elevated temperatures may lead to gradual degradation. For DESs to become viable at commercial scale, further research must prioritize recovery strategies that are energy-efficient, cost-effective, and recyclable in larger recycle numbers and thermally stable DESs to account for future biorefinery applications.

## 8 Techno-economic Analysis and Life Cycle Assessment

Although DESs have been extensively studied, their feasibility and profitability have rarely been explored. For DES-based biorefineries to be considered sustainable, they must be environmentally favorable and technically scalable at the industrial level. To determine the sustainability potential of DESs in biorefinery processes and guidance for further process optimization, techno-economic assessments (TEA) and life-cycle assessment (LCA) have been employed.^186^ TEA uses computer simulations to evaluate economic indicators such as capital and operational expenditure, net present value, and payback period.^186^ The TEA of biorefinery processes using various solvents has been investigated extensively in prior research. However, there is a noticeable gap in the literature concerning TEA of DES-based bioproducts. Kumar et al.^375^ first advanced the TEA designs for NADES-based biorefinery in 2020, using a 1 ton per day capacity design plant with three key parameters; payback period, net present value (NPV) and internal rate of return (IRR). Interestingly, instead of only using simulated data, their evaluation was based on real-life scenarios where the “*n*th” plant approach was used, “*n*” representing the number of plants in an operation. They evaluated different biorefinery and design scenarios based on several metrics, and the capital and operational expenditures thereof. Their work proved that low-volume, multivalue-added products are more cost-effective for biobased biorefineries than low-, high-, and single-product-based biorefineries. In the projected scenario, multivalue-added products (cellulose, lignin, and xylan) generated an NPV of 1.4 million USD, >100% IRR, and a payment period of <2 years. Moreover, the sensitivity analysis showed that of all value-added products, lignin was the most dynamic product in terms of pricing and application, followed by xylan. Furthermore, the recycling and reuse of NADESs for subsequent pretreatments reduces operational expenditure, which speaks to their economic feasibility and profitability.

Similarly, Zhao et al.^376^ discovered that DES recycling influences the operating costs owing to the reduced cost of the material. The total capital investment (TCI) decreased from $659.43 million to $612.74 million when the number of cycles increased from 5 to 20. Peng et al.^278^ also conducted an economic evaluation using an Aspen plus simulator with sensitivity analysis to study the techno-economic viability of ChCl/urea and ChCl/lactic acid for the bioproduction of ethanol and lignin as a byproduct from rice straw. The key parameters used to evaluate the construction and operation of a bioethanol plant are investment and operation costs, IRR, NPV, and minimum ethanol selling price (MESP). Sensitivity data were used to determine the factors influencing MESP. Different pretreatment temperatures were considered for ChCl/urea (110, 120, and 130 °C), while it was maintained at 121 °C for ChCl/LA to understand the economic feasibility of the two DESs. ChCl/LA produced a higher bioethanol yield (0.21 t/t) than ChCl/urea (0.166 t/t, 0.17 t/t, and 0.168 t/t for temperatures 110 °C, 120, and 130 °C, respectively). Moreover, ChCl/LA had less energy consumption, and its MESP was low at 2128 $/ton owing to the advantages of low TCI, low total operating cost (TOC), good pretreatment performance, and high solid loading (20%), which further proved that the economic advantages were influenced by pretreatment and raw materials. Although the ChCl/LA-pretreated MESP fluctuated owing to the enzymatic cellulose/conversion ratio, acetone price, and lactic acid price, sensitivity analysis concluded that using suitable enzymes, inexpensive washing solvents, and high solid loading pretreatment could reduce these fluctuations.

One study that agrees with these high solid loading findings is by Zhang et al.,^377^ who studied and compared high solid loadings (20% and 27%) of switchgrass using an aqueous DES (ChCl/ethylene glycol.H_2_O) for the bioproduction of butanediol and coproduction of furfural and technical lignin. They discovered that a 20% solid loading system had high mass and energy requirements. Techno-economic feasibility was studied using an Aspen Plus simulator and a modified National Renewable Energy Laboratory (NREL) economic model with minimum butanediol selling price (MBSP) as a key economic indicator. The MBDP ranges from $1703–1736/ton, which is approximately half of the current market price. Sensitivity analysis concluded that MBSP was influenced by the acetone/methyl isobutyl ketone recycling ratios, discount rate, and furfural prices, which suggests the need for technical improvements.

To assess the environmental viability of the biorefinery processes, LCA was used to quantify the environmental impacts of bioproducts obtained from different methodologies and sources, considering the life cycle of the systems. Ideally, a biorefinery should enhance its production efficiency and reduce environmental pollution. LCA has been explored for environmental evaluation using different solvents, but there have been few reports on DES-based bioproducts. Recently, Zargar et al.^378^ investigated the isolation of lignin-containing cellulose nanocrystals (LCNCs) from thermomechanical pulp (TMP) at a laboratory scale using DES binary (ChCl/oxalic acid.dihydrate) and ternary (ChCl/oxalic acid.dihydrate–*p*-toluenesulfonic acid) systems. They studied the environmental impacts of LCNCs using cradle-to-gate LCA for global warming potential (GWP), acidification potential (AP), and cumulative energy demand (MJ). Per kg of LCNCs, the results were 39 kg CO_2_-eq, 0.17 kg SO_2_-eq, and 995 MJ for GWP, AP, and energy use, respectively. In addition, varying the input volume and reusing the DES can further reduce the environmental impact. The potential of DES recycling and reuse, further reducing the environmental impact, was also reported by Guo et al.^379^ who investigated a comparative study of DES pretreatment and traditional alkali methods on wheat straw for partial swelling and subsequent improvement of structuring, cohesion, and durability of energy pellets. Xia et al.^380^ also reported a lower environmental impact of DES-based lignocellulosic bioplastics, merited by their ease of recycling and safe biodegradability in natural environments. In another study, using a ternary DES (choline chloride/1,3-butanediol/d-sorbitol) to extract bioactive polysaccharides from *poria cocos*, a laboratory-scale LCA was used to analyze the environmental impacts, and the results showed the environmental impact reductions of 93.61% waste solids and 69.72% fossil fuel depletion potential compared to the traditional method. Furthermore, the recovered DES maintained its performance after five cycles of dialysis.^381^ Another comparative analysis carried out to evaluate the environmental impacts was performed by Murugan et al.^382^ using DES and ethanol for the extraction of bioactive compounds from *Darcyodes rostrates.* Contrary to other positive reports, the environmental profile concluded that ethanol showed a lower environmental impact and energy consumption compared to DES. Transport activities and electricity consumption were the leading contributors to the high environmental impact. Both TEA and LCA are important for the crucial understanding of the economic and environmental implications at a commercial scale; integrating the two would be revolutionary to establish a solid foundation to advance technologies.^383^ However, there are still limitations with them individually that need to be addressed. For example, because DESs are relatively new solvents, their properties are unavailable in simulation software databases and therefore should be defined manually. For LCA, environmental impacts should go beyond climate change.^186^

Although DESs have shown considerable promise at the laboratory scale, there are no published LCA or TEA studies evaluating DES-based processes in real-world operational settings. Nonetheless, LCA and TEA have been extensively applied to biorefinery plants, providing critical insights into economic viability, environmental performance, and process optimization that can guide future scaling of DES-assisted biomass conversion technologies. Ostold Research^384^ did a cradle-to-gate assessment of the Sarpsborg Borregaard plant, quantifying the environmental performance of their products (cellulose, ethanol, lignin, and vanillin). The main environmental hotspots were associated with steam and energy production; steam is required in large quantities for the processing steps, making it a single largest source of CO_2_ emissions.^384^ However, a 2019 LCA update showed a slight improvement in CO_2_ footprint (i.e., cellulose GWP decrease from 1211 to 1160 kg) due to a second steam production plant using municipal waste.^385^ The CO_2_ footprint would have been significantly lower if Borregaard used its produced hydropower instead of outsourcing imported coal-based power.^59^ Moreover, due to steam requirement and energy-intensive chemical production, the Cumulative Energy Demand (CED) is high; vanillin ranking high with 36.5 kJ, followed by cellulose (33.0 kJ), powder lignin (31.5 kJ), liquid lignin (18.2 kJ), 99% ethanol (18.1 kJ), and 96% ethanol (8.7 kJ).^384^ Another key contributor to environmental impacts is waste production; vanillin recorded a high of 82.8 kg, the lowest being 96% ethanol at 26.8 kg,^384^ suggesting a correlation between CED and waste production. Finally, chemical production and consumption further contribute to environmental burdens as they increase the acidification potential, eutrophication potential, and photochemical ozone creation potential.^384^ From this LCA report, it is evident future DES-based biorefineries should be designed as fully integrated systems that coproduce multiple high-value products rather than a single commodity to maximize economic returns and be flexible in the processing line by adapting to different feedstocks to accommodate seasonal variations in feedstock supply. Furthermore, it is important to prioritize energy-efficient, zero-waste, and less expensive operations that use cogeneration, valorize the byproducts, and allow for high-solid loading to enhance profitability and reduce operating costs. Demonstration plants and regular LCA and TEA assessments are also important to identify environmental hotspots, economic risks and guiding process optimization and scaling for long-term sustainability.

## 9 Computational Approaches for Valorization of Biomass in the DES System

### 9.1 Density Functional Theory (DFT) and Molecular Dynamics (MD)

Physics-based computational approaches, including DFT and MD simulations, have been used to study biomass-solvent interactions, reaction mechanisms, and catalytic mechanisms of enzymes involved in biomass pretreatment and degradation at the molecular level. DFT is an effective tool for obtaining static properties, such as the reaction pathway and transition state, for a relatively small system.^386^ It has been used for assessment of thermophysical properties of solvents (e.g., diffusivity, viscosity, and conductivity),^387^ bond dissociation enthalpies of different lignin interunit linkages,^388^ and number of hydrogen bonds between biomass components and pretreatment solvents.^389^ For larger systems containing millions of atoms, MD is essential for assessing the equilibrium thermodynamic and physical properties, as well as predicting molecular interaction mechanisms. For instance, the viscosity of DES, a key physical property that governs biomass pretreatment efficiency by controlling mass/heat transfer and biomass accessibility, can be effectively assessed through MD simulations. It has been shown that an increase in the number of OH groups in the HBD component promotes the formation of a more extensive hydrogen-bond network, resulting in a higher viscosity of the DES.^390^ While classical MD simulations mainly focus on physical and elastic collisions between atoms and may not provide information about quantum chemistries such as bond cleavage and intermolecular recombination behavior, empirical force fields such as GROningen Molecular Simulation (GROMOS) and Chemistry at Harvard Macromolecular Mechanics (CHARMM) have been used in MD simulations to study nonbonded interactions, such as van der Waals and Coulombic interactions between biomass, water, and pretreatment solvent.^391^

At present, the study of biomass behavior in DES pretreatment systems via computational tools is still in its initial stage and mainly focuses on delignification and carbohydrate dissolution mechanisms. The important role of hydrogen bonding interactions in lignin and cellulose dissolution in DES has been highlighted in several studies.^392,393^ The characteristics of the hydrogen-bonding network and the strength of the interaction depend on the type of functional group in lignin (e.g., carboxyl and hydroxyl groups) and the number of oxygen atoms in the HBD of a DES system. One of the most commonly used quantitative description parameters of hydrogen bond donating and accepting ability (also known as acidity and basicity) are given by Kamlet–Taft solvatochromic parameters, denoted by α, β, and π* for hydrogen bond acidity, basicity, and dipolarity of the solvent, respectively). Kamlet–Taft solvatochromic parameters in combination with DFT calculations are often employed to study lignin solubilization mechanism in DES from the view of molecular interactions.^394^ A recent study indicated that DES with larger values of hydrogen bond basicity was capable of dissolving carbohydrate efficiently, while solvents with larger net basicity (β–α) are favorable for lignin solubilization.^170^ Besides being determined experimentally utilizing the Reichardt’s dyes or NMR,^395^ which is often impractical given the high cost of the dyes and virtually unlimited combinations of HBAs and HBDs in designed DESs, their solvatochromic parameters can also be predicted using computational approaches, such as machine learning algorithms trained on quantum chemically derived input features.^396^ Li et al.^397^ developed a trifunctional choline chloride/glyoxylic acid based binary DES for efficient fractionation of light-colored lignin with near proto-structures and theoretical yield of aromatic monomer upon depolymerization.^397^ DFT suggested that synergetic interaction between glyoxylic acid and choline chloride promoted the acetal reaction by reducing its Gibbs free energy, which subsequently stabilized the vulnerable β–O–4 interunit linkages and shielded the hydroxyl groups. DFT and MD simulations are often integrated to elucidate the interaction mechanisms of DES with lignin substrates. The MD simulations revealed the solvation effects of DES molecules on the reactivity of aryl ether linkages, including the formation of a charged solvation shell, variations in ether linkage exposure, and adjustments in the electrostatic potential around the linkages.^398^ In a typical choline chloride based DES system, the interaction between lignin and Cl^–^ was mainly electrostatic interaction, while the interaction between lignin and Ch^+^ was mainly van der Waals interactions.^399^ The types of functional group in lignin and the HBD also influence simulations the characteristics of the hydrogen-bonding network, as it has been revealed that carboxylic acids, such as lactic acid, tend to form hydrogen bonds with the γ-OH of lignin, whereas alcohols such as ethylene glycol prefer α-OH in lignin.^400^ Recent DFT and MD simulation studies have also highlighted the advances in ternary DES systems for lignin fractionation. In a ternary DES system containing ChCl, propylene glycol, and p-toluenesulfonic acid (PTSA), Cl^–^ was found to play a critical role in lignin depolymerization by forming hydrogen bonds with the γ-OH of lignin.^393^ MD simulation revealed that the addition of PTSA into the original DES system provided extra protons, formed new π–π stacking interactions with the lignin aromatic ring, and destroyed the old π–π stacking interaction between lignin molecules. Similarly, addition of p-hydroxybenzoic acid into a traditional ChCl-based DES also increased the hydrogen bonds and van der Waals interactions between DES and lignin system.^401^ Xia et al.^256^ developed a three-constituent DES system by coordinating AlCl_3_ in a traditional ChCl/glycerol DES system, and the lignin fractionation efficiency significantly improved from 3.6 to 95.5%. DFT and quantum mechanics calculations suggested that AlCl_3_ as an anion donor and acidic site holder could provide anionic hydrogen bond competition, and that the unidentate Al ligands were synchronized to form supramolecular chlorine–metal cation–HBA complexes, which allowed the multisite bridging ligands to break the aryl ether bonds in lignin (Figure 11).^256^ Other factors that may affect DES pretreatment efficiency include hydrophilic ability, solvent polarity, and acidity of the HBD, as revealed by DFT calculations.^283^ It was also reported that lignin under an electric field or microwave radiation could lengthen its bonds and increase the probability of breaking in DES, as revealed by MD simulations.^301^ MD simulations using the field of ReaxFF were utilized to propose the delignification mechanism of a phenyl glycoside LCC model in a choline-based DES system.^402^ The results showed that free protons in DES were important factors governing the delignification process, and acidic HBD generally resulted in higher delignification efficiency. Carbohydrate solubility in the DES system as well as their interaction with DES were also simulated by MD, and it was indicated that the anion of HBA and the hydroxyl groups from HBD molecules interacted mainly with cellulose hydroxyl groups, and thus were primarily responsible for its dissolution and deconstruction in the TBAB-based DES system.^403^ DFT-quantum modeling also suggested that cellulose dissolution in DES was mainly driven by strong H-bond interactions, and the attractive forces between cellulose and HBD were significantly stronger than those between cellulose and HBA.^404^ Similar to cellulose, MD simulations revealed that the solubilization mechanisms of hemicellulose in the DES system were also based on the strong hydrogen bond between the anion of HBD and the hydroxyl group of xylan.^405^ A recent study further showed that the removal of hemicellulose during DES pretreatment exposed lignin to the pretreatment solvent, which leads to lignin expansion according to molecular dynamics simulations.^406^

**Figure 11:**
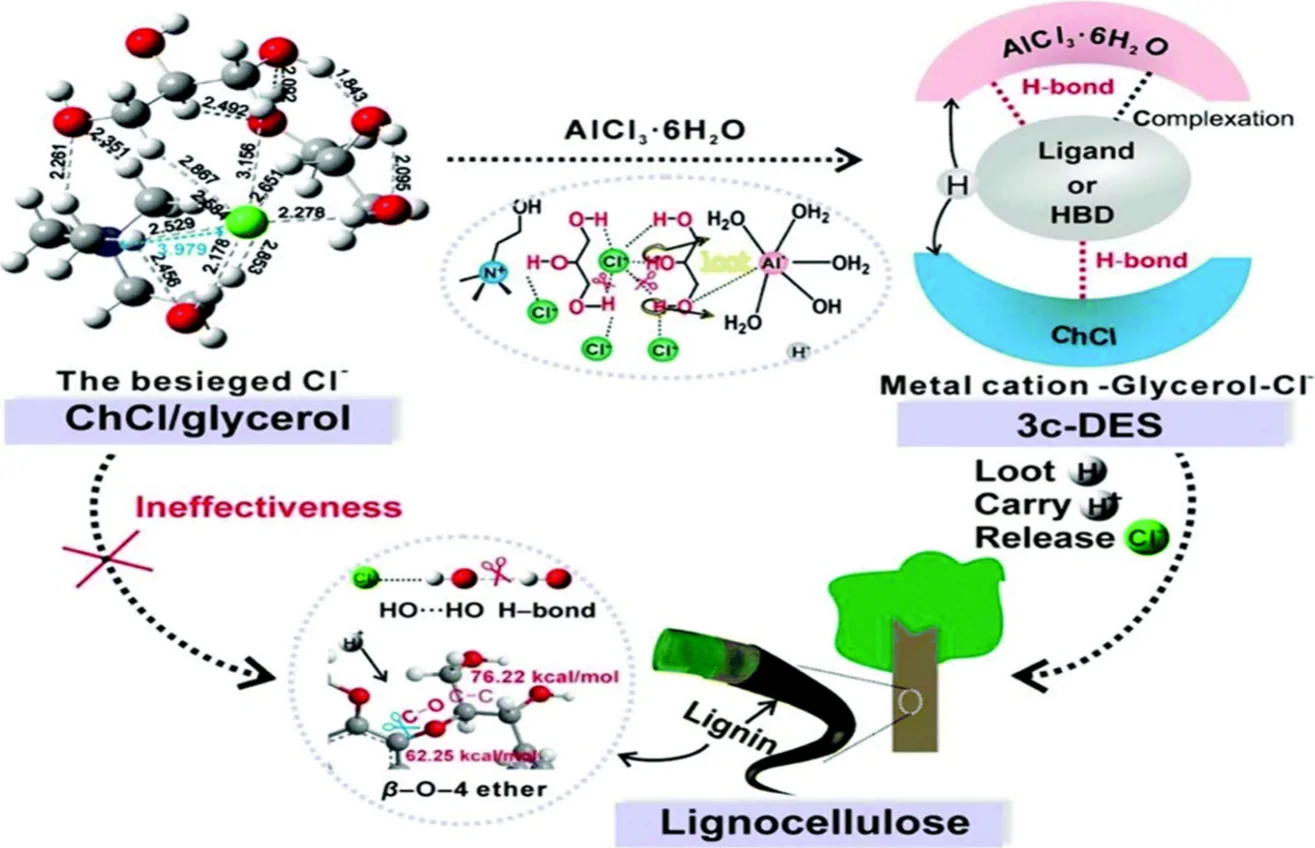
Illustration of the design of the ternary ChCl/glycerol/AlCl3 DES pretreatment solvent system.^256^ Reproduced from ref 256 with permission from the Royal Society of Chemistry. Copyright 2018 The Royal Society of Chemistry.

### 9.2 Conductor-like Screening Model for Real Solvents (COSMO-RS)

COSMO-RS, is a quantum chemistry-based thermodynamic model that predicts solubility and activity coefficients of solutes (such as lignin) in solvents like DES and ionic liquids without experimental input. COSMO-RS has been used to predict liquid–liquid equilibrium in DES system,^407^ predict solvatochromic (e.g., Kamlet–Taft) parameters for DES,^396,408^ screen DES as green extractants for the extraction of oil and antioxidants from plants,^409,410^ CO_2_ capture,^411^ bioethanol separation from water,^412^ and removal of contaminates from aqueous media.^413^ It is particularly valuable in predicting solubility trends for lignin model compounds, screening DES components to identify those with favorable interactions with lignin, and finally optimizing DES compositions to maximize lignin dissolution while minimizing degradation of carbohydrates in those DES systems. For example, lignin solubility in solvents like ionic liquid or DES could be assessed by calculating the thermodynamic properties of the logarithmic activity coefficient at infinite dilution, and lower values are beneficial as emphasized by several studies^414–416^ A recent study presented an integrated COSMO-RS and experimental approach to screen and design DES that selectively tune lignin structures during biomass DES pretreatment.^417^ Over 260 DES combinations comprising 13 HBAs and 20 HBDs were screened against eight lignin model compounds including various dimers and lignin-carbohydrate complexes to predict the activity coefficients to assess the DESs’ effectiveness in disrupting aryl ether linkages for lignin depolymerization (Figure 12). By combing computational screening, experimental validation, and multivariate analysis, this research establishes a framework for designing DESs that can be tailored to produce lignin with specific structural features, thereby expanding its applicability in high-value biomaterials and improving the economic and environmental sustainability of lignocellulosic biorefineries. One of the drawbacks of COSMO-RS is unlike MD simulations, this tool does not readily provide information on specific molecular interactions (e.g., hydrogen bonding) driving solution. DFT and MD simulation provide insights into hydrogen-bonding networks, solvent–lignin interactions and delignification mechanisms, revealing how DES composition, acidity, polarity, and additives influence the solubilization of biomass components while COSMO-RS complements these methods by predicting solubility in DES, enabling high-throughput screening and rational design of DES formulations to maximize solubilization and fractionation. Together, these methods establish a framework for designing effective DES systems for biomass processing.

**Figure 12:**
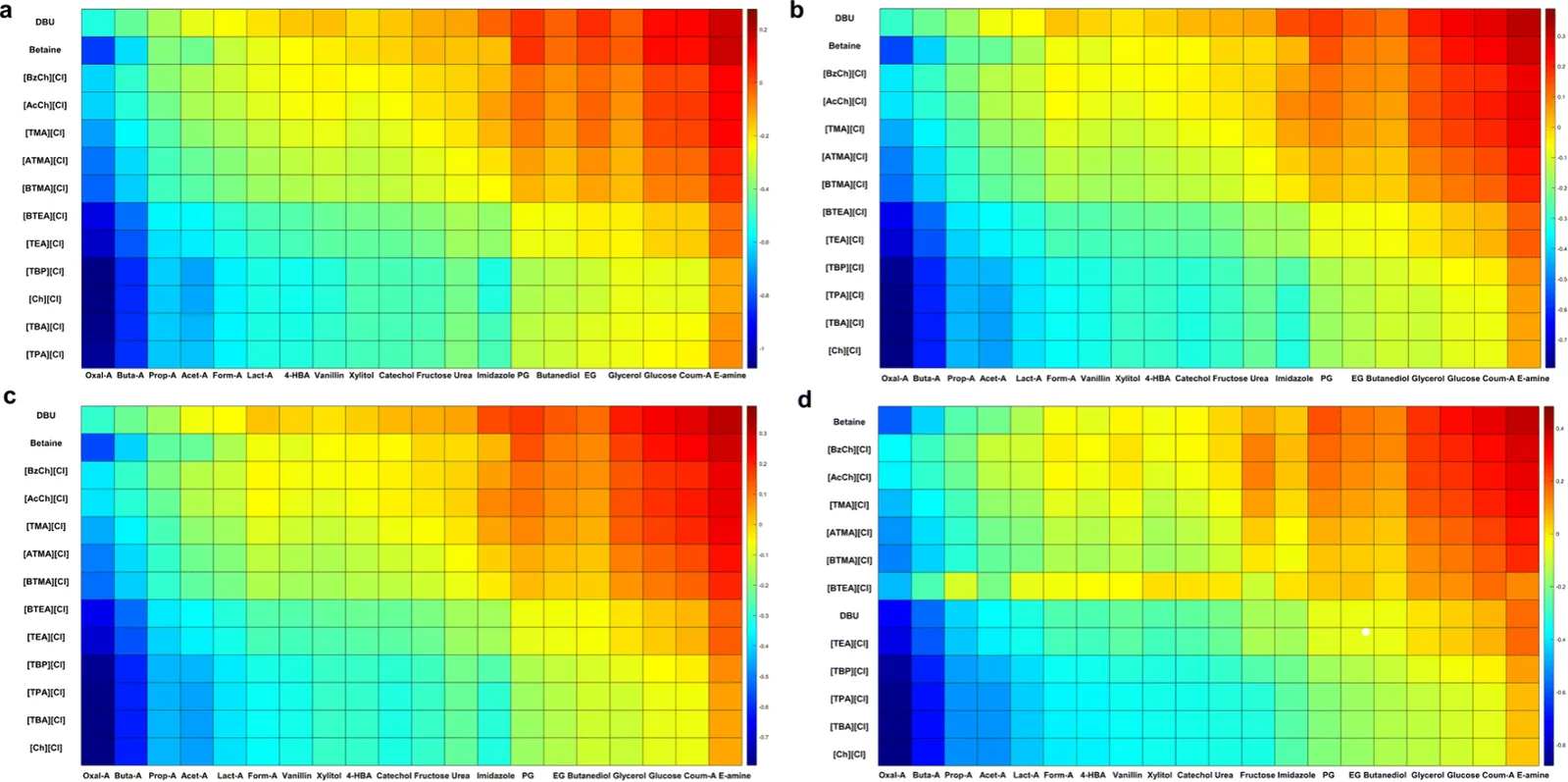
Logarithmic activity coefficient (ln γ) prediction of the lignin model in 260 DESs at 393.15 K. (a) HG-lignin model, (b) SS-lignin model, (c) GG-lignin model, and (d) SG-lignin model.^417^ Reproduced from ref 417 with permission from the Royal Society of Chemistry. Copyright 2025 The Royal Society of Chemistry.

## 10 Challenges and Opportunities

Following the overexploitation of fossil fuels and the global energy crisis, DESs are one of the many pretreatment methods introduced to implement sustainable biorefinery processes and products from lignocellulosic biomass. Although there has been a significant surge in research on this subject, research on DES synthesis and its industrial application, including biomass processing, is still in its infancy. Depending on their specific constituents, DESs offer promising “green” properties for biomass fractionation and valorization that warrant the replacement of fossil fuels with biofuels. Some of these are the ease of synthesis, low cost, nontoxicity, and biodegradability. Despite recent advancements in this field, there are still issues concerning the fundamental mechanisms and technical challenges in using DESs that require further investigation for future work.

DESs are excellent for lignin solubilization from biomass, but not cellulose and hemicellulose. The latter two residues remain intact for further processing by enzymatic hydrolysis. Further investigation is needed to determine the impact of DES composition and physicochemical properties on lignin, cellulose, and hemicellulose. To achieve this, pretreatment conditions, such as the HBA/HBD molar ratio, pretreatment temperature and time, viscosity, and other process control variables based on the feedstock nature, should also be extensively examined. The development of standardized methods to assess properties like hydrophobicity remains necessary. Notably, the pretreatment temperature is limited by the thermal stability of the DES. Existing research has shown that DESs with low viscosity and good thermal stability are the most efficient for biomass processing; therefore, further research is required to synthesize DESs of this nature. DESs generally have a high viscosity, but their relevance to lignocellulosic biomass is not well understood. To reduce DES viscosity, water can be added as a cosolvent. This suggests that less solvent is required, thereby reducing the operational costs of the process. This reflects scalability issues in industrial processes. However, the intermolecular bonding structure of the solvent may be disrupted if it is added disproportionately. More insight into the water/DES ratio and intermolecular interactions is required in the DES molecular framework. Despite its extensive study and properties that merit its use and applications, ChCl presents challenges such as thermal degradation and strong odor and therefore, alterative HBAs should be identified.

Numerous studies have recognized DESs as nontoxic and environmentally friendly. However, there are few reports that say otherwise on the toxicity and biocompatibility of DESs, which will need further investigation. Hayyan et al.^418^ studied the toxicity and cytotoxicity of different DESs on microorganisms and discovered that ChCl-based DESs showed no toxic effects on the bacterial species under study. However, their study suggested that the DESs had higher cytotoxicity levels than HBA and HBD. In another study, Wen et al.^419^ refutably reported that ChCl-based DESs with HBDs such as glycerol, ethylene glycol, urea, and acetamide have antibacterial properties. Insights into DES toxicity levels and biocompatibility with living systems are essential for large-scale industrial applications. An area that remains relatively underexplored is the long-term stability of DESs, particularly concerning the interactions between their components and their behavior during their extended use.^420^ In addition to chemical reactivity, the potential for solid reformation within the solvent overtime warrants investigation.^420^ From an industrial standpoint, properties such as corrosion are critically important and warrant further investigation,^420^ particularly because DESs are intended to replace highly corrosive mineral acids. Experimental evidence suggests that DESs are relatively easy to recycle and maintain their enzymatic activity, but high reuses need be performed to further prove this, as some studies have proved this to be untrue. Research has shown the advantages and effectiveness of integrating pretreatment methods, and a comprehensive understanding of this subject will be helpful in advancing biomass processing. Furthermore, techno-economic analysis and life cycle analysis of biomass processing using DESs should be conducted, and the study of their integration should be evaluated, as well as the current limitations of TEA and LCA. Thus, DESs can be manufactured at an industrial scale as low-cost green solvents. Sufficient findings have proved that DESs are potential alternatives to conventional solvents for valorizing lignocellulosic biomass.

## 11 Comparative Scope and Novelty of This Review

To highlight the thematic distinctiveness of this review relative to previous works, Figure 13 presents a conceptual Venn diagram demonstrating the thematic scope and novelty of this review compared to previous reviews on DES-based lignocellulosic biomass pretreatment. The overlapping section represents common areas of discussion, including the general background of lignocellulosic biomass, biomass pretreatment methods, DES fundamentals and their application in pretreating individual biomass components, biofuel production and sustainability assessments. Although some areas overlap with previous reviews, this work explores them in greater depth, offering a detailed and comprehensive analysis. While earlier literature has contributed significantly to establishing these foundations, it often provides broad overviews of DES properties without directly linking them to biomass pretreatment performance and this review narrows its focus to such physicochemical properties and discusses their influence on pretreatment efficiency. Notably, a novel inclusion is the discussion of DES thermal stability and its implications for DES recovery and recycling, emphasizing degradation as a key factor limiting feasibility of repeated use. A further distinction of this review is coverage of DES preparation methods, allowing readers to gain a comprehensive understanding of the diversity in DES preparation, including less-explored methods. Furthermore, this work examines DES performance on whole biomass fractionation, rather focusing solely on individual biomass components, offering an integrated perspective of DES efficiency in biomass processing. Additionally, it offers a comparative analysis of cellulose dissolution in DESs versus other known solvents (ILs and NMMO), addressing the known difficulty of cellulose dissolution by DESs.

**Figure 13:**
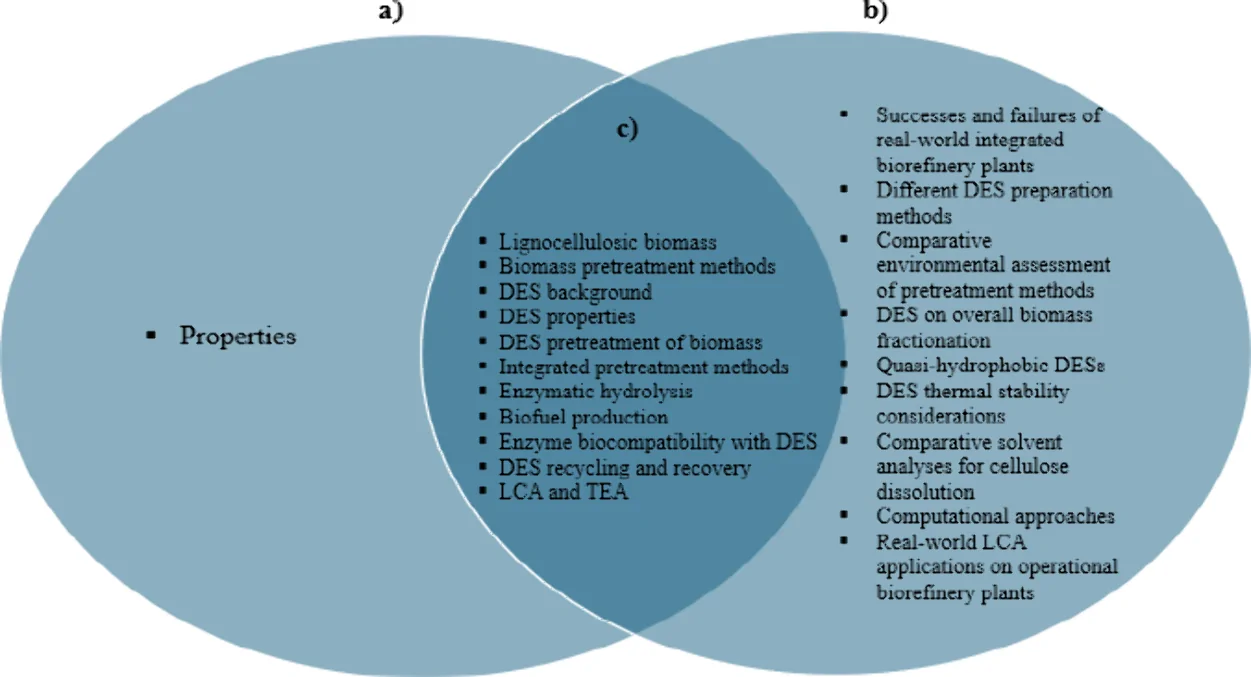
Conceptual Venn diagram illustrating the scope and novelty of this review relative to existing literature^124,202,421–426^ (a) and (c) the shared thematic areas whereas (b) is the gap, which is also addressed in the present review.

Distinctly, this review extends beyond previous scopes by incorporating computational approaches which are used to provide mechanistic insights and predictive capabilities for DES-based biomass pretreatment systems, bridging the gap between empirical observation and molecular-level understanding and supporting the rational design of DESs tailored for efficient lignocellulose pretreatment and fractionation. While many reviews cover laboratory-scale or pilot, few contextualize these finding within the realities of commercial deployment. This review discusses the successes and failures of operational biorefinery plants to provide a practical perspective on the technical, economic and logistical challenges that accompany transition from research to industrial implementation. Moreover, the review also discusses an LCA case study on an operational biorefinery plant to bridge laboratory findings with technical efficiency, scalability, environmental impact, long-term economic feasibility and offer practical guidance for future biorefinery development. This work further provides a practical perspective on the environmental implications of different biomass pretreatment methods by discussing a comparative environmental assessment of pretreatment methods, where key environmental metrics are used to evaluate methods based on sustainability indicators. Most importantly, this review draws on recent and up-to-date citations to ensure that it reflects the latest scientific and technological progress in DES chemistry, biomass processing and sustainability areas, emerging trends, challenges, and opportunities. This work extends beyond existing reviews by identifying knowledge gaps in areas such as DES recovery and recyclability, their interaction mechanism with lignocellulosic components, and the transition of laboratory-scale successes to industrial implementation. It also links physicochemical insights with environmental and techno-economic evaluations, therefore proposing integrative frameworks, bridging the gap between theoretical understanding and practical application, setting new directions for future DES research.

## 12 Conclusion

This review outlines the recent advancements in DES pretreatment for lignocellulosic biomass application, with factors that impact their efficacy, DES constituents, HBA/HBD molar ratio, pretreatment conditions (temperature, time, and integral pretreatment method), type of biomass, and the mechanisms thereof. Despite extensive studies on the subject, there are still discrepancies with respect to the ineffectuality of DES to disrupt the polysaccharide network, the reduction of viscosity of DES to enhance biomass processing, the synergic effect of integral pretreatment methods, recyclability of DESs, life cycle, and techno-economic assessments, all of which are important for developing a sustainable industrial bioprocess. For instance, acid-based DESs are efficient in biomass processing, but a techno-economic analysis must be conducted to evaluate the economic viability of biorefineries based on acid-based DES pretreatment. Furthermore, further comparative studies are needed to assess whether DESs offer consistent advantages over conventional solvents. In conclusion, computational approaches could provide guidance on the rational design of DES for biomass pretreatment and lignin fractionation and enable researchers to test DES pretreatment systems without the risk of expensive equipment and pressing processes that may not work as intended. Machine learning approaches that leverage experimental data sets could also become essential for identifying sustainable solvents and predicting biomass pretreatment efficiency. Using this review, researchers may perfect existing pretreatment methods or further investigate new approaches to valorize lignocellulosic biomass.

## References

[1] Brandt A., Gräsvik J., Hallett J. P., Welton T. (2013). Deconstruction of Lignocellulosic Biomass with Ionic Liquids. Green Chem..

[2] Kheshgi H. S., Prince R. C., Marland G. (2000). The Potential of Biomass Fuels in the Context of Global Climate Change: Focus on Transportation Fuels. Annu. Rev. Energy Environ..

[3] Huber G. W., Iborra S., Corma A. (2006). Synthesis of Transportation Fuels from Biomass: Chemistry, Catalysts, and Engineering. Chem. Rev..

[4] Tian D., Guo Y., Hu J., Yang G., Zhang J., Luo L., Xiao Y., Deng S., Deng O., Zhou W. (2020). Acidic Deep Eutectic Solvents Pretreatment for Selective Lignocellulosic Biomass Fractionation with Enhanced Cellulose Reactivity. Int. J. Biol. Macromol..

[5] Sankpal, S. T. ; Naikwade, P. V. Important bio-fuel crops: advantages and disadvantages. Int. J. Eng. Res. 2013, 4 (12).

[6] Valentine J., Clifton-Brown J., Hastings A., Robson P., Allison G., Smith P. (2012). Food vs. fuel: the use of land for lignocellulosic ‘next generation’energy crops that minimize competition with primary food production. Gcb Bioenergy.

[7] Limayem A., Ricke S. C. (2012). Lignocellulosic biomass for bioethanol production: current perspectives, potential issues and future prospects. Prog. Energy Combust. Sci..

[8] Cardona C. A., Sánchez Ó. J. (2007). Fuel ethanol production: process design trends and integration opportunities. Bioresour. Technol..

[9] Zabed H. M., Akter S., Yun J., Zhang G., Awad F. N., Qi X., Sahu J. (2019). Recent advances in biological pretreatment of microalgae and lignocellulosic biomass for biofuel production. Renew. Sustain. Energy Rev..

[10] Nanda S., Azargohar R., Dalai A. K., Kozinski J. (2015). An assessment on the sustainability of lignocellulosic biomass for biorefining. Renew. Sustain. Energy Rev..

[11] Sasmal, S. ; Mohanty, K. In Biorefining of Biomass to Biofuels: Opportunities and Perception; Kumar, S. , Sani, R. K. , Eds.; Springer International Publishing: Cham, 2018, 10.1007/978-3-319-67678-4_9.

[12] Akhtar N., Gupta K., Goyal D., Goyal A. (2016). Recent advances in pretreatment technologies for efficient hydrolysis of lignocellulosic biomass. Environ. Prog. Sustain. Energy.

[13] Dahadha S., Amin Z., Bazyar Lakeh A. A., Elbeshbishy E. (2017). Evaluation of different pretreatment processes of lignocellulosic biomass for enhanced biomethane production. Energ Fuels.

[14] Singhvi M. S., Gokhale D. V. (2019). Lignocellulosic biomass: Hurdles and challenges in its valorization. Appl. Environ. Microbiol..

[15] Zhao X., Zhang L., Liu D. (2012). Biomass recalcitrance. Part I: the chemical compositions and physical structures affecting the enzymatic hydrolysis of lignocellulose. Biofuels, Bioprod. Biorefin.

[16] Sannigrahi P., Hu F., Pu Y., Ragauskas A. (2012). A novel oxidative pretreatment of Loblolly Pine, Sweetgum, and Miscanthus by ozone. J. Wood Chem. Technol..

[17] Sharma V., Tsai M. L., Chen C. W., Sun P. P., Patel A. K., Singhania R. R., Nargotra P., Dong C. D. (2022). Deep eutectic solvents as promising pretreatment agents for sustainable lignocellulosic biorefineries: A review. Bioresour. Technol..

[18] Prasad B., Padhi R., Ghosh G. (2023). A review on key pretreatment approaches for lignocellulosic biomass to produce biofuel and value-added products. Int. J. Environ. Sci..

[19] Bajpai, P. ; Bajpai, P. Pretreatment of lignocellulosic biomass; Springer, 2016.

[20] Abbott A. P., Capper G., Davies D. L., Rasheed R. K., Tambyrajah V. (2003). Novel solvent properties of choline chloride/urea mixtures. Chem. Commun..

[21] Zhou M., Fakayode O. A., Yagoub A. E. A., Ji Q., Zhou C. (2022). Lignin fractionation from lignocellulosic biomass using deep eutectic solvents and its valorization. Renew. Sustain. Energy Rev..

[22] Xu G. C., Ding J. C., Han R. Z., Dong J. J., Ni Y. (2016). Enhancing cellulose accessibility of corn stover by deep eutectic solvent pretreatment for butanol fermentation. Bioresour. Technol..

[23] Gorke, J. T. ; Srienc, F. ; Kazlauskas, R. J. In Ionic liquid applications: pharmaceuticals, therapeutics, and biotechnology; ACS Publications, 2010.

[24] Baruah J., Nath B. K., Sharma R., Kumar S., Deka R. C., Baruah D. C., Kalita E. (2018). Recent trends in the pretreatment of lignocellulosic biomass for value-added products. Front. Energy Res..

[25] Klemm D., Heublein B., Fink H. P., Bohn A. (2005). Cellulose: Fascinating Biopolymer and Sustainable Raw Material. Angew. Chem., Int. Ed..

[26] Holtzapple, M. T. In Encyclopedia of Food Sciences and Nutrition (Second ed.); Caballero, B. , Ed.; Academic Press: Oxford, 2003, 10.1016/B0-12-227055-X/00185-1.

[27] Agbor V. B., Cicek N., Sparling R., Berlin A., Levin D. B. (2011). Biomass pretreatment: Fundamentals toward application. Biotechnol. Adv..

[28] Ling Z., Chen S., Zhang X., Takabe K., Xu F. (2017). Unraveling variations of crystalline cellulose induced by ionic liquid and their effects on enzymatic hydrolysis. Sci. Rep..

[29] Gupta P. K., Raghunath S. S., Prasanna D. V., Venkat P., Shree V., Chithananthan C., Choudhary S., Surender K., Geetha K. (2019). An update on overview of cellulose, its structure and applications. Cellul..

[30] Ratajczak K., Stobiecka M. (2020). High-performance modified cellulose paper-based biosensors for medical diagnostics and early cancer screening: A concise review. Carbohydr. Polym..

[31] Asif, M. In Sustainability of Construction Materials; Khatib, J. M. , Ed.; Woodhead Publishing, 2009, 10.1533/9781845695842.31.

[32] Pecha, M. B. ; Garcia-Perez, M. In Bioenergy (Second ed.); Dahiya, A. , Ed.; Academic Press, 2020, 10.1016/B978-0-12-815497-7.00029-4.

[33] Langan P., Petridis L., O’Neill H. M., Pingali S. V., Foston M., Nishiyama Y., Schulz R., Lindner B., Hanson B. L., Harton S. (2014). Common processes drive the thermochemical pretreatment of lignocellulosic biomass. Green Chem..

[34] Soudham, V. P. Biochemical conversion of biomass to biofuels: pretreatment-detoxification-hydrolysis-fermentation. Umeå University, Göteborg, 2015.

[35] Chen, H. In Lignocellulose Biorefinery Engineering; Chen, H. , Ed.; Woodhead Publishing, 2015, 10.1016/B978-0-08-100135-6.00003-X.

[36] Bajpai, P. In Biermann’s Handbook of Pulp and Paper (Third ed.); Bajpai, P. , Ed.; Elsevier, 2018, 10.1016/B978-0-12-814240-0.00002-1.

[37] Saha B. C. (2003). Hemicellulose bioconversion. J. Ind. Microbiol. Biotechnol..

[38] Tryfona T., Bourdon M., Delgado Marques R., Busse-Wicher M., Vilaplana F., Stott K., Dupree P. (2023). Grass xylan structural variation suggests functional specialization and distinctive interaction with cellulose and lignin. Plant J..

[39] Luo Y., Li Z., Li X., Liu X., Fan J., Clark J. H., Hu C. (2019). The production of furfural directly from hemicellulose in lignocellulosic biomass: A review. Catal. Today.

[40] Wang Z., Zhou J., Yin Y., Mu M., Liu Y., Zhou D., Wang W., Zuo X., Yang J. (2024). A Novel Green Biorefinery Strategy for Corn Stover by Pretreatment with Weak Alkali-Assisted Deep Eutectic Solvents. Green Chem..

[41] Juturu V., Wu J. C. (2013). Biotechnology. Insight into microbial hemicellulases other than xylanases: a review. J. Chem. Technol. Biotechnol..

[42] Andlar M., Rezić T., Marđetko N., Kracher D., Ludwig R., Šantek B. (2018). Lignocellulose degradation: An overview of fungi and fungal enzymes involved in lignocellulose degradation. Eng. Life Sci..

[43] Khan, T. A. ; Lee, J.-H. ; Kim, H.-J. In Lignocellulose for Future Bioeconomy; Ariffin, H. , Sapuan, S. M. , Hassan, M. A. , Eds.; Elsevier, 2019, 10.1016/B978-0-12-816354-2.00009-8.

[44] Lee H. V., Hamid S. B. A., Zain S. K. (2014). Conversion of Lignocellulosic Biomass to Nanocellulose: Structure and Chemical Process. Sci. World J..

[45] Mariana M., Alfatah T., HPS A. K., Yahya E. B., Olaiya N. G., Nuryawan A., Mistar E. M., Abdullah C. K., Abdulmadjid S. N., Ismail H. (2021). A current advancement on the role of lignin as sustainable reinforcement material in biopolymeric blends. J. Mater. Sci. Technol..

[46] Selig M. J., Viamajala S., Decker S. R., Tucker M. P., Himmel M. E., Vinzant T. B. (2007). Deposition of lignin droplets produced during dilute acid pretreatment of maize stems retards enzymatic hydrolysis of cellulose. Biotechnol. Prog..

[47] Zitting A., Paajanen A., Altgen M., Rautkari L., Penttilä P. A. (2024). Role of Lignin in Moisture Interactions of Cellulose Microfibril Structures in Wood. Small Struct..

[48] Mistar E. M., Alfatah T., Supardan M. D. (2020). Synthesis and characterization of activated carbon from Bambusa vulgaris striata using two-step KOH activation. J. Mater. Sci. Technol..

[49] Feofilova E. P., Mysyakina I. S. (2016). Lignin: Chemical structure, biodegradation, and practical application (a review). Appl. Biochem. Biotechnol..

[50] Yoo C. G., Meng X., Pu Y., Ragauskas A. J. (2020). The critical role of lignin in lignocellulosic biomass conversion and recent pretreatment strategies: A comprehensive review. Bioresour. Technol..

[51] Xu Y. H., Li X. Y., Li M. F., Peng F., Ma J. F. (2021). Acetone fractionation of heterogeneous tetrahydrofurfuryl alcohol lignin to improve its homogeneity and functionality. J. Mater. Sci. Technol..

[52] Patil V., Adhikari S., Cross P., Jahromi H. (2020). Progress in the solvent depolymerization of lignin. Renew. Sustain. Energy Rev..

[53] Zakzeski J., Bruijnincx P. C., Jongerius A. L., Weckhuysen B. M. (2010). The catalytic valorization of lignin for the production of renewable chemicals. Chem. Rev..

[54] Szalaty T. J., Klapiszewski Ł., Jesionowski T. J. (2020). Recent developments in modification of lignin using ionic liquids for the fabrication of advanced materials-A review. J. Mol. Liq..

[55] Li X., Xu Y., Alorku K., Wang J., Ma L. (2023). A review of lignin-first reductive catalytic fractionation of lignocellulose. Mol. Catal..

[56] Jȩdrzejczak P., Collins M. N., Jesionowski T., Klapiszewski Ł. (2021). The role of lignin and lignin-based materials in sustainable construction - A comprehensive review. Int. J. Biol. Chem..

[57] Karak, N. In Biopolymers and Biotech Admixtures for Eco-Efficient Construction Materials; Pacheco-Torgal, F. , Ivanov, V. , Karak, N. , Jonkers, H. , Eds.; Woodhead Publishing, 2016, 10.1016/B978-0-08-100214-8.00015-4.

[58] Persson, B. Sulfitsprit: Förhoppningar och Besvikelser Under 100 År; DAUS Tryck & Media: Sweden, 2010.

[59] Rødsrud G., Lersch M., Sjöde A. (2012). History and Future of World’s Most Advanced Biorefinery in Operation. Biomass Bioenergy.

[60] Martin, J. Project Liberty: Launch of an Integrated Bio-Refinery with Eco-Sustainable and Renewable Technologies. Conversion of Corn Stover Biomass to Bio-Ethanol, Final Report; POET-DSM Project Liberty, LLC: Emmetsburg, IA, United States, 2021.

[61] Leon, G. Integrated Biorefinery for Conversion of Biomass to Ethanol, Synthesis Gas, and Heat; Abengoa Bioenergy: Hugoton, KS, United States, 2017.

[62] Froehlich A., Covalla S., Fromanger R., Hämäläinen J., Kaartinen T., Mastel B., Laine M. M. (2022). Multi-Sugar Fermentation of Lignocellulosic Hydrolysate under Industrially Relevant Conditions: Comparison of Several Yeast Strains. bioRxiv.

[63] De Blasio, C. In Fundamentals of Biofuels Engineering and Technology; Springer: Cham, 2019.

[64] St1. The Dismantling of ST1’s Bioethanol Production Plant Begins: Lessons Applied. St1. https://st1.com/article/the-dismantling-of-st1s-bioethanol-production-plant-begins-lessons-applied, accessed October 21, 2025.

[65] Hashemi, S. Optimization of Washing Steps Prior to Bleaching of a Pulp Produced by Cooking with Ionic Liquid. Thesis, Karlstad University, Faculty of Health, Science and Technology, Karlstad, 2024.

[66] Chundawat, S. ; Balan, V. ; Da Costa Sousa, L. ; Dale, B. In Bioalcohol production; Elsevier, 2010.

[67] Bensah E. C., Mensah M. (2013). Chemical Pretreatment Methods for the Production of Cellulosic Ethanol: Technologies and Innovations. Int. J. Chem. Eng..

[68] Behera S., Arora R., Nandhagopal N., Kumar S. (2014). Importance of chemical pretreatment for bioconversion of lignocellulosic biomass. Renew. Sustain. Energy Rev..

[69] Sun Y., Cheng J. (2002). Hydrolysis of lignocellulosic materials for ethanol production: a review. Bioresour. Technol..

[70] Haghighi Mood S., Hossein Golfeshan A., Tabatabaei M., Salehi Jouzani G., Najafi G. H., Gholami M., Ardjmand M. (2013). Lignocellulosic biomass to bioethanol, a comprehensive review with a focus on pretreatment. Renew. Sustain. Energy Rev..

[71] Aslanzadeh S., Ishola M. M., Richards T., Taherzadeh M. J. (2014). An overview of existing individual unit operations. Biorefin..

[72] Keskin, T. ; Nalakath Abubackar, H. ; Arslan, K. ; Azbar, N. In Biohydrogen (Second ed.); Pandey, A. , Mohan, S. V. , Chang, J. S. , Hallenbeck, P. C. , Larroche, C. , Eds.; Elsevier, 2019, 10.1016/B978-0-444-64203-5.00012-5.

[73] Zhu W., Zhu J., Gleisner R., Pan X. J. (2010). On energy consumption for size-reduction and yields from subsequent enzymatic saccharification of pretreated lodgepole pine. Bioresour. Technol..

[74] Chandra R. P., Bura R., Mabee W., Berlin d. A., Pan X., Saddler J. N. (2007). Substrate pretreatment: the key to effective enzymatic hydrolysis of lignocellulosics?. Biofuels.

[75] Hendriks A., Zeeman G. (2009). Pretreatments to enhance the digestibility of lignocellulosic biomass. Bioresour. Technol..

[76] Mtui G. Y. (2009). Recent advances in pretreatment of lignocellulosic wastes and production of value added products. Afr. J. Biotechnol..

[77] Wyman C. E., Dale B. E., Elander R. T., Holtzapple M., Ladisch M. R., Lee Y. J. (2005). Coordinated development of leading biomass pretreatment technologies. Bioresour. Technol..

[78] Aita, G. M. ; Kim, M. In Sustainability of the sugar and sugar-ethanol industries; ACS Publications, 2010.

[79] Klein-Marcuschamer D., Oleskowicz-Popiel P., Simmons B. A., Blanch H. W. (2010). Technoeconomic analysis of biofuels: A wiki-based platform for lignocellulosic biorefineries. Biomass Bioenerg..

[80] Toghiani J., Malekzadeh S., Jamali N., Afsham N., Fallah N., Mahboubi A., Nasernejad B., Taherzadeh M. J., Oladzad S. (2024). Novel Advanced Oxidation Processes (AOPs) as Lignocellulosic Biomass Pretreatment Approaches and Their Sustainability Assessment: A Review. Curr. Pollut. Rep..

[81] Wasserscheid, P. ; Welton, T. Ionic liquids in synthesis; John Wiley & Sons, 2008.

[82] Rothe M., Müller E., Denk P., Kunz W. (2021). Ionic Liquids Based on the Concept of Melting Point Lowering Due to Ethoxylation. Molecules.

[83] Reddy P. J. (2015). A critical review of ionic liquids for the pretreatment of lignocellulosic biomass. S. Afr. J. Sci..

[84] Molefi R., Matshetshe K., Kabane B., Xhakaza N. M., Deenadayalu N., Bahadur I. (2024). Pre-Screening of an Eco-Friendly Solvent for Separating Industrial Mixtures: A Useful Tool for Solvent Selection. Fluid Phase Equilib..

[85] Lynam J. G., Kumar N., Wong M. J. (2017). Deep eutectic solvents’ ability to solubilize lignin, cellulose, and hemicellulose; thermal stability; and density. Bioresour. Technol..

[86] Chen Y., Mu T. (2019). Application of deep eutectic solvents in biomass pretreatment and conversion. Green Energy & Env..

[87] Abbott A. P., Boothby D., Capper G., Davies D. L., Rasheed R. K. (2004). Deep eutectic solvents formed between choline chloride and carboxylic acids: versatile alternatives to ionic liquids. J. Am. Chem. Soc..

[88] Liu L., Li Q., Wan C. (2025). Deep Eutectic Solvent-Based Microextraction System for Simultaneous Lignocellulose Fractionation and Furfural Production. Green Chem..

[89] Smith E. L., Abbott A. P., Ryder K. S. (2014). Deep Eutectic Solvents (DESs) and Their Applications. Chem. Rev..

[90] Abbott A. P., Harris R. C., Ryder K. S. (2007). Application of hole theory to define ionic liquids by their transport properties. J. Phys. Chem. B.

[91] Abranches D. O., Martins M. A., Silva L. P., Schaeffer N., Pinho S. P., Coutinho J. A. (2019). Phenolic hydrogen bond donors in the formation of non-ionic deep eutectic solvents: the quest for type V DES. Chem. Commun..

[92] Tang X., Zuo M., Li Z., Liu H., Xiong C., Zeng X., Sun Y., Hu L., Liu S., Lei T. (2017). Green processing of lignocellulosic biomass and its derivatives in deep eutectic solvents. ChemSusChem.

[93] EFSA Panel on Additives and Products or Substances used in Animal Feed (FEEDAP) . Scientific Opinion on safety and efficacy of choline chloride as a feed additive for all animal species. EFSA J. 2011, 9 (9), 2353.

[94] Dai Y., van Spronsen J., Witkamp G.-J., Verpoorte R., Choi Y. H. (2013). Natural Deep Eutectic Solvents as New Potential Media for Green Technology. Anal. Chim. Acta.

[95] Florindo C., Branco L. C., Marrucho I. M. (2019). Quest for Green-Solvent Design: From Hydrophilic to Hydrophobic (Deep) Eutectic Solvents. ChemSusChem.

[96] Duarte A. R. C., Ferreira A. S. D., Barreiros S., Cabrita E., Reis R. L., Paiva A. (2017). A Comparison Between Pure Active Pharmaceutical Ingredients and Therapeutic Deep Eutectic Solvents: Solubility and Permeability Studies. Eur. J. Pharm. Biopharm..

[97] Chandran D., Khalid M., Walvekar R., Mubarak N. M., Dharaskar S., Wong W. Y., Gupta T. C. S. (2019). Deep Eutectic Solvents for Extraction-Desulphurization: A Review. J. Mol. Liq..

[98] Palmelund H., Rantanen J., Löbmann K. (2021). Deliquescence Behavior of Deep Eutectic Solvents. Appl. Sci..

[99] Jablonský M., Škulcová A., Ház A., Šima J., Majová V. (2018). Long-Term Isothermal Stability of Deep Eutectic Solvents. BioResources..

[100] Gambino M., Gaune P., Nabavian M., Gaune-Escard M., Bros J. (1987). Enthalpie de Fusion de l’Uree et de Quelques Melanges Eutectiques à Base d’Uree. Thermochim. Acta.

[101] Abbott A. P., Barron J. C., Ryder K. S., Wilson D. (2007). Eutectic-based ionic liquids with metal-containing anions and cations. Chem. - Eur. J..

[102] Abranches D. O., Martins M. A., Silva L. P., Schaeffer N., Pinho S. P., Coutinho J. A. J. C. C. (2019). Phenolic hydrogen bond donors in the formation of non-ionic deep eutectic solvents: the quest for type V DES. Chem. Commun..

[103] van Osch D. J., Zubeir L. F., van den Bruinhorst A., Rocha M. A., Kroon M. C. (2015). Hydrophobic deep eutectic solvents as water-immiscible extractants. Green Chem..

[104] Schaeffer N., Abranches D. O., Silva L. P., Martins M. A., Carvalho P. J., Russina O., Triolo A., Paccou L., Guinet Y., Hedoux A., Coutinho J. A. (2021). Non-ideality in thymol+ menthol type V deep eutectic solvents. ACS Sustain. Chem. Eng..

[105] Ijardar S. P., Singh V., Gardas R. L. (2022). Revisiting the physicochemical properties and applications of deep eutectic solvents. Molecules.

[106] Choi Y. H., van Spronsen J., Dai Y., Verberne M., Hollmann F., Arends I. W., Witkamp G. J., Verpoorte R. J. (2011). Are natural deep eutectic solvents the missing link in understanding cellular metabolism and physiology?. Plant Physiol..

[107] Socas-Rodríguez B., Torres-Cornejo M. V., Álvarez-Rivera G., Mendiola J. A. (2021). Deep eutectic solvents for the extraction of bioactive compounds from natural sources and agricultural by-products. Appl. Sci..

[108] González C. G., Mustafa N. R., Wilson E. G., Verpoorte R., Choi Y. H. (2018). Application of natural deep eutectic solvents for the “green” extraction of vanillin from vanilla pods. Flavour Fragr. J..

[109] Muradymov R., Paul N., Das N. K., Banerjee T., Shishov A. (2024). Quasi-hydrophobic deep eutectic solvents for simultaneous automated determination of polar and non-polar dyes in food products. Microchem. J..

[110] Rageh A. H., Abdel-Aal F. A. M., Farrag S. A., Ali A. B. H. (2024). A surfactant-based quasi-hydrophobic deep eutectic solvent for dispersive liquid-liquid microextraction of gliflozins from environmental water samples using UHPLC/fluorescence detection. Talanta.

[111] Shishov A., Dubrovsky I., Kirichenko S., Bulatov A. (2022). Behavior of quaternary ammonium salts and terpenoids-based deep eutectic solvents in aqueous phase. J. Mol. Liq..

[112] Soylak M., Uzcan F., Goktas O. (2023). Ultrasound-assisted quasi-hydrophobic deep eutectic solvent-based determination of trace Rhodamine B in water and food samples: A simple and green approach. J. Food Compos. Anal..

[113] Gissawong N., Mukdasai S., Boonchiangma S., Sansuk S., Srijaranai S. (2020). A rapid and simple method for the removal of dyes and organophosphorus pesticides from water and soil samples using deep eutectic solvent embedded sponge. Chemosphere.

[114] El-Deen A. K., Shimizu K. (2025). QbD-integrated natural eutectic solvent microextraction for simultaneous determination of co-administered antithrombotic drugs: A holistic evaluation of greenness, applicability, innovation, and performance. Microchem. J..

[115] Abdelwahed F. T., Mortada W. I., Eltabey R. M. (2025). A new surfactant-based hydrophobic deep eutectic solvent for microextraction and spectrophotometric determination of Rhodamine B and Allura red in real samples. Microchem. J..

[116] Shishov A., Timofeeva I., Gerasimov A., Israelyan D., Bulatov A. (2024). A hydrophobic deep eutectic solvent-based microextraction for the determination of ultra-trace arsenic in foods by an electrothermal atomization atomic absorption spectrometry. Talanta.

[117] Kivelä H., Salomäki M., Vainikka P., Mäkilä E., Poletti F., Ruggeri S., Terzi F., Lukkari J. (2022). Effect of Water on a Hydrophobic Deep Eutectic Solvent. J. Phys. Chem. B.

[118] El Achkar T., Greige-Gerges H., Fourmentin S. (2021). Basics and properties of deep eutectic solvents: a review. Environ. Chem. Lett..

[119] Tang B., Zhang H., Row K. H. (2015). Application of deep eutectic solvents in the extraction and separation of target compounds from various samples. J. Sep. Sci..

[120] Florindo C., Oliveira F. S., Rebelo L. P. N., Fernandes A. M., Marrucho I. M. (2014). Insights into the synthesis and properties of deep eutectic solvents based on cholinium chloride and carboxylic acids. ACS Sustain. Chem. Eng..

[121] Gutierrez M. C., Ferrer M. L., Mateo C. R., del Monte F. (2009). Freeze-drying of aqueous solutions of deep eutectic solvents: a suitable approach to deep eutectic suspensions of self-assembled structures. Langmuir.

[122] Gomez F. J., Espino M., Fernández M. A., Silva M. F. (2018). A greener approach to prepare natural deep eutectic solvents. ChemistrySelect.

[123] Santana A. P., Mora-Vargas J. A., Guimaraes T. G., Amaral C. D., Oliveira A., Gonzalez M. H. (2019). Sustainable synthesis of natural deep eutectic solvents (NADES) by different methods. J. Mol. Liq..

[124] Satlewal A., Agrawal R., Bhagia S., Sangoro J., Ragauskas A. (2018). Natural deep eutectic solvents for lignocellulosic biomass pretreatment: Recent developments, challenges and novel opportunities. Biotechnol. Adv..

[125] Marchel M., Cieśliński H., Boczkaj G. (2022). Thermal Instability of Choline Chloride-Based Deep Eutectic Solvents and Its Influence on Their Toxicity—Important Limitations of DESs as Sustainable Materials. Ind. Eng. Chem. Res..

[126] Yiin C. L., Yap K. L., Ku A. Z. E., Chin B. L. F., Lock S. S. M., Cheah K. W., Loy A. C. M., Chan Y. H. (2021). Recent advances in green solvents for lignocellulosic biomass pretreatment: potential of choline chloride (ChCl) based solvents. Bioresour. Technol..

[127] Farooq M. Q., Abbasi N. M., Anderson J. L. (2020). Deep eutectic solvents in separations: Methods of preparation, polarity, and applications in extractions and capillary electrochromatography. J. Chromatogr. A.

[128] Pollet P., Davey E. A., Ureña-Benavides E. E., Eckert C. A., Liotta C. L. (2014). Solvents for sustainable chemical processes. Green Chem..

[129] Hansen B. B., Spittle S., Chen B., Poe D., Zhang Y., Klein J. M., Horton A., Adhikari L., Zelovich T., Doherty B. W. J. C. r. (2021). Deep eutectic solvents: A review of fundamentals and applications. Chem. Rev..

[130] Ijardar S. P. (2020). Deep eutectic solvents composed of tetrabutylammonium bromide and PEG: Density, speed of sound and viscosity as a function of temperature. J. Chem. Thermodyn..

[131] Sazali A. L., Amran S. K., Pa K., Anuar M. R., Kelly Yong T. J. M. J. A. S. (2023). Potential of lignin from oil palm biomass using deep eutectic solvents as carbon fibre precursor. Malays. J. Anal. Sci..

[132] Shahbaz K., Mjalli F., Hashim M., AlNashef I. (2011). Using deep eutectic solvents based on methyl triphenyl phosphunium bromide for the removal of glycerol from palm-oil-based biodiesel. Energ Fuels.

[133] Chen W., Xue Z., Wang J., Jiang J., Zhao X., Mu T. (2018). Investigation on the thermal stability of deep eutectic solvents. Acta Phys. Chim. Sin.

[134] Liu S., Yu D., Chen Y., Shi R., Zhou F., Mu T. (2022). High-Resolution Thermogravimetric Analysis Is Required for Evaluating the Thermal Stability of Deep Eutectic Solvents. Ind. Eng. Chem. Res..

[135] Li Y., Li Y., Li H., Fan X., Yan H., Cai M., Xu X., Zhu M. (2023). Insights into the tribological behavior of choline chloride—urea and choline chloride—thiourea deep eutectic solvents. Friction.

[136] Wang H., Jing Y., Wang X., Yao Y., Jia Y. (2011). Ionic liquid analogous formed from magnesium chloride hexahydrate and its physico-chemical properties. J. Mol. Liq..

[137] Kareem M. A., Mjalli F. S., Hashim M. A., AlNashef I. M. (2010). Phosphonium-Based Ionic Liquids Analogues and Their Physical Properties. J. Chem. Eng. Data.

[138] Sun T., Teja A. S. (2003). Density, viscosity, and thermal conductivity of aqueous ethylene, diethylene, and triethylene glycol mixtures between 290 and 450 K. J. Chem. Eng. Data.

[139] Abbott A. P., Harris R. C., Ryder K. S., D’Agostino C., Gladden L. F., Mantle M. D. (2011). Glycerol eutectics as sustainable solvent systems. Green Chem..

[140] Frimpong, M. Understanding the Role of HBA/HBD Variations on Deep Eutectic Solvent Properties and Activity Using Acetylsalicylic Acid as a Model Drug. Dissertation, South Dakota State University, 2023.

[141] Sarmad S., Xie Y., Mikkola J. P., Ji X. (2017). Screening of deep eutectic solvents (DESs) as green CO 2 sorbents: from solubility to viscosity. New J. Chem..

[142] Omar K. A., Sadeghi R. (2022). Physicochemical properties of deep eutectic solvents: A review. J. Mol. Liq..

[143] Chandran K., Kait C. F., Wilfred C. D., Zaid H. F. M. (2021). A review on deep eutectic solvents: Physiochemical properties and its application as an absorbent for sulfur dioxide. J. Mol. Liq..

[144] Florindo C., Oliveira M. M., Branco L. C., Marrucho I. M. (2017). Carbohydrates-based deep eutectic solvents: Thermophysical properties and rice straw dissolution. J. Mol. Liq..

[145] Hayyan A., Mjalli F. S., AlNashef I. M., Al-Wahaibi T., Al-Wahaibi Y. M., Hashim M. A. (2012). Fruit sugar-based deep eutectic solvents and their physical properties. Thermochim. Acta.

[146] Sánchez P. B., González B., Salgado J., JoséParajó J., Domínguez Á. (2019). Physical properties of seven deep eutectic solvents based on l-proline or betaine. J. Chem. Thermodyn.

[147] Abbott A. P., Capper G., Gray S. (2006). Design of improved deep eutectic solvents using hole theory. ChemPhysChem.

[148] Shi D., Zhou F., Mu W., Ling C., Mu T., Yu G., Li R. (2022). Deep insights into the viscosity of deep eutectic solvents by an XGBoost-based model plus SHapley Additive exPlanation. Phys. Chem. Chem. Phys..

[149] Lundberg, S. M. ; Lee, S.-I. A unified approach to interpreting model predictions. ArXiv 2017. 10.48550/arXiv.1705.07874

[150] AlOmar M. K., Hayyan M., Alsaadi M. A., Akib S., Hayyan A., Hashim M. A. (2016). Glycerol-based deep eutectic solvents: Physical properties. J. Mol. Liq..

[151] Omar K. A., Sadeghi R. (2021). Novel deep eutectic solvents based on pyrogallol: synthesis and characterizations. J. Chem. Eng..

[152] D’Agostino C., Harris R. C., Abbott A. P., Gladden L. F., Mantle M. D. (2011). Molecular motion and ion diffusion in choline chloride based deep eutectic solvents studied by 1 H pulsed field gradient NMR spectroscopy. Phys. Chem. Chem. Phys..

[153] Harifi-Mood A. R., Buchner R. (2017). Density, viscosity, and conductivity of choline chloride+ethylene glycol as a deep eutectic solvent and its binary mixtures with dimethyl sulfoxide. J. Mol. Liq..

[154] Zhu J., Yu K., Zhu Y., Zhu R., Ye F., Song N., Xu Y. (2017). Physicochemical properties of deep eutectic solvents formed by choline chloride and phenolic compounds at T = (293.15 to 333.15) K: The influence of electronic effect of substitution group. J. Mol. Liq..

[155] Abbott A. P., Capper G., Davies D. L., Rasheed R. (2004). Ionic liquids based upon metal halide/substituted quaternary ammonium salt mixtures. Inorg. Chem..

[156] Bahadori L., Chakrabarti M. H., Mjalli F. S., AlNashef I. M., Manan N. S. A., Hashim M. A. (2013). J Physicochemical properties of ammonium-based deep eutectic solvents and their electrochemical evaluation using organometallic reference redox systems. Electrochim. Acta.

[157] Mjalli F. S., Naser J. (2015). Viscosity model for choline chloride-based deep eutectic solvents. Asia-Pac. J. Chem. Eng..

[158] Guo W., Hou Y., Ren S., Tian S., Wu W. (2013). Formation of deep eutectic solvents by phenols and choline chloride and their physical properties. J. Chem. Eng. Data.

[159] Hou Y., Gu Y., Zhang S., Yang F., Ding H., Shan Y. (2008). Novel binary eutectic mixtures based on imidazole. J. Mol. Liq..

[160] Ruggeri S., Poletti F., Zanardi C., Pigani L., Zanfrognini B., Corsi E., Dossi N., Salomäki M., Kivelä H., Lukkari J. (2019). Chemical and electrochemical properties of a hydrophobic deep eutectic solvent. Electrochim. Acta.

[161] Zhu S., Li H., Zhu W., Jiang W., Wang C., Wu P., Zhang Q., Li H. (2016). Vibrational analysis and formation mechanism of typical deep eutectic solvents: An experimental and theoretical study. J. Mol. Graph. Model.

[162] Mota-Morales J. D., Gutiérrez M. C., Sanchez I. C., Luna-Bárcenas G., del Monte F. (2011). Frontal polymerizations carried out in deep-eutectic mixtures providing both the monomers and the polymerization medium. Chem. Commun..

[163] Reichardt C. (1994). Solvatochromic dyes as solvent polarity indicators. Chem. Rev..

[164] Pandey A., Rai R., Pal M., Pandey S. (2014). How polar are choline chloride-based deep eutectic solvents?. Phys. Chem. Chem. Phys..

[165] Kamlet M. J., Taft R. W. (1976). The solvatochromic comparison method. I. The. beta.-scale of solvent hydrogen-bond acceptor (HBA) basicities. J. Am. Chem. Soc..

[166] Valvi A., Dutta J., Tiwari S. (2017). Temperature-dependent empirical parameters for polarity in choline chloride based deep eutectic solvents. J. Phys. Chem. B.

[167] Zhang Q., De Oliveira Vigier K., Royer S., Jerome F. (2012). Deep eutectic solvents: syntheses, properties and applications. Chem. Soc. Rev..

[168] Florindo C., McIntosh A., Welton T., Branco L., Marrucho I. (2018). A closer look into deep eutectic solvents: exploring intermolecular interactions using solvatochromic probes. Phys. Chem. Chem. Phys..

[169] Dwamena A. K., Raynie D. E. (2020). Solvatochromic parameters of deep eutectic solvents: effect of different carboxylic acids as hydrogen bond donor. J. Chem. Eng. Data.

[170] Zhang L., Yu H., Liu S., Wang Y., Mu T., Xue Z. (2023). Kamlet-Taft parameters of deep eutectic solvents and their relationship with dissolution of main lignocellulosic components. Ind. Eng. Chem. Res..

[171] Kundu D., Rao P. S., Banerjee T. (2020). First-Principles Prediction of Kamlet-Taft Solvatochromic Parameters of Deep Eutectic Solvent Using the COSMO-RS Model. Ind. Eng. Chem. Res..

[172] Qin R., Wang Z., Wei C., Zhou F., Tian Y., Chen Y., Mu T. (2024). Quantification of alkalinity of deep eutectic solvents based on (H-) and NMR. Phys. Chem. Chem. Phys..

[173] Zhou F., Shi R., Wang Y., Xue Z., Zhang B., Mu T. (2022). Acidity scales of deep eutectic solvents based on IR and NMR. Phys. Chem. Chem. Phys..

[174] Pelaquim F. P., Barbosa Neto A. M., Dalmolin I. A. L., Costa M. C. (2021). Gas solubility using deep eutectic solvents: review and analysis. Ind. Eng. Chem. Res..

[175] Samin J. P., Heering A., Saame J., Kütt A., Leito I. (2025). Experimental unified pH scale in 1,2-dichloroethane. Phys. Chem. Chem. Phys..

[176] Marcus Y., Marcus Y. (2019). Properties of deep eutectic solvents. Deep eutectic solvents.

[177] Doneux T., Sorgho A., Soma F., Rayée Q., Bougouma M. (2024). Electrodeposition in Deep Eutectic Solvents: The ″Obvious″, the ″Unexpected″ and the ″Wonders″. Molecules.

[178] Abbott A. P., Alabdullah S. S., Al-Murshedi A. Y., Ryder K. S. (2018). Brønsted acidity in deep eutectic solvents and ionic liquids. Faraday Discuss..

[179] Panić M., Radović M., Cvjetko Bubalo M., Radošević K., Rogošić M., Coutinho J. A. P., RadojčićRedovniković I., Jurinjak Tušek A. (2022). Prediction of pH Value of Aqueous Acidic and Basic Deep Eutectic Solvent Using COSMO-RS σ Profiles’ Molecular Descriptors. Molecules.

[180] Skulcova A., Russ A., Jablonsky M., Sima J. (2018). The pH behavior of seventeen deep eutectic solvents. BioResources.

[181] Harmsen, P. ; Huijgen, W. ; Bermudez, L. ; Bakker, R. J. R. Literature review of physical and chemical pretreatment processes for lignocellulosic; Report number ECN-E—10-013; Energy Research Centre of The Netherlands: Petten, The Netherlands, 2010.

[182] Chaturvedi V., Verma P. (2013). An overview of key pretreatment processes employed for bioconversion of lignocellulosic biomass into biofuels and value added products. 3 Biotech.

[183] Brodeur G., Yau E., Badal K., Collier J., Ramachandran K. B., Ramakrishnan S. (2011). Chemical and physicochemical pretreatment of lignocellulosic biomass: a review. J. Enzyme Res..

[184] Kumar A. K., Parikh B. S., Pravakar M. (2016). Natural deep eutectic solvent mediated pretreatment of rice straw: bioanalytical characterization of lignin extract and enzymatic hydrolysis of pretreated biomass residue. Environ. Sci. Pollut Res..

[185] Procentese A., Raganati F., Olivieri G., Russo M. E., Rehmann L., Marzocchella A. J. (2018). Deep eutectic solvents pretreatment of agro-industrial food waste. Biotechnol Biofuels.

[186] del Mar Contreras-Gámez M., Galán-Martín Á., Seixas N., da Costa Lopes A. M., Silvestre A., Castro E. (2023). Deep eutectic solvents for improved biomass pretreatment: Current status and future prospective towards sustainable processes. Bioresour. Technol..

[187] Wikene K. O., Rukke H. V., Bruzell E., Tønnesen H. H. (2016). Physicochemical characterisation and antimicrobial phototoxicity of an anionic porphyrin in natural deep eutectic solvents. Eur. J. Pharm. Biopharm.

[188] Mjalli F. S., Ahmed O. U. (2016). Characteristics and intermolecular interaction of eutectic binary mixtures: Reline and Glyceline. Korean J. Chem. Eng..

[189] Naser J., Mjalli F., Jibril B., Al-Hatmi S., Gano Z. (2013). Potassium carbonate as a salt for deep eutectic solvents. Int. J. Chem. Eng. Appl..

[190] Kareem M. A., Mjalli F. S., Hashim M. A., AlNashef I. M. (2010). Phosphonium-based ionic liquids analogues and their physical properties. J. Chem. Eng..

[191] Zhao B.-Y., Xu P., Yang F. X., Wu H., Zong M. H., Lou W. Y. (2015). Biocompatible deep eutectic solvents based on choline chloride: characterization and application to the extraction of rutin from Sophora japonica. ACS Sustain Chem. Eng..

[192] Soares B., Tavares D. J. P., Amaral J. L., Silvestre A. J. D., Freire C. S. R., Coutinho J. A. P. (2017). Enhanced Solubility of Lignin Monomeric Model Compounds and Technical Lignins in Aqueous Solutions of Deep Eutectic Solvents. ACS Sustain. Chem. Eng..

[193] Devi M., Moral R., Thakuria S., Mitra A., Paul S. (2023). Hydrophobic Deep Eutectic Solvents as Greener Substitutes for Conventional Extraction Media: Examples and Techniques. ACS Omega.

[194] Omar K. A., Sadeghi R. (2022). Hydrophobic deep eutectic solvents: thermo-physical characteristics and their application in liquid-liquid extraction. J. Iran. Chem. Soc..

[195] Chen Y., Mu T. (2021). Revisiting greenness of ionic liquids and deep eutectic solvents. Green Chem. Eng..

[196] Bao Y., Wang Y., Yan C., Xue Z. (2025). Deep eutectic solvents for fractionation and valorization of lignocellulose. Green Chem. Eng..

[197] Tenhunen T.-M., Hakalahti M., Kouko J., Salminen A., Härkäsalmi T., Pere J., Harlin A., Hänninen T. (2015). Method for forming pulp fibre yarns developed by a design-driven process. Bioresour..

[198] Morais E. S., Lopes A. M. d. C., Freire M. G., Freire C. S. R., Coutinho J. A. P., Silvestre A. (2020). Use of Ionic Liquids and Deep Eutectic Solvents in Polysaccharides Dissolution and Extraction Processes towards Sustainable Biomass Valorization. Molecules.

[199] Ma, H. ; Fu, P. ; Zhao, J. ; Lin, X. ; Wu, W. ; Yu, Z. ; Xia, C. ; Wang, Q. ; Gao, M. ; Zhou, J. Pretreatment of Wheat Straw Lignocelluloses by Deep Eutectic Solvent for Lignin Extraction. Molecules 2022, 27 (22).7955 10.3390/molecules27227955 PMC9697946 36432056

[200] Hossain M. A., Rahaman M. S., Yelle D., Shang H., Sun Z., Renneckar S., Dong J., Tulaphol S., Sathitsuksanoh N. J. I. C. (2021). Products. Effects of polyol-based deep eutectic solvents on the efficiency of rice straw enzymatic hydrolysis. Ind. Crop. Prod..

[201] Hou X. D., Li A. L., Lin K. P., Wang Y. Y., Kuang Z. Y., Cao S. (2018). Insight into the structure-function relationships of deep eutectic solvents during rice straw pretreatment. Bioresour. Technol..

[202] Wang W., Lee D. J. (2021). Lignocellulosic biomass pretreatment by deep eutectic solvents on lignin extraction and saccharification enhancement: A review. Bioresour. Technol..

[203] Morán-Aguilar M. G., Calderón-Santoyo M., de Souza Oliveira R. P., Aguilar-Uscanga M. G., Domínguez J. M. (2022). Deconstructing sugarcane bagasse lignocellulose by acid-based deep eutectic solvents to enhance enzymatic digestibility. Carbohydr. Polym..

[204] Liang X., Zhu Y., Qi B., Li S., Luo J., Wan Y. (2021). Structure-property-performance relationships of lactic acid-based deep eutectic solvents with different hydrogen bond acceptors for corn stover pretreatment. Bioresour. Technol..

[205] Jablonsky M., Haz A., Majova V. (2019). Assessing the opportunities for applying deep eutectic solvents for fractionation of beech wood and wheat straw. Cellulose.

[206] Zhou J., Wu S., Liu Y. (2024). Structural changes of poplar lignin during the ternary deep eutectic solvent (DES) treatment and synergetic alkali-DES treatment. Ind. Crop. Prod..

[207] Deng H. Q., Lin X. H., Fan J. T., Fu P. Z., Guan J. J., Lei H. L., Liu L. H., Lai L. H., Hou X. D., Lou W. (2023). Glycolic acid-based deep eutectic solvents boosting co-production of xylo-oligomers and fermentable sugars from corncob and the related kinetic mechanism. Biotechnol. biofuels bioprod.

[208] Chen Z., Reznicek W. D., Wan C. J. (2018). Deep eutectic solvent pretreatment enabling full utilization of switchgrass. Bioresour. Technol..

[209] Tan Y. T., Chua A. S. M., Ngoh G. C. (2020). Deep eutectic solvent for lignocellulosic biomass fractionation and the subsequent conversion to bio-based products-A review. Bioresour. Technol..

[210] Xu H., Peng J., Kong Y., Liu Y., Su Z., Li B., Song X., Liu S., Tian W. (2020). Key process parameters for deep eutectic solvents pretreatment of lignocellulosic biomass materials: A review. Bioresour. Technol..

[211] Ai B., Li W., Woomer J., Li M., Pu Y., Sheng Z., Zheng L., Adedeji A., Ragauskas A. J., Shi J. (2020). Natural deep eutectic solvent mediated extrusion for continuous high-solid pretreatment of lignocellulosic biomass. Green Chem..

[212] Tenhunen T. M., Lewandowska A. E., Orelma H., Johansson L. S., Virtanen T., Harlin A., Österberg M., Eichhorn S. J., Tammelin T. (2018). Understanding the interactions of cellulose fibres and deep eutectic solvent of choline chloride and urea. Cellulose.

[213] Yang L., Xu L., Yang H., Shi Z., Zhao P., Yang J. (2022). Effect of different washing methods on reducing the inhibition of surface lignin in the tetraethylammonium chloride/oxalic acid-based deep eutectic solvent pretreatment. Ind. Crop. Prod..

[214] Olugbemide A. D., Oberlintner A., Novak U., Likozar B. (2021). Lignocellulosic Corn Stover Biomass Pre-Treatment by Deep Eutectic Solvents (DES) for Biomethane Production Process by Bioresource Anaerobic Digestion. Sustainability.

[215] Jia Z., Li X., Xu S., Sun Y., Chen L., Ye J., Li W., Wu T., Li J., Wang S. (2024). Novel ternary deep eutectic solvents pretreatment of corn stalk to realize high-value utilization. Chem. Eng. J..

[216] Xian X., Fang L., Zhou Y., Li B., Zheng X., Liu Y., Lin X. J. F. (2022). Integrated bioprocess for cellulosic ethanol production from wheat straw: new ternary deep-eutectic-solvent pretreatment, enzymatic saccharification, and fermentation. Fermentation.

[217] Li N., Meng F., Yang H., Shi Z., Zhao P., Yang J. (2022). Enhancing enzymatic digestibility of bamboo residues using a three-constituent deep eutectic solvent pretreatment. Bioresour. Technol..

[218] Zhu Y., Yang T. X., Qi B. K., Li H., Zhao Q. S., Zhao B. (2023). Acidic and alkaline deep eutectic solvents (DESs) pretreatment of grapevine: Component analysis, characterization, lignin structural analysis, and antioxidant properties. Int. J. Biol. Macromol..

[219] Tan Y. T., Ngoh G. C., Chua A. S. M. (2018). Evaluation of fractionation and delignification efficiencies of deep eutectic solvents on oil palm empty fruit bunch. Ind. Crops Prod..

[220] Rajeswari G., Jacob S. (2024). Co-fermentation of lactic acid and acetone-butanol-ethanol (ABE) from the deep eutectic solvent-pretreated Aloe vera leaf rind through sequential valorization of holocellulose. Biomass Convers Biorefin..

[221] Vigier K. D. O., Chatel G., Jérôme F. (2015). Contribution of deep eutectic solvents for biomass processing: opportunities, challenges, and limitations. ChemCatChem..

[222] Dietz C. H. J. T., Kroon M. C., van Sint Annaland M., Gallucci F. (2017). Thermophysical properties and solubility of different sugar-derived molecules in deep eutectic solvents. J. Chem. Eng. Data.

[223] Pu Y., Ziemer C., Ragauskas A. (2006). CP/MAS 13C NMR analysis of cellulase treated bleached softwood kraft pulp. Carbohydr. Res..

[224] Samuel R., Pu Y., Foston M., Ragauskas A. (2010). Solid-state NMR characterization of switchgrass cellulose after dilute acid pretreatment. Biofuels.

[225] Salem K. S., Kasera N. K., Rahman M. A., Jameel H., Habibi Y., Eichhorn S. J., French A. D., Pal L., Lucia L. A. (2023). Comparison and assessment of methods for cellulose crystallinity determination. Chem. Soc. Rev..

[226] Ling Z., Guo Z., Huang C., Yao L., Xu F. (2020). Deconstruction of oriented crystalline cellulose by novel levulinic acid based deep eutectic solvents pretreatment for improved enzymatic accessibility. Bioresour. Technol..

[227] Procentese A., Johnson E., Orr V., Campanile A. G., Wood J. A., Marzocchella A., Rehmann L. (2015). Deep eutectic solvent pretreatment and subsequent saccharification of corncob. Bioresour. Technol..

[228] Nor N. A. M., Mustapha W. A. W., Hassan O. (2016). Deep eutectic solvent (DES) as a pretreatment for oil palm empty fruit bunch (OPEFB) in sugar production. Procedia Chem..

[229] Pan M., Zhao G., Ding C., Wu B., Lian Z., Lian H. (2017). Physicochemical transformation of rice straw after pretreatment with a deep eutectic solvent of choline chloride/urea. Carbohydr. Polym..

[230] Zhang Q., Benoit M., De Oliveira Vigier K., Barrault J., Jérôme F. (2012). Green and inexpensive choline-derived solvents for cellulose decrystallization. Chem. Eur. J..

[900] Hertel, M. ; Bommarius, A. ; Realff, M. ; Kang, Y. Deep Eutectic Solvent Systems and Methods. World Intellectual Property Organization. WO 2012/145522, 2012.

[232] Seiler E. R. D., Takeoka Y., Rikukawa M., Yoshizawa-Fujita M. (2020). Development of a novel cellulose solvent based on pyrrolidinium hydroxide and reliable solubility analysis. RSC Adv..

[233] Lethesh K. C., Evjen S., Venkatraman V., Shah S. N., Fiksdahl A. (2020). Highly efficient cellulose dissolution by alkaline ionic liquids. Carbohydr. Polym..

[234] Acharya S., Liyanage S., Parajuli P., Rumi S. S., Shamshina J. L., Abidi N. (2021). Utilization of Cellulose to Its Full Potential: A Review on Cellulose Dissolution, Regeneration, and Applications. Polymers.

[235] Lu B., Xu A., Wang J. (2014). Cation does matter: how cationic structure affects the dissolution of cellulose in ionic liquids. Green Chem..

[236] Häkkinen R., Abbott A. (2019). Solvation of carbohydrates in five choline chloride-based deep eutectic solvents and the implication for cellulose solubility. Green Chem..

[237] Zhang H., Lang J., Lan P., Yang H., Lu J., Wang Z. (2020). Study on the Dissolution Mechanism of Cellulose by ChCl-Based Deep Eutectic Solvents. Materials (Basel).

[238] Ren H., Chen C., Wang Q., Zhao D., Guo S. (2016). The properties of choline chloride-based deep eutectic solvents and their performance in the dissolution of cellulose. BioResources.

[239] Malaeke H., Housaindokht M. R., Monhemi H., Izadyar M. (2018). Deep eutectic solvent as an efficient molecular liquid for lignin solubilization and wood delignification. J. Mol. Liq..

[240] Wang J., Wang Y., Ma Z., Yan L. J. G. E. (2020). Environment. Dissolution of highly molecular weight cellulose isolated from wheat straw in deep eutectic solvent of Choline/l-Lysine hydrochloride. Green Energy Environ..

[241] Zhang C. W., Xia S. Q., Ma P. (2016). Facile pretreatment of lignocellulosic biomass using deep eutectic solvents. Bioresour. Technol..

[242] Hou X. D., Smith T. J., Li N., Zong M. H. J. B. (2012). bioengineering. Novel renewable ionic liquids as highly effective solvents for pretreatment of rice straw biomass by selective removal of lignin. Biotechnol. Bioeng..

[243] Fang C., Thomsen M. H., Frankær C. G., Brudecki G. P., Schmidt J. E., AlNashef I. M. (2017). Reviving pretreatment effectiveness of deep eutectic solvents on lignocellulosic date palm residues by prior recalcitrance reduction. Ind. Eng. Chem. Res..

[244] Zhao Z., Chen X., Ali M. F., Abdeltawab A. A., Yakout S. M., Yu G. (2018). Pretreatment of wheat straw using basic ethanolamine-based deep eutectic solvents for improving enzymatic hydrolysis. Bioresour. Technol..

[245] Qaseem M. F., Shaheen H., Wu A.-M. (2021). Cell wall hemicellulose for sustainable industrial utilization. Renew. Sustain. Energy Rev..

[246] Liu Y., Chen W., Xia Q., Guo B., Wang Q., Liu S., Liu Y., Li J., Yu H. (2017). Efficient cleavage of lignin-carbohydrate complexes and ultrafast extraction of lignin oligomers from wood biomass by microwave-assisted treatment with deep eutectic solvent. ChemSusChem.

[247] Giummarella N., Pu Y., Ragauskas A. J., Lawoko M. J. G. C. (2019). A critical review on the analysis of lignin carbohydrate bonds. Gree Chem..

[248] Hou X. D., Feng G. J., Ye M., Huang C. M., Zhang Y. (2017). Significantly enhanced enzymatic hydrolysis of rice straw via a high-performance two-stage deep eutectic solvents synergistic pretreatment. Bioresour. Technol..

[249] Chen Z., Wang Y., Cheng H., Zhou H. (2022). Hemicellulose degradation: An overlooked issue in acidic deep eutectic solvents pretreatment of lignocellulosic biomass. Ind. Crop. Prod..

[250] Qin H., Hu X., Wang J., Cheng H., Chen L., Qi Z. (2020). Overview of acidic deep eutectic solvents on synthesis, properties and applications. Green Energy Environ..

[251] Zhong L., Wang C., Yang G., Chen J., Xu F., Yoo C. G., Lyu G. (2022). Rapid and efficient microwave-assisted guanidine hydrochloride deep eutectic solvent pretreatment for biological conversion of castor stalk. Bioresour. Technol..

[252] Huang C., Zhan Y., Cheng J., Wang J., Meng X., Zhou X., Fang G., Ragauskas A. J. (2021). Facilitating enzymatic hydrolysis with a novel guaiacol-based deep eutectic solvent pretreatment. Bioresour. Technol..

[253] Kim K. H., Dutta T., Sun J., Simmons B., Singh S. (2018). Biomass pretreatment using deep eutectic solvents from lignin derived phenols. Green Chem..

[254] Wang Y., Meng X., Jeong K., Li S., Leem G., Kim K. H., Pu Y., Ragauskas A. J., Yoo C. G. (2020). Investigation of a Lignin-Based Deep Eutectic Solvent Using p-Hydroxybenzoic Acid for Efficient Woody Biomass Conversion. ACS Sustain. Chem. Eng..

[255] Ryu J., Zhang M., Wang Y., Li R., Kim K. H., Ragauskas A. J., Leem G., Park M. B., Yoo C. G. (2024). Impacts of Hydrogen Bond Donor Structures in Phenolic Aldehyde Deep Eutectic Solvents on Pretreatment Efficiency. Energy Fuels.

[256] Xia Q., Liu Y., Meng J., Cheng W., Chen W., Liu S., Liu Y., Li J., Yu H. J. G. c. (2018). Multiple hydrogen bond coordination in three-constituent deep eutectic solvents enhances lignin fractionation from biomass. Green Chem..

[257] Huang C., Cheng J., Zhan Y., Liu X., Wang J., Wang Y., Yoo C. G., Fang G., Meng X., Ragauskas A. J. (2022). Utilization of guaiacol-based deep eutectic solvent for achieving a sustainable biorefinery. Bioresour. Technol..

[258] Wang Z. K., Li H., Lin X. C., Tang L., Chen J. J., Mo J.-W., Yu R.-S., Shen X. (2020). Novel recyclable deep eutectic solvent boost biomass pretreatment for enzymatic hydrolysis. Bioresour. Technol..

[259] Lin W., Xing S., Jin Y., Lu X., Huang C., Yong Q. (2020). Insight into understanding the performance of deep eutectic solvent pretreatment on improving enzymatic digestibility of bamboo residues. Bioresour. Technol..

[260] Lee C. B. T. L., Wu T. Y., Ting C. H., Tan J. K., Siow L. F., Cheng C. K., Md. Jahim J., Mohammad A. W. (2019). One-pot furfural production using choline chloride-dicarboxylic acid based deep eutectic solvents under mild conditions. Bioresour. Technol..

[261] Isci A., Thieme N., Lamp A., Zverlov V., Kaltschmitt M. (2021). Production of xylo-oligosaccharides from wheat straw using microwave assisted deep eutectic solvent pretreatment. Bioresour. Technol. Rep..

[262] Morais E. S., Freire M. G., Freire C. S., Coutinho J. A., Silvestre A. (2020). Enhanced conversion of xylan into furfural using acidic deep eutectic solvents with dual solvent and catalyst behavior. ChemSusChem..

[263] Chen Z., Bai X., A L., Wan C. (2018). High-solid lignocellulose processing enabled by natural deep eutectic solvent for lignin extraction and industrially relevant production of renewable chemicals. ACS Sustain. Chem. Eng..

[264] Jiang S., Verrier C., Ahmar M., Lai J., Ma C., Muller E., Queneau Y., Pera-Titus M., Jerome F., De Oliveira Vigier K. (2018). Unveiling the role of choline chloride in furfural synthesis from highly concentrated feeds of xylose. ACS Sustain. Chem. Eng..

[265] Cheng J., Huang C., Zhan Y., Liu X., Wang J., Huang C., Fang G., Ragauskas A. J., Xie Z., Meng X. (2023). A novel mineral-acid free biphasic deep eutectic solvent/γ-valerolactone system for furfural production and boosting the enzymatic hydrolysis of lignocellulosic biomass. Bioresour. Technol..

[266] Chen Z., Wan C. (2019). A novel deep eutectic solvent/acetone biphasic system for high-yield furfural production. Bioresour. Technol. Rep..

[267] Sun L. L., Yue Z., Sun S. C., Sun S. N., Cao X. F., Yuan T. Q., Wen J. L. (2022). Exploration of deep eutectic solvent-based biphasic system for furfural production and enhancing enzymatic hydrolysis: Chemical, topochemical, and morphological changes. Bioresour. Technol.y.

[268] Guo Z., Zhang Q., You T., Zhang X., Xu F., Wu Y. (2019). Short-time deep eutectic solvent pretreatment for enhanced enzymatic saccharification and lignin valorization. Green Chem..

[269] Zhai Q., Long F., Jiang X., Hse C., Jiang J., Xu J. (2020). Facile and rapid fractionation of bamboo wood with a p-toluenesulfonic acid-based three-constituent deep eutectic solvent. Ind. Crops Prod..

[270] Chen Z., Wan C. (2018). Ultrafast fractionation of lignocellulosic biomass by microwave-assisted deep eutectic solvent pretreatment. Bioresour. Technol..

[271] Lin X., Liu Y., Zheng X., Qureshi N. (2021). High-efficient cellulosic butanol production from deep eutectic solvent pretreated corn stover without detoxification. Ind. Crops Prod..

[272] Kumar A. K., Shah E., Patel A., Sharma S., Dixit G. (2018). Physico-chemical characterization and evaluation of neat and aqueous mixtures of choline chloride+ lactic acid for lignocellulosic biomass fractionation, enzymatic hydrolysis and fermentation. J. Mol. Liq..

[273] Chen Z., Jacoby W. A., Wan C. (2019). Ternary deep eutectic solvents for effective biomass deconstruction at high solids and low enzyme loadings. Bioresour. Technol..

[274] Yu Q., Zhang A., Wang W., Chen L., Bai R., Zhuang X., Wang Q., Wang Z., Yuan Z. J. B. t. (2018). Deep eutectic solvents from hemicellulose-derived acids for the cellulosic ethanol refining of Akebia’herbal residues. Bioresour. Technol..

[275] Liu Y., Zheng J., Xiao J., He X., Zhang K., Yuan S., Peng Z., Chen Z., Lin X. (2019). Enhanced enzymatic hydrolysis and lignin extraction of wheat straw by triethylbenzyl ammonium chloride/lactic acid-based deep eutectic solvent pretreatment. ACS omega.

[276] Ma C. Y., Xu L. H., Zhang C., Guo K. N., Yuan T.-Q., Wen J.-L. (2021). A synergistic hydrothermal-deep eutectic solvent (DES) pretreatment for rapid fractionation and targeted valorization of hemicelluloses and cellulose from poplar wood. Bioresour. Technol..

[277] Ling R., Wu W., Yuan Y., Wei W., Jin Y. (2021). Investigation of choline chloride-formic acid pretreatment and Tween 80 to enhance sugarcane bagasse enzymatic hydrolysis. Bioresour. Technol..

[278] Peng J., Xu H., Wang W., Kong Y., Su Z., Li B. (2021). Techno-economic analysis of bioethanol preparation process via deep eutectic solvent pretreatment. Ind. Crops Prod..

[279] Shen X. J., Wen J. L., Mei Q. Q., Chen X., Sun D., Yuan T. Q., Sun R. C. (2019). Facile fractionation of lignocelluloses by biomass-derived deep eutectic solvent (DES) pretreatment for cellulose enzymatic hydrolysis and lignin valorization. Green Chem..

[280] Chen Z., Bai X., Lusi A., Jacoby W. A., Wan C. (2019). One-pot selective conversion of lignocellulosic biomass into furfural and co-products using aqueous choline chloride/methyl isobutyl ketone biphasic solvent system. Bioresour. Technol..

[281] Ho M. C., Wu T. Y. (2020). Sequential pretreatment with alkaline hydrogen peroxide and choline chloride: copper (II) chloride dihydrate-Synergistic fractionation of oil palm fronds. Bioresour. Technol..

[282] Zhang Z., Xu J., Xie J., Zhu S., Wang B., Li J., Chen K. (2022). Physicochemical transformation and enzymatic hydrolysis promotion of reed straw after pretreatment with a new deep eutectic solvent. Carbohydr. Polym..

[283] Xu H., Kong Y., Peng J., Song X., Liu Y., Su Z., Li B., Gao C., Tian W. (2021). Comprehensive analysis of important parameters of choline chloride-based deep eutectic solvent pretreatment of lignocellulosic biomass. Bioresour. Technol..

[284] Provost V., Dumarcay S., Ziegler-Devin I., Boltoeva M., Trébouet D., Villain-Gambier M. J. B. t. (2022). Deep eutectic solvent pretreatment of biomass: Influence of hydrogen bond donor and temperature on lignin extraction with high β-O-4 content. Bioresour. Technol..

[285] Kumar N., Gautam R., Stallings J. D., Coty G. G., Lynam J. G. (2021). Secondary Agriculture Residues Pretreatment Using Deep Eutectic Solvents. WASTE BIOMASS VALORI..

[286] Sosa F. H. B., Abranches D. O., da Costa Lopes A. M., da Costa M. C., Coutinho J. A. P. (2024). Role of Deep Eutectic Solvent Precursors as Hydrotropes: Unveiling Synergism/Antagonism for Enhanced Kraft Lignin Dissolution. ACS Sustain. Chem. Eng..

[287] Song Y., Shi X., Ma S., Yang X., Zhang X. (2020). A novel aqueous gallic acid-based natural deep eutectic solvent for delignification of hybrid poplar and enhanced enzymatic hydrolysis of treated pulp. Cellulose.

[288] Kim K. H., Eudes A., Jeong K., Yoo C. G., Kim C. S., Ragauskas A. (2019). Integration of renewable deep eutectic solvents with engineered biomass to achieve a closed-loop biorefinery. Proceedings of the Proc. Nat. Acad. Sci. U.S.A.

[289] Chen L., Yu Q., Wang Q., Wang W., Qi W., Zhuang X., Wang Z., Yuan Z. (2019). A novel deep eutectic solvent from lignin-derived acids for improving the enzymatic digestibility of herbal residues from cellulose. Cellulose.

[290] Chen Y., Zhang L., Yu J., Lu Y., Jiang B., Fan Y., Wang Z. (2019). High-purity lignin isolated from poplar wood meal through dissolving treatment with deep eutectic solvents. R. Soc. Open Sci..

[291] Chen Z., Bai X., A L., Zhang H., Wan C. (2020). Insights into Structural Changes of Lignin toward Tailored Properties during Deep Eutectic Solvent Pretreatment. ACS Sustain. Chem. Eng..

[292] Tan Y. T., Ngoh G. C., Chua A. S. M. J. (2019). Effect of functional groups in acid constituent of deep eutectic solvent for extraction of reactive lignin. Bioresour. Technol..

[293] Wang Z. K., Hong S., Wen J.l., Ma C. Y., Tang L., Jiang H., Chen J. J., Li S., Shen X. J., Yuan T. Q. (2020). Lewis acid-facilitated deep eutectic solvent (DES) pretreatment for producing high-purity and antioxidative lignin. ACS Sustain. Chem. Eng..

[294] Guo Z., Zhang Q., You T., Ji Z., Zhang X., Qin Y., Xu F. (2019). Heteropoly acids enhanced neutral deep eutectic solvent pretreatment for enzymatic hydrolysis and ethanol fermentation of Miscanthus x giganteus under mild conditions. Bioresour. Technol..

[295] Alizadeh V., Malberg F., Pádua A. A. H., Kirchner B. (2020). Are There Magic Compositions in Deep Eutectic Solvents? Effects of Composition and Water Content in Choline Chloride/Ethylene Glycol from Ab Initio Molecular Dynamics. J. Phys. Chem. B.

[296] Dai Y., Witkamp G.-J., Verpoorte R., Choi Y. H. (2015). Tailoring properties of natural deep eutectic solvents with water to facilitate their applications. Food Chem..

[297] Zhang L., Zhang C., Ma Y., Zhao X., Zhang X. (2024). Lignocellulose pretreatment by Deep eutectic solvent and water binary system for enhancement of lignin extraction and cellulose saccharification. Ind. Crop. Prod..

[298] Fakayode O. A., Aboagarib E. A. A., Yan D., Li M., Wahia H., Mustapha A. T., Zhou C., Ma H. (2020). Novel two-pot approach ultrasonication and deep eutectic solvent pretreatments for watermelon rind delignification: Parametric screening and optimization via response surface methodology. Energy.

[299] Calcio Gaudino E., Tabasso S., Grillo G., Cravotto G., Dreyer T., Schories G., Altenberg S., Jashina L., Telysheva G. (2018). Wheat straw lignin extraction with bio-based solvents using enabling technologies. CR CHIM.

[300] Ji Q., Yu X., Yagoub A. E.-G. A., Chen L., Zhou C. J. (2020). Efficient removal of lignin from vegetable wastes by ultrasonic and microwave-assisted treatment with ternary deep eutectic solvent. Ind. Crops Prod.

[301] Muley P. D., Mobley J. K., Tong X., Novak B., Stevens J., Moldovan D., Shi J., Boldor D. (2019). Rapid microwave-assisted biomass delignification and lignin depolymerization in deep eutectic solvents. Energy Convers. Manage..

[302] Vaid S., Sharma S., Dutt H. C., Mahajan R., Bajaj B. K. (2021). One pot consolidated bioprocess for conversion of Saccharum spontaneum biomass to ethanol-biofuel. Energy Convers. Manage..

[303] Nargotra P., Sharma V., Bajaj B. K. (2019). Consolidated bioprocessing of surfactant-assisted ionic liquid-pretreated Parthenium hysterophorus L. biomass for bioethanol production. Bioresour. Technol..

[304] Yang Y. T., Qin M. K., Sun Q., Gao Y. F., Ma C. Y., Wen J. L. (2022). Structural elucidation and targeted valorization of poplar lignin from the synergistic hydrothermal-deep eutectic solvent pretreatment. Int. J. Biol. Macromol..

[305] Sun Q., Chen W. J., Pang B., Sun Z., Lam S. S., Sonne C., Yuan T. Q. (2021). Ultrastructural change in lignocellulosic biomass during hydrothermal pretreatment. Bioresour. Technol..

[306] Wang R., Wang K., Zhou M., Xu J., Jiang J. (2021). Efficient fractionation of moso bamboo by synergistic hydrothermal-deep eutectic solvents pretreatment. Bioresour. Technol..

[307] Wu Y., Liu Z., Ma L., Guo Z., Yang Q., Wang X., Hui L. (2021). Structural characterization and evaluation of the antioxidant activity of DES-Lignin isolated from Cunninghamia lanceolata. WOOD SCI TECHNOL.

[308] Li C., Huang C., Zhao Y., Zheng C., Su H., Zhang L., Luo W., Zhao H., Wang S., Huang L. (2021). Effect of choline-based deep eutectic solvent pretreatment on the structure of cellulose and lignin in bagasse. Processes.

[309] Schutyser W., Renders a. T., Van den Bosch S., Koelewijn S. F., Beckham G., Sels B. F. (2018). Chemicals from lignin: an interplay of lignocellulose fractionation, depolymerisation, and upgrading. Chem. Soc. Rev..

[310] Liu X., Li T., Wu S., Ma H., Yin Y. (2020). Structural characterization and comparison of enzymatic and deep eutectic solvents isolated lignin from various green processes: Toward lignin valorization. Bioresour. Technol..

[311] Xu F., Sun J., Wehrs M., Kim K. H., Rau S. S., Chan A. M., Simmons B. A., Mukhopadhyay A., Singh S. (2018). Biocompatible choline-based deep eutectic solvents enable one-pot production of cellulosic ethanol. ACS Sustain. Chem. Eng..

[312] Jablonský M., Škulcová A., Kamenská L., Vrška M., Šima J. (2015). Deep eutectic solvents: fractionation of wheat straw. Bioresources.

[313] Lou R., Ma R., Lin K. T., Ahamed A., Zhang X. (2019). Facile extraction of wheat straw by deep eutectic solvent (DES) to produce lignin nanoparticles. ACS Sustain. Chem. Eng..

[314] Hou X. D., Lin K. P., Li A. L., Yang L. M., Fu M. H. (2018). Effect of constituents molar ratios of deep eutectic solvents on rice straw fractionation efficiency and the micro-mechanism investigation. Ind. Crops Prod.

[315] Alvarez-Vasco C., Ma R., Quintero M., Guo M., Geleynse S., Ramasamy K. K., Wolcott M., Zhang X. (2016). Unique low-molecular-weight lignin with high purity extracted from wood by deep eutectic solvents (DES): a source of lignin for valorization. Green Chem..

[316] Soares B., da Costa Lopes A. M., Silvestre A. J.D., Rodrigues Pinto P. C., Freire C. S.R., Coutinho J. A.P. (2021). Wood delignification with aqueous solutions of deep eutectic solvents. Ind. Crops Prod.

[317] Wang S., Su S., Xiao L. P., Wang B., Sun R. C., Song G. (2020). Catechyl lignin extracted from castor seed coats using deep eutectic solvents: characterization and depolymerization. ACS Sustain. Chem. Eng..

[318] Das L., Li M., Stevens J., Li W., Pu Y., Ragauskas A. J., Shi J. (2018). Characterization and catalytic transfer hydrogenolysis of deep eutectic solvent extracted sorghum lignin to phenolic compounds. ACS Sustain. Chem. Eng..

[319] Li T., Lyu G., Liu Y., Lou R., Lucia L. A., Yang G., Chen J., Saeed H. A. (2017). Deep eutectic solvents (DESs) for the isolation of willow lignin (Salix matsudana cv. Zhuliu). Int. J. Mol. Sci..

[320] Li W., Amos K., Li M., Pu Y., Debolt S., Ragauskas A. J., Shi J. (2018). Fractionation and characterization of lignin streams from unique high-lignin content endocarp feedstocks. Biotechnol. biofuels bioprod..

[321] Xie J., Chen J., Cheng Z., Zhu S., Xu J. J. C. P. (2021). Pretreatment of pine lignocelluloses by recyclable deep eutectic solvent for elevated enzymatic saccharification and lignin nanoparticles extraction. Carbohydr. Polym..

[322] Kim K. H., Eudes A., Jeong K., Yoo C. G., Kim C. S., Ragauskas A. (2019). Integration of renewable deep eutectic solvents with engineered biomass to achieve a closed-loop biorefinery. Proc. Nat. Acad. Sci. U.S.A..

[323] Satlewal A., Agrawal R., Das P., Bhagia S., Pu Y., Puri S. K., Ramakumar S., Ragauskas A. (2019). Assessing the facile pretreatments of bagasse for efficient enzymatic conversion and their impacts on structural and chemical properties. ACS Sustain. Chem. Eng..

[324] Fernandes C., Melro E., Magalhães S., Alves L., Craveiro R., Filipe A., Valente A. J., Martins G., Antunes F. E., Romano A. (2021). New deep eutectic solvent assisted extraction of highly pure lignin from maritime pine sawdust (Pinus pinaster Ait.). Int. J. Biol. Macromol..

[325] Njoku S. I., Ahring B. K., Uellendahl H. (2012). Pretreatment as the crucial step for a cellulosic ethanol biorefinery: testing the efficiency of wet explosion on different types of biomass. Bioresour. Technol..

[326] Maitan-Alfenas G. P., Visser E. M., Guimarães V. M. (2015). Enzymatic hydrolysis of lignocellulosic biomass: converting food waste in valuable products. Curr. Opin. Food Sci..

[327] Zhang Z., Liu B., Zhao Z. K. (2012). Efficient acid-catalyzed hydrolysis of cellulose in organic electrolyte solutions. Polym. Degrad. Stab..

[328] Brummer V., Skryja P., Jurena T., Hlavacek V., Stehlik P. (2014). Suitable technological conditions for enzymatic hydrolysis of waste paper by Novozymes® Enzymes NS50013 and NS50010. Appl. Biochem. Biotechnol..

[329] Poovaiah C. R., Nageswara-Rao M., Soneji J. R., Baxter H. L., Stewart C. N. (2014). Altered lignin biosynthesis using biotechnology to improve lignocellulosic biofuel feedstocks. Plant Biotechnol.

[330] Kulshrestha A., Pancha I., Mishra S., Kumar A. J. (2021). Deep eutectic solvents and ionic liquid assisted hydrolysis of microalgal biomass: A promising approach towards sustainable biofuel production. J. Mol. Liq..

[331] Xu F., Sun J., Konda N. M., Shi J., Dutta T., Scown C. D., Simmons B. A., Singh S. (2016). Transforming biomass conversion with ionic liquids: process intensification and the development of a high-gravity, one-pot process for the production of cellulosic ethanol. Energy Environ. Sci..

[332] Gorke J. T., Srienc F., Kazlauskas R. J. (2008). Hydrolase-catalyzed biotransformations in deep eutectic solvents. Chem. Commun..

[333] Nagoor Gunny A., Arbain D., Mohamed Daud M., Jamal P. (2014). Synergistic action of deep eutectic solvents and cellulases for lignocellulosic biomass hydrolysis. Mater. Res. Innovations.

[334] Nargotra P., Sharma V., Sharma S., Kapoor N., Bajaj B. K. (2022). Development of consolidated bioprocess for biofuel-ethanol production from ultrasound-assisted deep eutectic solvent pretreated Parthenium hysterophorus biomass. Biomass Convers. Biorefin..

[335] Wahlström R., Hiltunen J., Sirkka M. P. d. S. N., Vuoti S., Kruus K. (2016). Comparison of three deep eutectic solvents and 1-ethyl-3-methylimidazolium acetate in the pretreatment of lignocellulose: effect on enzyme stability, lignocellulose digestibility and one-pot hydrolysis. RSC Adv..

[336] Gunny A. A. N., Arbain D., Nashef E. M., Jamal P. (2015). Applicability evaluation of Deep Eutectic Solvents-Cellulase system for lignocellulose hydrolysis. Bioresour. Technol..

[337] Zulkefli S., Abdulmalek E., Rahman M. B. A. (2017). Pretreatment of oil palm trunk in deep eutectic solvent and optimization of enzymatic hydrolysis of pretreated oil palm trunk. Renew. Energy.

[338] Casey E., Mosier N. S., Adamec J., Stockdale Z., Ho N., Sedlak M. (2013). Effect of salts on the Co-fermentation of glucose and xylose by a genetically engineered strain of Saccharomyces cerevisiae. Biotechnol. biofuels bioprod..

[339] Lin T. H., Huang C. F., Guo G. L., Hwang W.-S., Huang S. L. (2012). Pilot-scale ethanol production from rice straw hydrolysates using xylose-fermenting Pichia stipitis. Bioresour. Technol..

[340] Wirawan F., Cheng C. L., Kao W. C., Lee D. J., Chang J. S. (2012). Cellulosic ethanol production performance with SSF and SHF processes using immobilized Zymomonas mobilis. Appl. Energy.

[341] Hasunuma T., Kondo A. (2012). Development of yeast cell factories for consolidated bioprocessing of lignocellulose to bioethanol through cell surface engineering. Biotechnol. Adv..

[342] Okuofu S. I., Gerrano A. S., Singh S., Pillai S. (2022). Deep eutectic solvent pretreatment of Bambara groundnut haulm for enhanced saccharification and bioethanol production. Biomass Convers. Biorefin..

[343] Ngatcha A. D. P., Muhammad G., Lv Y., Xiong W., Zhao A., Xu J., Alam M. A. (2022). Microalgae biomass pre-treatment with deep eutectic solvent to optimize lipid isolation in biodiesel production. Biomass Convers. Biorefin..

[344] Bhatia S. K., Otari S. V., Jeon J. M., Gurav R., Choi Y. K., Bhatia R. K., Pugazhendhi A., Kumar V., Banu J. R., Yoon J.J. J. B. T. (2021). Biowaste-to-Bioplastic (polyhydroxyalkanoates): Conversion technologies, strategies, challenges, and perspective. Bioresour. Technol..

[345] Gu L., Huang W., Tang S., Tian S., Zhang X. (2015). A novel deep eutectic solvent for biodiesel preparation using a homogeneous base catalyst. Chem. Eng. J..

[346] Liu W., Wang F. (2018). p-Toluenesulfonic Acid-based Deep Eutectic Solvent as Transesterification Catalyst for Biodiesel Production. J. Oleo Sci..

[347] Shahbaz K., Mjalli F. S., Hashim M. A., AlNashef I. M. (2011). Eutectic solvents for the removal of residual palm oil-based biodiesel catalyst. Sep. Purif. Technol..

[348] Pan Y., Alam M. A., Wang Z., Huang D., Hu K., Chen H., Yuan Z. (2017). One-step production of biodiesel from wet and unbroken microalgae biomass using deep eutectic solvent. Bioresour. Technol..

[349] Wichaphian A., Kaewman N., Pathom-Aree W., Phinyo K., Pekkoh J., Chromkaew Y., Cheirsilp B., Srinuanpan S. (2024). Zero-waste biorefining co-products from ultrasonically assisted deep eutectic solvent-pretreated Chlorella biomass: Sustainable production of biodiesel and bio-fertilizer. Bioresour. Technol..

[350] van Osch D. J., Kollau L. J., van den Bruinhorst A., Asikainen S., Rocha M. A., Kroon M. C. (2017). Ionic liquids and deep eutectic solvents for lignocellulosic biomass fractionation. Phys. Chem. Chem. Phys..

[351] Yan G., Zhou Y., Zhao L., Wang W., Yang Y., Zhao X., Chen Y., Yao X. (2022). Recycling of deep eutectic solvent for sustainable and efficient pretreatment of corncob. Ind. Crops and Prod..

[352] Ci Y. H., Yu F., Zhou C. X., Mo H. E., Li Z. Y., Ma Y. Q., Zang L. H. (2020). New ternary deep eutectic solvents for effective wheat straw deconstruction into its high-value utilization under near-neutral conditions. Green Chem..

[353] Zhu A., Jiang T., Han B., Zhang J., Xie Y., Ma X. (2007). Supported choline chloride/urea as a heterogeneous catalyst for chemical fixation of carbon dioxide to cyclic carbonates. Green Chem..

[354] Zhu A., Li L., Wang J., Zhuo K. (2011). Direct nucleophilic substitution reaction of alcohols mediated by a zinc-based ionic liquid. Green chem..

[355] Isci A., Kaltschmitt M. (2022). Recovery and recycling of deep eutectic solvents in biomass conversions: A review. Biomass Convers. Biorefin..

[356] Mamilla J. L., Novak U., Grilc M., Likozar B. (2019). Natural deep eutectic solvents (DES) for fractionation of waste lignocellulosic biomass and its cascade conversion to value-added bio-based chemicals. Biomass Bioenergy.

[357] Li A. L., Hou X. D., Lin K. P., Zhang X., Fu M. H. (2018). Rice straw pretreatment using deep eutectic solvents with different constituents molar ratios: Biomass fractionation, polysaccharides enzymatic digestion and solvent reuse. J. Biosci. Bioeng..

[358] Yu Q., Song Z., Zhuang X., Liu L., Qiu W., Shi J., Wang W., Li Y., Wang Z., Yuan Z. (2019). Catalytic conversion of herbal residue carbohydrates to furanic derivatives in a deep eutectic solvent accompanied by dissolution and recrystallisation of choline chloride. Cellulose.

[359] Zhang, L. ; Yu, H. Conversion of xylan and xylose into furfural in biorenewable deep eutectic solvent with trivalent metal chloride added. BioResources 2013. 10.15376/biores.8.4.6014-6025

[360] Le K., Zuo M., Song X., Zeng X., Tang X., Sun Y., Lei T., Lin L. J. (2017). An effective pathway for 5-brominemethylfurfural synthesis from biomass sugars in deep eutectic solvent. J. Chem. Technol. Biotechnol..

[361] Tran P. H., Tran P. V. (2019). A highly selective and efficient method for the production of 5-hydroxymethylfurfural from dehydration of fructose using SACS/DES catalytic system. Fuel.

[362] Lang J., Lu J., Lan P., Wang N., Yang H., Zhang H. (2020). Preparation of 5-HMF in a des/ethyl n-butyrate two-phase system. Catalysts.

[363] Merza F., Fawzy A., AlNashef I., Al-Zuhair S., Taher H. J. E. R. (2018). Effectiveness of using deep eutectic solvents as an alternative to conventional solvents in enzymatic biodiesel production from waste oils. Energy Rep..

[364] Xu M., Ran L., Chen N., Fan X., Ren D., Yi L. (2019). Polarity-dependent extraction of flavonoids from citrus peel waste using a tailor-made deep eutectic solvent. Food Chem..

[365] Smink D., Kersten S. R., Schuur B. (2020). Comparing multistage liquid-liquid extraction with cold water precipitation for improvement of lignin recovery from deep eutectic solvents. Sep. Purif. Technol..

[366] Grillo G., Gunjević V., Radošević K., Redovniković I. R., Cravotto G. (2020). Deep eutectic solvents and nonconventional technologies for blueberry-peel extraction: Kinetics, anthocyanin stability, and antiproliferative activity. Antioxidants.

[367] Plaza A., Tapia X., Yañez C., Vilches F., Candia O., Cabezas R., Romero J. (2020). Obtaining hydroxytyrosol from olive mill waste using deep eutectic solvents and then supercritical CO 2. Waste and Biomass Valori..

[368] Zhou J., Sui H., Jia Z., Yang Z., He L., Li X. (2018). Recovery and purification of ionic liquids from solutions: a review. RSC adv..

[369] Knez Ž., Markočič E., Leitgeb M., Primožič M., Hrnčič M. K., Škerget M. (2014). Industrial applications of supercritical fluids: A review. Energy.

[370] Hayyan A., Ali Hashim M., Mjalli F. S., Hayyan M., AlNashef I. M. (2013). A novel phosphonium-based deep eutectic catalyst for biodiesel production from industrial low grade crude palm oil. Chem. Eng. Sci..

[371] Hayyan A., Hashim M. A., Hayyan M., Mjalli F. S., AlNashef I. M. (2013). A novel ammonium based eutectic solvent for the treatment of free fatty acid and synthesis of biodiesel fuel. Ind. Crops Prod..

[372] Hayyan A., Hashim M. A., Hayyan M., Mjalli F. S., AlNashef I. M. (2014). A new processing route for cleaner production of biodiesel fuel using a choline chloride based deep eutectic solvent. J. Clean. Prod..

[373] Wahlström R., Rommi K., Willberg-Keyriläinen P., Ercili-Cura D., Holopainen-Mantila U., Hiltunen J., Mäkinen O., Nygren H., Mikkelson A., Kuutti L. (2017). High yield protein extraction from Brewer’s spent grain with novel carboxylate salt-urea aqueous deep eutectic solvents. ChemistrySelect.

[374] Liang X., Fu Y., Chang J. (2019). Effective separation, recovery and recycling of deep eutectic solvent after biomass fractionation with membrane-based methodology. Sep. Purif. Technol..

[375] Kumar A. K., Sharma S., Dixit G., Shah E., Patel A., Boczkaj G. (2020). Techno-economic evaluation of a natural deep eutectic solvent-based biorefinery: Exploring different design scenarios. Biofuels, Bioprod. Biorefin..

[376] Zhao J., Lee J., Wang D. (2022). An integrated deep eutectic solvent-ionic liquid-metal catalyst system for lignin and 5-hydroxymethylfurfural production from lignocellulosic biomass: Technoeconomic analysis. Bioresour. Technol..

[377] Zang G., Shah A., Wan C. (2020). Techno-economic analysis of co-production of 2, 3-butanediol, furfural, and technical lignin via biomass processing based on deep eutectic solvent pretreatment. Biofuels, Bioprod. Biorefin..

[378] Zargar S., Jiang J., Jiang F., Tu Q. (2022). Isolation of lignin-containing cellulose nanocrystals: life-cycle environmental impacts and opportunities for improvement. Biofuels, Bioprod. Biorefin..

[379] Guo T., Yu Y., Wan Z., Zargar S., Wu J., Bi R., Sokhansanj S., Tu Q., Rojas O. J. (2022). Energy pellets from whole-wheat straw processed with a deep eutectic solvent: A comprehensive thermal, molecular and environmental evaluation. Renew. Energy.

[380] Xia Q., Chen C., Yao Y., Li J., He S., Zhou Y., Li T., Pan X., Yao Y., Hu L. (2021). A strong, biodegradable and recyclable lignocellulosic Bioplastic. Nat. Sustain..

[381] Guo Y., Li Y., Li Z., Yan W., Chen P., Yao S. (2021). Extraction assisted by far infrared radiation and hot air circulation with deep eutectic solvent for bioactive polysaccharides from Poria cocos (Schw.) wolf. Green Chem..

[382] Murugan, M. ; Tee, L. ; Oh, K. Evaluation of the environment impact of extraction of bioactive compounds from Darcyodes rostrata using Deep Eutectic Solvent (DES) using Life Cycle Assessment (LCA). In Journal of Physics: Conference Series, 2021; p 012005.

[383] Ioannou I., D’Angelo S. C., Galán-Martín Á., Pozo C., Pérez-Ramírez J., Guillén-Gosálbez G. (2021). Process modelling and life cycle assessment coupled with experimental work to shape the future sustainable production of chemicals and fuels. React. Chem. Eng..

[384] Modal, I. ; Brekke, A. ; Raadal, H. Life cycle assessment of cellulose, ethanol, lignin and vanillin from Borregaard, Sarpsborg—phase II. Ostfold Research, OR 2009, 17.

[385] Modahl, I. ; Vold, B. The 2010 LCA of cellulose, ethanol, lignin and vanillin from Borregaard Sarpsborg. Ostfold Research, Norway 2011, 78.

[386] Cohen A. J., Mori-Sánchez P., Yang W. (2012). Challenges for Density Functional Theory. Chem. Rev..

[387] Mariano Colombari F., Marcos Nascimento V., Liu Y. L., de Morais Rocha G. J., Driemeier C. (2022). Density functional theory with implicit solvents for accurate estimation of aqueous and organic solvation free energies of lignin fragments. ACS Sustain. Chem. Eng..

[388] Houston R. W., Abdoulmoumine N. H. (2023). Investigation of the thermal deconstruction of β-β′ and 4-O-5 linkages in lignin model oligomers by density functional theory (DFT). RSC Adv..

[389] Shah M., Singh P. S., Priyanka N., Devi T. G. (2025). Synthesis and characterization of hydrophobic and hydrophilic deep eutectic solvents: Spectroscopic, DFT, molecular docking analysis. J. Mol. Liq..

[390] Han P., Nie W., Zhao G., Gao P. (2022). Theoretical investigation on the structure and physicochemical properties of choline chloride-based deep eutectic solvents. J. Mol. Liq..

[391] Pham, L. T. M. ; Choudhary, H. ; Gauttam, R. ; Singer, S. W. ; Gladden, J. M. ; Simmons, B. A. ; Singh, S. ; Sale, K. L. Revisiting Theoretical Tools and Approaches for the Valorization of Recalcitrant Lignocellulosic Biomass to Value-Added Chemicals. Front. energy res. 2022, 10. 10.3389/fenrg.2022.863153

[392] Pan X., Liu Y., Ma Z., Qin Y., Lu X., Feng X., Shao Q., Zhu Y. (2024). Molecular insight into the mechanism of lignin dissolution in acid choline chloride-Based deep eutectic solvents. J. Mol. Liq..

[393] Wang W., Zhu B., Xu Y., Li B., Xu H. (2022). Mechanism study of ternary deep eutectic solvents with protonic acid for lignin fractionation. Bioresour. Technol..

[394] Zhang Y., Ren H., Li B., Udin S. M., Maarof H., Zhou W., Cheng F., Yang J., Liu Y., Alias H. (2023). Mechanistic insights into the lignin dissolution behavior in amino acid based deep eutectic solvents. Int. J. Biol. Macromol..

[395] Madeira P. P., Passos H., Gomes J., Coutinho J. A., Freire M. G. (2017). Alternative probe for the determination of the hydrogen-bond acidity of ionic liquids and their aqueous solutions. Phys. Chem. Chem. Phys..

[396] Mohan M., Gugulothu N., Guggilam S., Rajeshwar T. R., Kidder M. K., Smith J. C. (2025). Physics-informed machine learning to predict solvatochromic parameters of designer solvents with case studies in CO2 and lignin dissolution. Green Chem. Eng..

[397] Li H., Li D., He Y., Luo C., Lan W., Ragauskas A. J., You T., Xu F. (2025). Unleashing the potential of lignin with innovative trifunctional binary deep eutectic solvent strategy: Isolation of uncondensed lignin. Sci. Adv..

[398] Qiao Q., Shi J., Shao Q. (2021). The multiscale solvation effect on the reactivity of β-O-4 of lignin dimers in deep eutectic solvents. Phys. Chem. Chem. Phys..

[399] Zhu B., Ge H., Wei J., Xu Y., Wang S., Li B., Xu H. (2024). Comprehensive analysis of lignin dimer dissolution microscopic mechanism in different aromatic-based deep eutectic solvent. Ind. Crops Prod..

[400] Li Q., Dong Y., Hammond K. D., Wan C. (2021). Revealing the role of hydrogen bonding interactions and supramolecular complexes in lignin dissolution by deep eutectic solvents. J. Mol. Liq..

[401] Ge H., Wei J., Wang S., Yan Z., Jiang M., Zhu L., Liu C., Li B., Huang C., Xu H. (2025). Multiscale exploration of the lignin dissolution mechanism based on novel ternary deep eutectic solvents incorporating p-hydroxybenzoic acid. Green Chemi..

[402] Xu H., Kong Y., Peng J., Wang W., Li B. (2021). Mechanism of deep eutectic solvent delignification: insights from molecular dynamics simulations. ACS Sustain. Chem. Eng..

[403] Mohan M., Naik P. K., Banerjee T., Goud V. V., Paul S. (2017). Solubility of glucose in tetrabutylammonium bromide based deep eutectic solvents: Experimental and molecular dynamic simulations. Fluid Ph. Equilib..

[404] Azougagh O., Jilal I., Jabir L., El-Hammi H., Essayeh S., Mohammed N., Achalhi N., El yousfi R., El Idrissi A., El Ouardi Y. (2023). Dissolution mechanism of cellulose in a benzyltriethylammonium/urea deep eutectic solvent (DES): DFT-quantum modeling, molecular dynamics and experimental investigation. Phys. Chem. Chem. Phys..

[405] Wang W., Kong Y., Peng J., Li B., Xu H. (2022). Probing the mechanism of green solvent solubilization of hemicellulose based on molecular dynamics simulations. Ind. Crop Prod.

[406] Yu Y., Wan Z., Parks J. M., Sokhansanj S., Rojas O. J., Smith J. C. J. G. C. (2024). Molecular origins of enhanced bioproduct properties by pretreatment of agricultural residues with deep eutectic solvents. Green Chem..

[407] Wang K., Peng D., Alhadid A., Minceva M. (2024). Assessment of COSMO-RS for Predicting Liquid-Liquid Equilibrium in Systems Containing Deep Eutectic Solvents. Ind. Eng. Chem. Res..

[408] Wojeicchowski J. P., Abranches D. O., Ferreira A. M., Mafra M. R., Coutinho J. A. P. (2021). Using COSMO-RS to Predict Solvatochromic Parameters for Deep Eutectic Solvents. ACS Sustain. Chem. Eng..

[409] Al-Maari M. A., Hizaddin H. F., Hayyan A., Hadj-Kali M. K. (2024). Screening deep eutectic solvents as green extractants for oil from plant seeds based on COSMO-RS model. J. Mol. Liq..

[410] Wojeicchowski J. P., Ferreira A. M., Abranches D. O., Mafra M. R., Coutinho J. A. P. (2020). Using COSMO-RS in the Design of Deep Eutectic Solvents for the Extraction of Antioxidants from Rosemary. ACS Sustain. Chem. Eng.g.

[411] Liu, Y. ; Yu, H. ; Sun, Y. ; Zeng, S. ; Zhang, X. ; Nie, Y. ; Zhang, S. ; Ji, X. Screening Deep Eutectic Solvents for CO2 Capture With COSMO-RS. Front. Chem. 2020, 8. 10.3389/fchem.2020.00082 PMC7031488 32117899

[412] Khan P., Yasin M., AlMohamadi H., Zhang X., Laeeq Khan A., Nawaz R., Amjad Gilani M. (2024). Exploring the potential of hydrophobic deep eutectic solvents for bioethanol separation using DFT and COSMO-RS model. J. Mol. Liq..

[413] Adeyemi I., Sulaiman R., Almazroui M., Al-Hammadi A., AlNashef I. M. (2020). Removal of chlorophenols from aqueous media with hydrophobic deep eutectic solvents: Experimental study and COSMO RS evaluation. J. Mol. Liq..

[414] Heintz A., Kulikov D. V., Verevkin S. P. (2001). Thermodynamic Properties of Mixtures Containing Ionic Liquids. 1. Activity Coefficients at Infinite Dilution of Alkanes, Alkenes, and Alkylbenzenes in 4-Methyl-n-butylpyridinium Tetrafluoroborate Using Gas-Liquid Chromatography. J. Chem. Eng. Data.

[415] Mukesh C., Huang G., Qin H., Liu Y., Ji X. (2024). Insight into lignin oxidative depolymerization in ionic liquids and deep eutectic solvents. Biomass Bioenerg..

[416] Balaji C., Banerjee T., Goud V. V. (2012). COSMO-RS Based Predictions for the Extraction of Lignin from Lignocellulosic Biomass Using Ionic Liquids: Effect of Cation and Anion Combination. J. Solution Chem..

[417] Zhou L., Meng X., Li W., Yu J., Kemefa C. O., Dai S. Y., Ragauskas A. J., Yuan J. S. (2025). Computational modeling-guided design of deep eutectic solvents for tailoring lignin chemistry during lignocellulose pretreatment. Green Chem..

[418] Hayyan M., Hashim M. A., Hayyan A., Al-Saadi M. A., AlNashef I. M., Mirghani M. E., Saheed O. K. J. C. (2013). Are deep eutectic solvents benign or toxic?. Chemosphere.

[419] Wen Q., Chen J.-X., Tang Y.-L., Wang J., Yang Z. J. C. (2015). Assessing the toxicity and biodegradability of deep eutectic solvents. Chemosphere.

[420] Van Osch D. J., Dietz C. H., Warrag S. E., Kroon M. C. (2020). The curious case of hydrophobic deep eutectic solvents: a story on the discovery, design, and applications. ACS Sustain. Chem. Eng..

[421] Amesho K. T., Lin Y.-C., Mohan S. V., Halder S., Ponnusamy V. K., Jhang S.-R. J. E. c. l. (2023). Deep eutectic solvents in the transformation of biomass into biofuels and fine chemicals: a review. Environ. Chem. Lett..

[422] Li P., Yang C., Jiang Z., Jin Y., Wu W. J. J. o. B. (2023). Bioproducts. Lignocellulose pretreatment by deep eutectic solvents and related technologies: A review. J. Bioresour. Bioprod..

[423] Kalhor P., Ghandi K. J. (2019). Deep eutectic solvents for pretreatment, extraction, and catalysis of biomass and food waste. Molecules.

[424] Lobato-Rodríguez Á., Gullón B., Romaní A., Ferreira-Santos P., Garrote G., Del-Río P. G. (2023). Recent advances in biorefineries based on lignin extraction using deep eutectic solvents: A review. Bioresour. Technol..

[425] Xiao T., Hou M., Guo X., Cao X., Li C., Zhang Q., Jia W., Sun Y., Guo Y., Shi H. (2024). Recent progress in deep eutectic solvent (DES) fractionation of lignocellulosic components: A review. Renew. Sustain. Energy Rev..

[426] Hussain M., Ashraf H. T., Vasudev V., Yasin S., Jamil M. I., Saleem M., Aziz T., Al Afroz E., Feichao Z., Huapeng Z. (2025). Deep eutectic solvents in biomass pretreatment for green approach: a comprehensive review. Polym. Bull..

